# Absolute chronology of the Early Palaeolithic Karatau Culture in Central Asia

**DOI:** 10.1038/s41467-026-74938-5

**Published:** 2026-07-01

**Authors:** Aske L. Sørensen, Anton A. Anoikin, Kseniya A. Kolobova, Tura U. Khudjageldiev, Petr M. Sosin, Natalia Taratunina, Ekaterina P. Kulakova, Olga Tokareva, Olga Meshcheryakova, Aljasil Chirakkal, Amélie Challier, Ramona Schneider, Asleddin Karayev, Islom Ashurmadov, Abdullo Sharipov, Vladimir Pavlov, Andrei Rybalko, Farhad Khormali, Andrew S. Murray, Gábor Ujvari, Jago J. Birk, Jesper Olsen, Thomas M. Hansen, Redzhep N. Kurbanov, Thomas Stevens, David K. Wright, Jan-Pieter Buylaert, Mads F. Knudsen

**Affiliations:** 1 Department of Geoscience, Aarhus University, Aarhus, Denmark; 2 Department of Geosciences and Natural Resource Management, University of Copenhagen, Copenhagen, Denmark; 3 Individual researcher, Novosibirsk, Russia; 4 APSACA, Institute of Anthropology, Tashkent, Uzbekistan; 5 Institute of History, Archaeology and Ethnography, National Academy of Science of Tajikistan, Dushanbe, Tajikistan; 6 Institute of Water Problems, Hydropower and Ecology, National Academy of Science of Tajikistan, Dushanbe, Tajikistan; 7 Department of Physics, Technical University of Denmark, DTU Risø Campus, Roskilde, Denmark; 8 Individual researcher, Moscow, Russia; 9 Department of Soil Sciences, Gorgan University of Agricultural Sciences and Natural Resources, Gorgan, Iran; 10 School of Geography and Ocean Sciences, Nanjing University, Nanjing, China; 11 Department of Archaeology, Conservation and History, University of Oslo, Oslo, Norway; 12 Department of Earth Sciences, Uppsala University, Uppsala, Sweden; 13 Department of Earth Sciences, Southern Methodist University, Dallas, Texas USA; 14 Institute for Geological and Geochemical Research, HUN-REN Research Centre for Astronomy and Earth Sciences, Budapest, Hungary; 15 CSFK, MTA Centre of Excellence, Budapest, Hungary; 16 Department of Geography, University of Göttingen, Göttingen, Germany; 17 Department of Physics, Aarhus University, Aarhus, Denmark

**Keywords:** Palaeoclimate, Stratigraphy, Archaeology

## Abstract

Central Asia represents a key region for our understanding of early human dispersal patterns, because it served as a migration corridor that linked the Levant and southern Caucasus with Northeast Asia. However, no Early Palaeolithic sites in Central Asia are anchored with reliable age constraints, including the thick loess-palaeosol sections in Tajikistan that recorded early human activities and environmental changes over multiple glacial-interglacial cycles. This lack of absolute age constraints is presently a key factor limiting our understanding of the early human occupation history of this region. Here, we provide a comprehensive description and an absolute chronological framework for the Early Palaeolithic Karatau Culture; defined by the rich lithic assemblages found in palaeosols in the Khovaling Loess Plateau, Tajikistan. Age constraints are provided through multi-method analysis of three loess-palaeosol sections in the Khovaling Loess Plateau, combining luminescence ages, cosmogenic ^26^Al-^10^Be concentrations, and magnetostratigraphic boundaries into a probabilistic inverse age-depth model. This model shows that the Karatau Culture flourished with the onset of Marine Isotope Stage 15, thrived during Marine Isotope Stage 13 and 11, but disappeared around onset of Marine Isotope Stage 10. We frame the archaeological occupations within local and regional ecological settings to better understand the drivers of Pleistocene human migrations in Central Asia.

## Abstract (web summary)

Early Palaeolithic sites in Central Asia are important for our understanding of early human dispersal and migration. Here, the authors present a multi-method, absolute age framework for the Karatau Culture, spanning the last 1 million years, in the Khovaling Loess Plateau, Tajikistan.

## Introduction

**Fig. 1:**
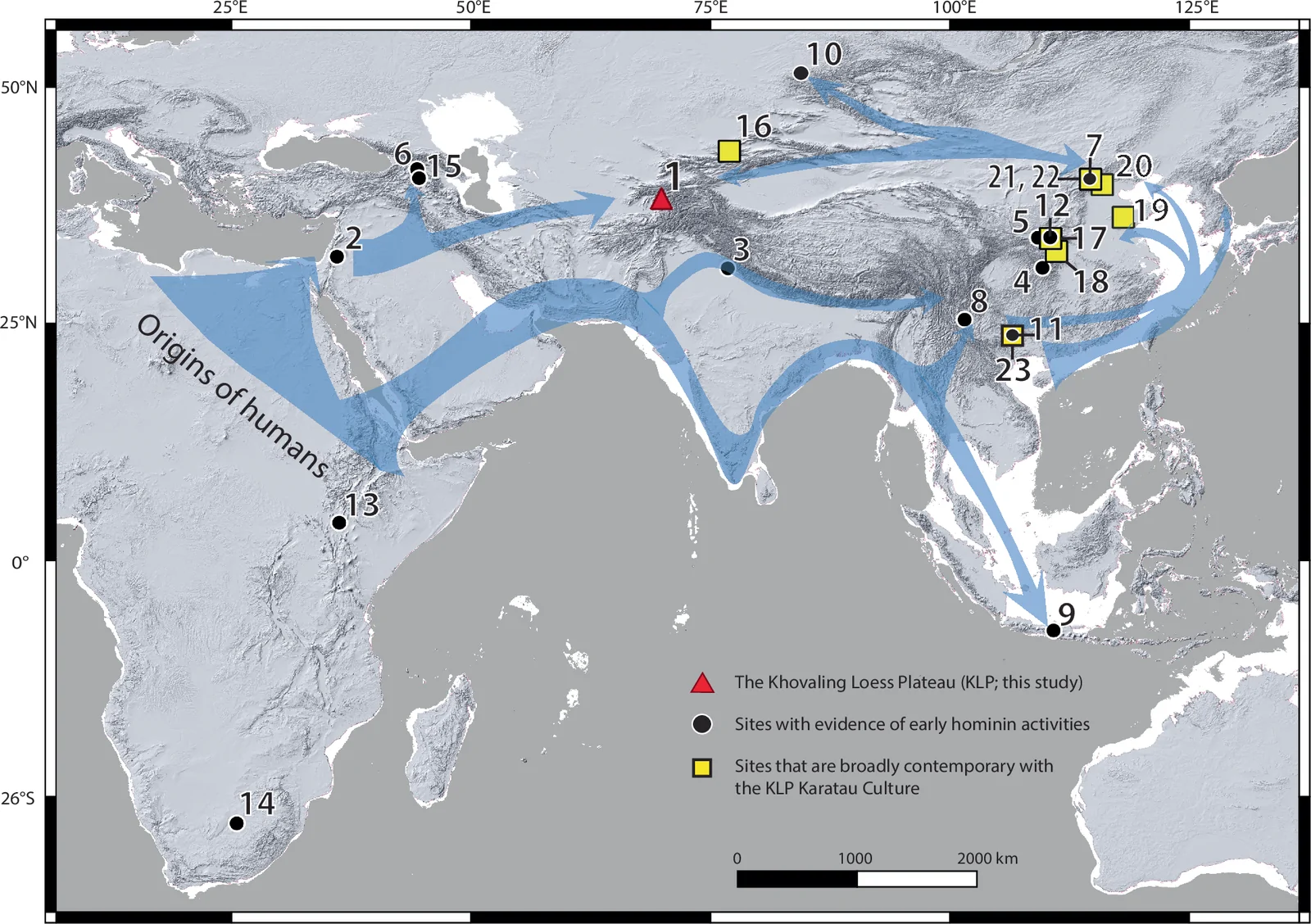
Potential migration routes of early hominins out of Africa across Asia. The topographic reconstruction^77^ shows global sea levels at maximum glacial conditions (white areas), i.e. −120 m from the present-day level^78^. Arrows represent potential migration routes based on^21,79–81^. The Khovaling Loess Plateau (KLP) (this study) is represented by a red triangle. Black dots represent sites with evidence of early hominin activities in various parts of Asia (sites 2–12) or early use of fire (sites 13–15). These sites include: 2. Sukhna^1^; 3. Masol^5^; 4. Longgupo^4^; 5. Lantian^7^; 6. Dmanisi^8^; 7. Nihewan Basin^9^; 8. Niujianbao^10^; 9. Sangirian^11^; 10. Karama^12,13^; 11. Bose Basin (Xiaomei and Fengshudao)^14^; 12. Luonan Basin^60^; 13. Koobi Fora^55^; 14. Wonderwerk Cave^56^; 15. Lusakert Cave^58^. Yellow squares denote sites that specifically are discussed in relation to the KLP Karatau Culture (sites 16–23): 16. Koskurgan and Shoktas in southern Kazakhstan^59^; 17. Bailong^61^; 18. Shuangshu^62^, 19. Yiyuan^63^, 20. Zhoukoudian^64^, 21. Jijiazhuang^17^, 22. Hougou^16^, 23. Bose Basin^65^. Note that Bailong is a site in the Luonan Basin, while Jijiazhuang and Hougou are sites within the Nihewan Basin.

Early humans first dispersed out of Africa approximately 2.6–2.0 million years ago (Ma)^1–6^. The ensuing tempo of eastward migration was rapid (Fig. 1), as the presence of lithic tools shows that early humans had reached the Chinese Loess Plateau by 2.1 Ma^7^. Human fossil discoveries reveal that *Homo erectus* was present in the Caucasus Mountains by 1.8 Ma^8^, in northern China^9^ and southern China^10^ around 1.8–1.7 Ma, and in Java by ~1.5–1.3 Ma^11^. Many archaeological sites have documented the subsequent presence of early humans in large parts of mid- to low-latitude Eurasia by ~1 Ma, including Siberia^12,13^, showing that early humans were capable of exploiting habitats ranging from tropical forests and temperate grasslands to cold-steppe environments. Both southern and northern China host a range of well-dated Early Palaeolithic sites that pre-date 0.4 Ma, including several sites in the Bose Basin^14^, the Luonan Basin^15^, and the Nihewan Basin^16–18^ (Fig. 1). In contrast, to date there have been no well-dated Early Palaeolithic sites pre-dating 0.4 Ma in Central Asia, partly because archaeological sites are relatively scarce in this semi-arid region and none of the existing sites have robust absolute chronologies. It therefore remains unclear how Central Asia fits into the overall picture of early human dispersal patterns in Eurasia. It is possible that Central Asia served as an important migration corridor that linked the southern Caucasus with Northeast Asia^19–21^, including Siberia and northwestern China (Fig. 1). Alternatively, it is also possible that parts of Central Asia were populated from the east via western China^22^. Robust age constraints at sites with typologically diagnostic artefacts on these pathways are needed to advance our understanding of the migration history of Eurasia.

**Fig. 2:**
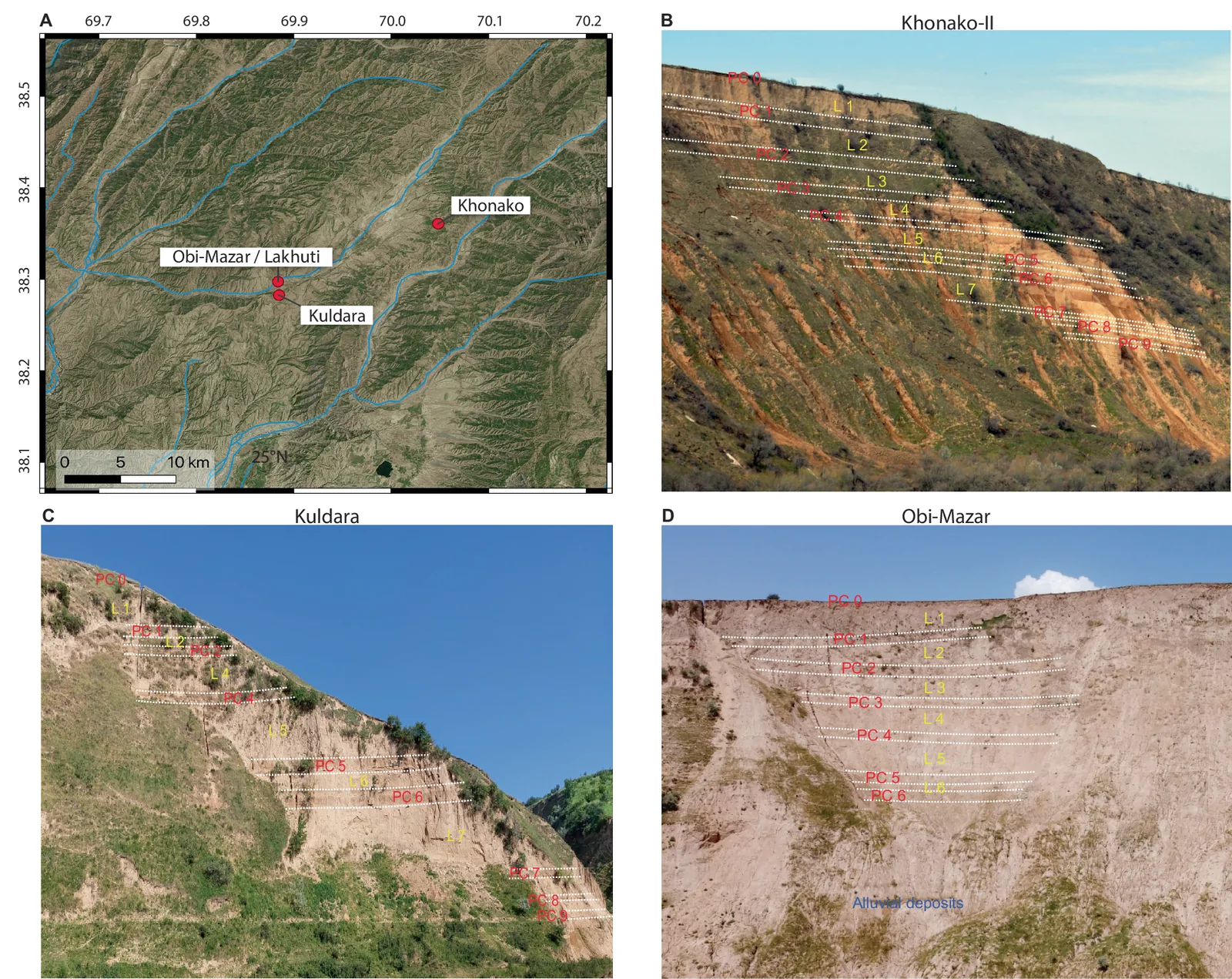
Location and photos of the studied loess profiles. **A** Location of the archaeological sites on the Khovaling Loess Plateau in Southern Tajikistan used in this study, including photographs of **B** Khonako-II, **C** Kuldara, and **D** Obi-Mazar, L: Loess, PC: Pedocomplex. Both Kuldara and Obi-Mazar have surface elevations around 1300 m, whereas the surface elevation at Khonako-II is 1880 m (Table S2). The surface of the sections at Obi-Mazar and Khonako-II is sub-horizontal, whereas the surface is sloping at Kuldara because it is cut by a gulley.

Despite problems with dating, there is ample evidence to suggest that early humans were present in Central Asia during the Early Palaeolithic. The most compelling of this evidence derives from the Khovaling Loess Plateau (KLP) in southeastern Tajikistan, which contains thick ( ~ 200 m) sections of windblown loess (units designated as L) intercalated with palaeosols known as pedocomplexes (PCs; Fig. 2;^23^). Some of these palaeosols host numerous lithic tools, and these loess-palaeosol sections have therefore given rise to the term ‘Loessic Palaeolithic’^24,25^. It is generally believed that the less-weathered loess units were deposited during cold and dry glacial periods and that palaeosols were formed during warmer and wetter conditions associated with interglacial periods—a notion that was recently confirmed for the upper ~250 ka of the sections at Kuldara and Khonako-II using high-resolution luminescence dating^26,27^.

Several of the pedocomplexes (PCs) in the Khovaling Loess Plateau host rich collections of lithic tools that were initially identified and excavated in the 1970s^28–30^. The lithic tools found in the two uppermost pedocomplexes (PC1 and PC2) are comprised of blade industries, that include laminar technology for producing blades and Levallois technology for flakes and points^31,32^. This technocomplex is distinctly different from the more expediently produced lithic tools found in some of the deeper pedocomplexes of the Khovaling Loess Plateau; these archaeological artefacts provide evidence that early humans were present in Central Asia long before the emergence of *H. sapiens* in Africa. The oldest trace of early humans in Central Asia comes from PC12 and PC11 at Kuldara, one of the large vertical sections exposed in the Khovaling Loess Plateau^33^. Palaeomagnetic data of Penkov and Gamov^34^ show that PC12 and PC11 were deposited during the late Matuyama chron—between the Matuyama-Brunhes geomagnetic polarity reversal (around 0.773 Ma;^35^) and the Jaramillo event (1.07–0.99 Ma;^35^)—giving them an approximate age of 960–900 ka. However, the Kuldara assemblages (PC12 and PC11) only contain a few simple flakes, and the absence of typo-technologically diagnostic lithic tools limits what can be said about the early humans that occupied the region at this time.

By far the richest assemblages of lithic tools in the Khovaling Loess Plateau are found in pedocomplexes PC4, PC5, and PC6, which together contain around 6500 artefacts. Soviet pioneer archaeologist Vladimir Ranov^24^ named these assemblages the ‘Karatau Culture’ and characterised the culture as a pebble-core technocomplex, which includes choppers, side-scrapers, and denticulated tools. The technology is characterised by unidirectional parallel or slice reduction techniques with the removal of one or two elements. Later technologies include radial reduction techniques^36^. The culture is further characterised by the absence of prepared cores and a paucity of standardization in tool manufacture^31,36^. According to Ranov^31^, there are indications of technological development from PC5 and PC6, where the lithic tools display characteristics typical of the Early Palaeolithic, to PC4 where the stone tools exhibit some characteristics associated with the Middle Palaeolithic. However, Ranov^31^ only described site-specific assemblages and did not provide a comprehensive definition of the Karatau Culture.

The age of the Karatau Culture has up to now remained uncertain, because none of the units hosting this culture (PC4–6) have been dated using absolute chronological methods. The Karatau Culture is so far constrained in time by a younger limit of ~250 ka^26^, provided by the luminescence ages associated with PC2 (and L3), and an older limit of ~770 ka is provided by the Matuyama-Brunhes boundary^34,37,38^. Stratigraphic correlation of the loess-palaeosol sequences with Marine Isotope Stages (MIS) has suggested that PC4, PC5, and PC6 correspond to MIS 11, 13, and 15, respectively. However, high-resolution luminescence dating of the upper ~250 ka indicates the presence of significant hiatuses that coincide with erosional boundaries^26^; at Kuldara, both L3 and PC3 are missing entirely, implying that dating via stratigraphic correlation cannot be assumed to be reliable in this setting.

The stratified loess-palaeosol sections exposed along rivers cut into the Khovaling Loess Plateau represent important archaeological sites because they provide a record of human activities along with climatic and environmental changes in Central Asia across multiple glacial-interglacial cycles. Up until now, however, none of the layers that host the Early Palaeolithic Karatau Culture or the Kuldara assemblages have been anchored in time by absolute age constraints. Here, we provide a comprehensive description of the Karatau Culture based on close inspection of 3534 artefacts discovered during historical excavations and our recent excavations (Supplementary Tables S6–8). We also provide an exceptionally detailed absolute chronology for three sections from the Khovaling Loess Plateau (Obi-Mazar, Kuldara, and Khonako-II), based on 286 luminescence ages from loess and soil samples, 16 ^26^Al-^10^Be cosmogenic nuclide concentration pairs from quartz-bearing lithics, and palaeomagnetic polarity records based on loess and soil samples (see Supplementary Notes 1–3 in Supplementary Mat.). The luminescence ages, cosmogenic nuclide concentrations, and magnetostratigraphic tie-points are integrated in a combined probabilistic inverse age-depth model for each section, and the age-depth model for each section is subsequently augmented via transposition of age constraints among the sections using automated probabilistic correlation of the associated magnetic susceptibility records. These age-depth models provide an absolute chronological framework for the Early Palaeolithic in Central Asia and temporally constrain ecological and evolutionary characteristics of early human occupations in the region.

## Results

### Description of the Karatau culture

**Fig. 3:**
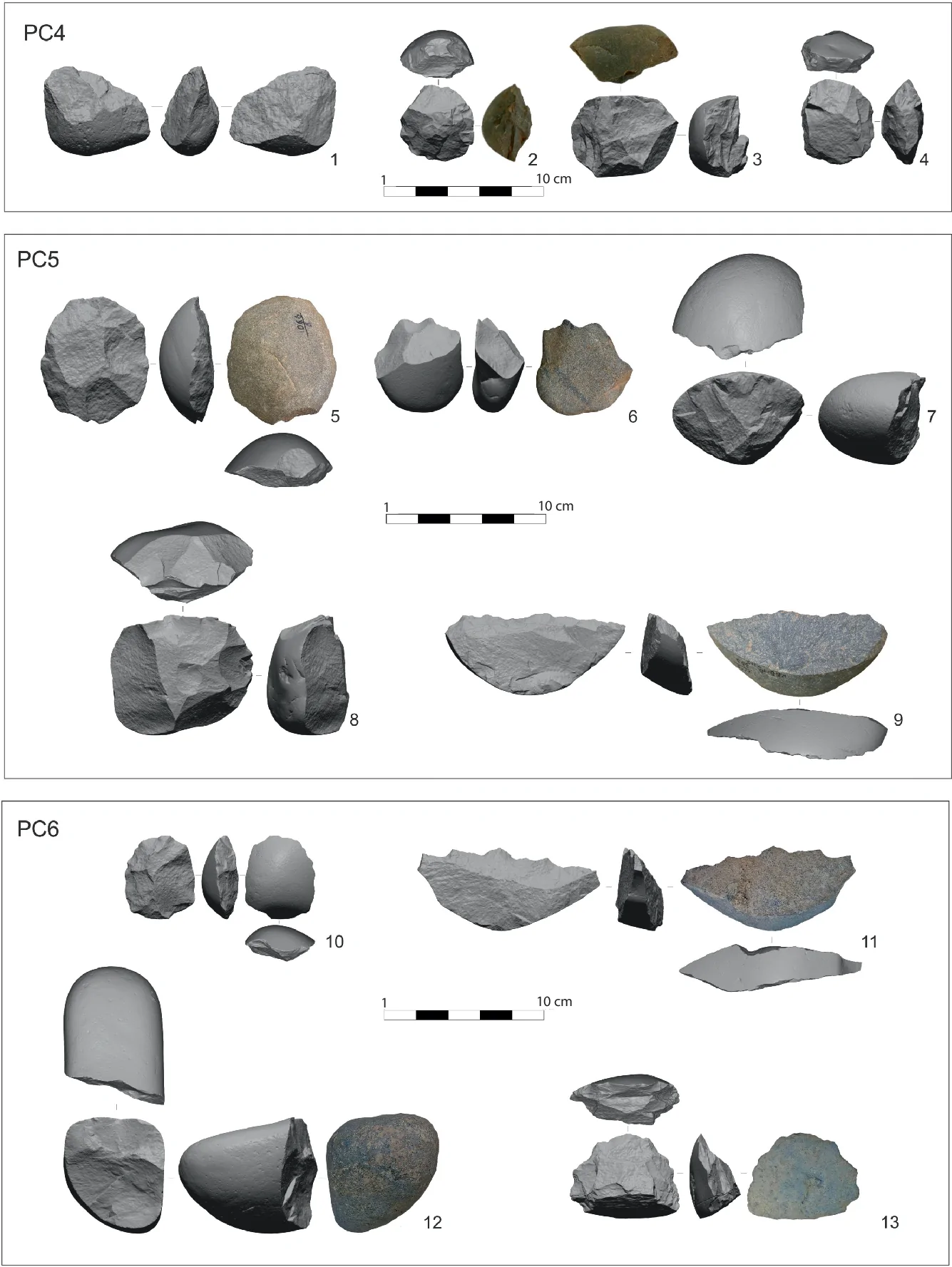
Pictures and 3D models of different types of lithic artefacts of the Karatau Culture. Lithic artefacts found at Lakhuti-IV: 1–4; Lakhuti-I: 5-9; Karatau: 10–13. Types of lithic artefacts: transverse end-scrapers: 1, 9, 11; unifaces: 2, 5, 10, 13; orthogonal parallel core: 3; radial core: 4; chopper: 6; “citron” cores: 7, 12; unidirectional parallel core: 8. PC: pedocomplex.

The Karatau Culture is defined by the rich collection of lithic artefacts found in pedocomplexes PCs4–6. These three distinct pedocomplexes (PCs4–6) have been identified in several different loess-palaeosol sections exposed in the Khovaling Loess Plateau, including Obi-Mazar, Lakhuti, Khonako, Karatau, and Karamaydan^31^. Our analysis of 3534 artefacts shows that the lithic assemblages from the three pedocomplexes PCs4–6 display a high degree of similarity and exhibit minimal typological differences, despite significant differences in the concentration of artefacts (the lowest concentration is found in PC6 and the highest in PC5; this difference does not appear to be related to the availability of raw material). Cores and core-like pebbles account for 4–14% of the assemblages. Several flat-faced techniques have been used in the primary flaking, including the radial and citron techniques (Fig. 3: 3,4,7,8,12). The flat-faced technique is characterised by unidirectional removals only. There are many ‘citron’ flakes (up to 8%), i.e. lemon shaped wedges produced during pebble flaking (see Supplementary Note 5) and the striking platforms are generally flat or cortical. The tool kit, which constitutes up to 5% of the assemblages, contains a significant proportion of retouched flakes. The most distinctive tools include choppers (Fig. 3: 6; 4-G–I: 5,9,16,17), simple scrapers (Fig. 3: 1,9,11; 4-G–I: 10), denticulated and notched tools as well as unifaces (Fig. 3: 2,5,10,13; 4-G–I: 2–4,7,8,14,15), but hammerstones, atypical end-scrapers, and perforators are also present in the assemblages. Waste products, which include small flakes ( < 1.5 cm) and chips indicative of blanks retouching, account for the majority of debitage. No blades have been found in pedocomplexes PCs4–6.

Although the industries found in pedocomplexes PCs4–6 generally exhibit similar characteristics without systematic typological differences, there are some notable variations among the pedocomplexes and among the different sections. First, the relative proportion of cores and various tool types differs among the assemblages. Second, the assemblages from PC6 and PC5 contain examples of some rare tool types that are not observed in PC4. This includes scrapers with intensive retouch (Karatau and Obi-Mazar, PC6), Tayacian retouch points (Lakhuti-IV) in PC6, and scrapers displaying perimeter retouch in PC5 (Lakhuti-I). There are also some systematic differences among the different sites, which is most pronounced in PC4. The PC4 assemblage at Khonako (only found at Khonako-III), located several 100 m above and ~3 km from the Obi-Mazar River, is characterised by a small percentage of cores (3.5% versus 10.2% at Obi-Mazar PC4), a predominance of small flakes (24.3 % compared to 14.6% at Obi-Mazar PC4), and a higher proportion of tools (5.2% versus 0.3% at Obi-Mazar PC4) compared to the PC4 assemblages found at Obi-Mazar and Lakhuti, which are located on the bank of the Obi-Mazar River where raw material for tool making is abundant in the form of river cobbles.

### Age-depth models for the Khovaling Loess Plateau

**Fig. 4:**
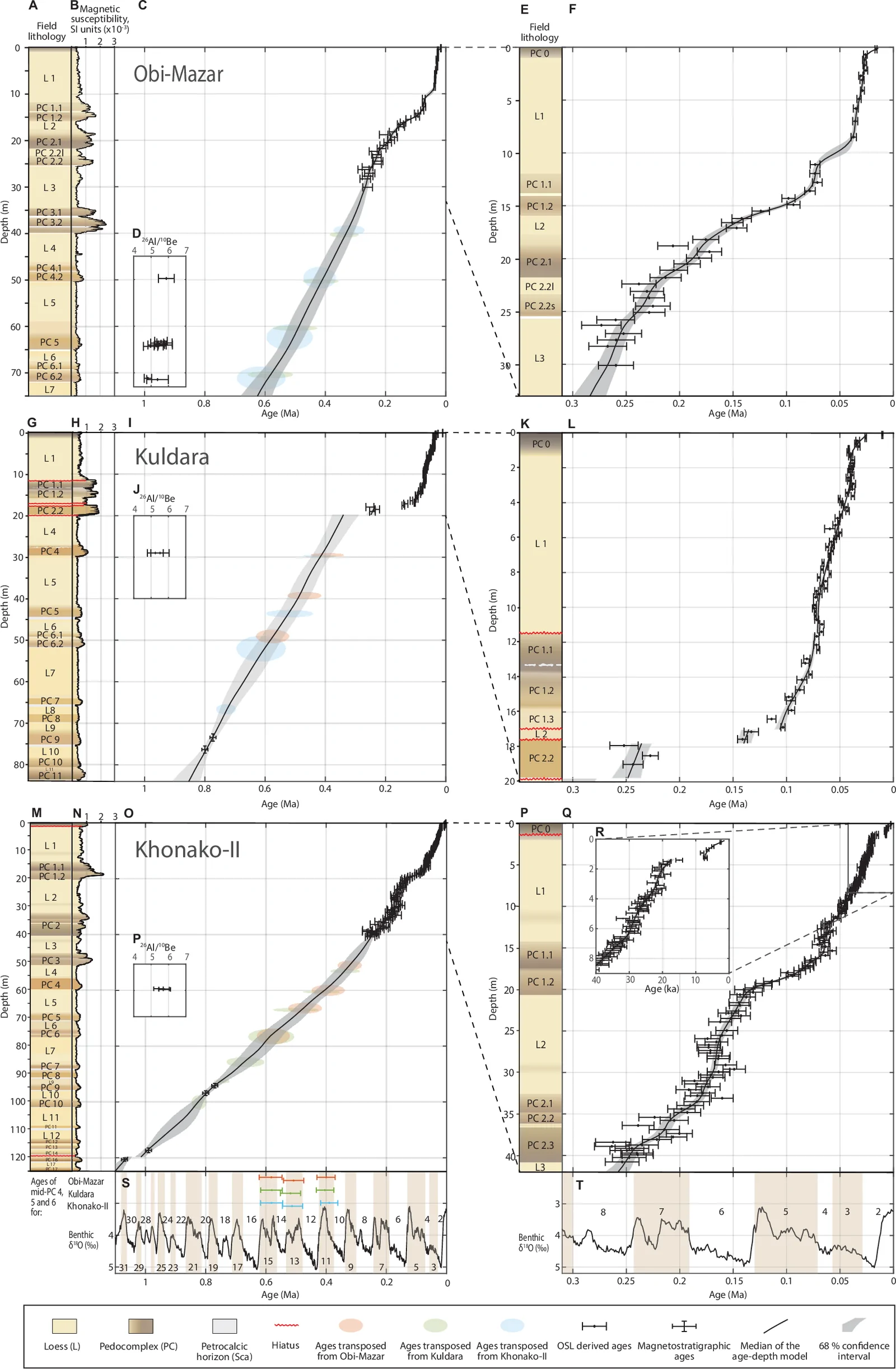
Absolute chronology of the Khovaling Loess Plateau in southern Tajikistan obtained from the Obi-Mazar, Kuldara, and Khonako-II sites. Panels **A**, **E**, **G**, **K**, **M** and **P**: field lithology for each respective site. Panels **B** (*n* = 2317), **H** (*n* = 2861), and **N** (*n* = 1038): magnetic susceptibility profiles for Obi-Mazar, Kuldara, and Khonako-II, respectively. Panels **C** (*n* = 48), **F** (*n* = 40), **I** (*n* = 86), **L** (*n* = 78), **O** (*n* = 181), **Q** (*n* = 168), and **R** (*n* = 68): CosmoChron age-depth models (black lines and grey areas represent the median and 68 % confidence interval, respectively), based on luminescence ages, cosmogenic ^26^Al/^10^Be concentrations, magnetostratigraphic boundaries, and probabilistic alignment of the magnetic susceptibility records (see Fig. S4). Black dots and horizontal error bars represent individual luminescence ages or magnetostratigraphic boundaries, and their corresponding 1σ age uncertainties, respectively. Depth uncertainties associated with the magnetostratigraphic boundaries (vertical black error bars) are shown as the standard deviation of the posterior lock-in depths (see Fig. S9). Coloured ellipses represent transposed ages from the individual age-depth models of Obi-Mazar (red), Kuldara (green) and Khonako-II (blue), with width indicating the 1σ age uncertainty (see Figs. S3–5) and height indicating the 1σ depth uncertainty (see Figs. S6–S8). Panels **D** (*n* = 11), **J** (*n* = 2), and **P** (*n* = 3): measured ^26^Al/^10^Be ratios and the associated 1σ uncertainties based on lithics from PCs 4–6 collected during multiple expeditions between 1976 to 2021 (Supplementary Data 4). The y-axis shows the depth, while the x-axis shows the ^26^Al/^10^Be ratios which decrease with depth reflecting increasing burial age with depth. These ratios are incorporated into the age-depth models by calculating the ^26^Al/^10^Be ratios as a function of the exposure burial history as detailed in the Supplementary Note 4 and Sørensen et al.^74^. Panels **S** and **T** the LR04 benthic δ^18^O stack^50^, with numbered and shaded areas indicating Marine Isotope Stages (MIS). Coloured dots with corresponding error bars represent the median ages and associated 68 % confidence intervals for the mid-depths of the Karatau Culture layers PC4–6, derived from the age-depth models for Obi-Mazar (red), Kuldara (green), and Khonako-II (blue).

The age-depth models associated with the Obi-Mazar, Kuldara, and Khonako-II profiles are presented in Fig. 4 along with the lithology (Fig. 4A, G, M), magnetic susceptibility (Fig. 4B, H, N), and the data that constrain the age-depth models (Fig. 4C, I, O). The upper ~250 ka of the loess-palaeosol sections, which are temporally constrained by luminescence ages (Fig. 4F, L, Q), indicate variable dust accumulation rates across time and space. The reconstructed accumulation rates at Khonako-II show that loess units L1 and L2 accumulated fast with an average rate of 0.350 ± 0.051 m/ka. On the other hand, the accumulation rate was more attenuated during deposition of sediments comprising pedocomplexes PC1 and PC2, in particular during deposition of PC1 sediments (0.078 ± 0.012 m/ka), which on average accumulated ~4.5 times slower than L1 and L2. However, the results also show that ~3 m of sediment accumulated relatively rapidly across the PC1-L1 boundary. The accumulation rates reconstructed for Obi-Mazar show similar trends, suggesting that PC1 sediments deposited at lower rate (0.058 ± 0.015 m/ka) than the non-pedogenically modified loess units (the PC units consist of pedogenically modified loess that accumulated during warmer and wetter periods), and that ~3 m of rapid deposition occurred across the PC1-L1 boundary, comparable to that observed at Khonako-II. However, the systematic difference in accumulation rate between pedocomplex units and loess units is less pronounced at Obi-Mazar compared to Khonako-II, and the general accumulation rate is also slower at Obi-Mazar. The pattern is somewhat different at Kuldara, where the accumulation rate is relatively consistent across L1 and PC1 units. Kuldara also differs from the two other sections in another sense as the luminescence ages reveal the presence of significant hiatuses lasting up to 94 ± 13 ka (at 17.8 m; Fig. S8) The luminescence-constrained age-depth models also show that PC3 is missing entirely at Kuldara, whereas there are no significant hiatuses in the other two sections. The only exceptions include a small hiatus at the very top of Khonako-II lasting 10 ± 1 ka (at 1.35 m; Fig. S8), and one within the Jaramillo subchron at the bottom of Khonako-II lasting 42 ± 17 ka (at 119.7 m; Fig. S8).

The upper ~250 ka (Fig. 4F, L, Q) of the profiles datable by luminescence provide a high-resolution view of the accumulation of sediments during the Pleistocene, but modelled age reconstructions from the parts older than ~250 ka (Fig. 4C, I, O) indicate the deep antiquity of loess deposition in the region. The age-depth models show that sediments comprising the Middle Palaeolithic pedocomplexes, PC1 and PC2, accumulated at ~70–130 ka and ~170–250 ka, respectively (Figs. 4F, Q). PC3, which contains virtually no lithic tools, comprises sediments deposited around 330 ± 35 ka, whereas sediments of pedocomplexes PC4, PC5, and PC6 associated with the Early Palaeolithic Karatau Culture were deposited around ~400, ~500 ka, and ~580 ka, respectively, with a modelled age uncertainty (1σ) of ~35 ka (see Table S4). Sediments comprising pedocomplexes PC7, PC8, and PC9, which host a very limited number of lithic tools (7 in total; all from Khonako-II), were deposited at ~700, ~730, and ~780 ka, respectively. Finally, the pedocomplexes associated with the Kuldara assemblages (PC11 and PC12), which are located below the Matuyama-Brunhes Boundary, have a modelled depositional age of ~930 ka. The age uncertainty (1σ) of the age-depth models for stratigraphic units located below the luminescence age range is generally considerably larger (30–50 ka, Table S4) than for units located within the luminescence age range (upper ~250 ka) which shows 1σ uncertainties of 1–20 ka (Fig. 4). With the available data, it is therefore not possible to reconstruct differences in sediment accumulation rate between pedocomplexes and loess units below the luminescence age range.

## Discussion

### Reliability and precision of the age-depth models

The vertical coupling of luminescence ages, cosmogenic ^26^Al-^10^Be concentrations, and palaeomagnetic boundaries significantly enhances the precision of the age-depth models for the three loess sections at Obi-Mazar, Kuldara, and Khonako. This is particularly evident for the age associated with the Early Palaeolithic Karatau Culture (PCs4–6), as the ages obtained with the simple conventional ^26^Al-^10^Be cosmogenic-nuclide burial method, which treats all samples individually, yield ages with an average uncertainty (1σ) of 146 ka for PCs4–6 (Fig. S3). Even without the augmented age-depth models, which incorporate ages transposed from the two other sections, the age uncertainties of PCs4–6 drop to ~50 ka for the pedocomplexes for which ^26^Al-^10^Be data are available (the relative age error of PC5 drops from 29 to 10 %; Fig. S3, Table S4). For all three sections, the independent age models show that PCs4–6 most likely formed during MIS 11, 13, and 15, respectively. Importantly, this also implies an absence of major hiatuses and missing pedocomplexes (apart from L3 and PC3 at Kuldara); a necessary condition for incorporating transposed ages, as the Probabilistic Dynamic Time Warping (P-DTW)^39^ alignment model potentially could overlook hiatuses.

Inclusion of transposed ages in the augmented age-depth models further refines the age-depth relationships, reducing the uncertainty on the age of PCs4–6 to ~35 ka across all sites (the relative age error of PC5 drops from 10 to 7%). This improvement highlights the benefits of shared chronological constraints, where data from one section augment the age model associated with other sections. Specifically, the ages associated with PC5 and PC6 are primarily constrained by ^26^Al-^10^Be data from Obi-Mazar, while the age of PC4 relies on data from all three sections.

**Fig. 5:**
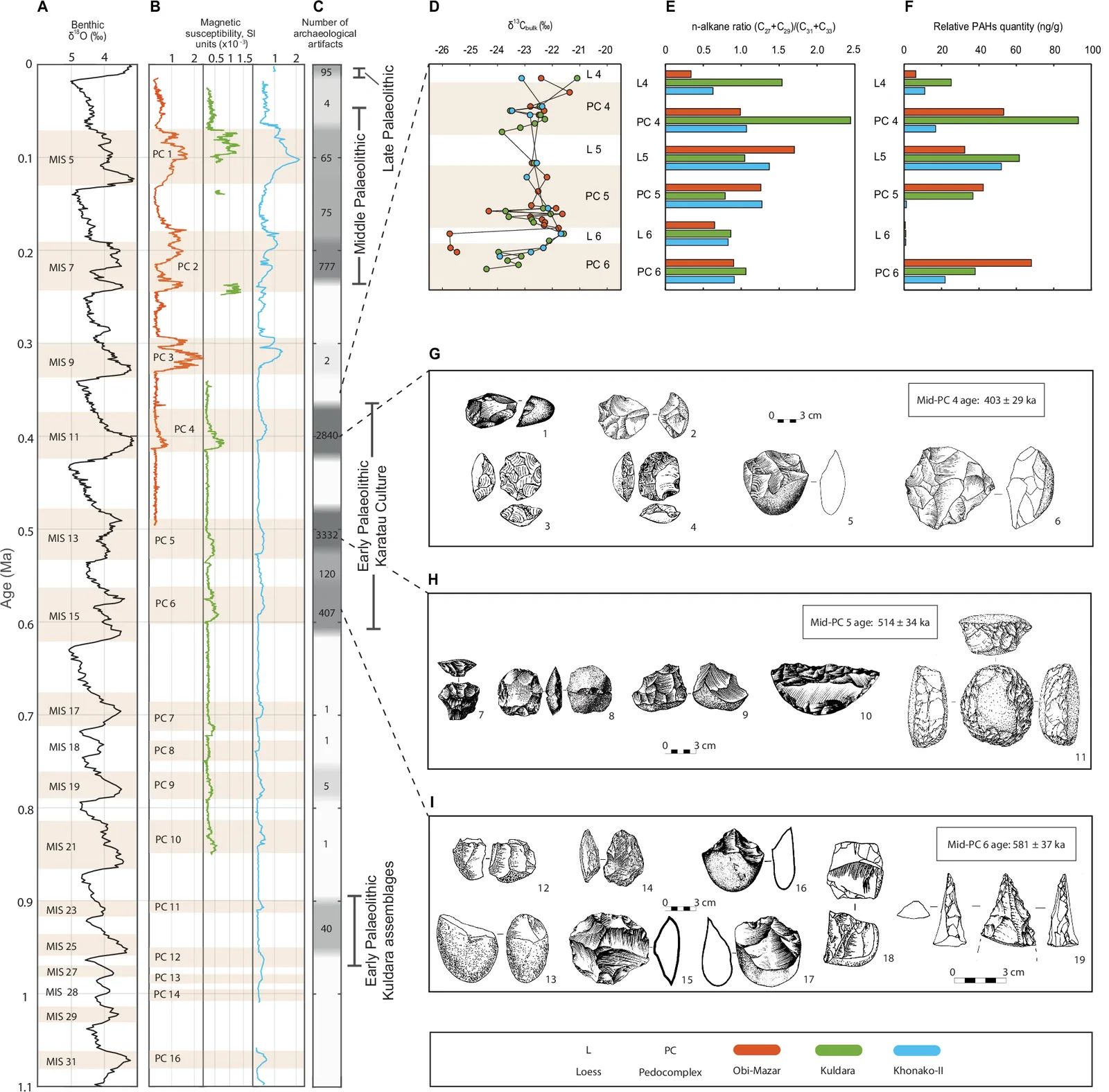
Summary of the findings across the Obi-Mazar (red), Kuldara (green), and Khonako-II (blue) study sites. **A** LR04 δ^18^O stack^50^, **B** Magnetic susceptibility as a function of time for Obi-Mazar (*n* = 2317), Kuldara (*n* = 2861), and Khonako-II (*n* = 1038). The chronology used in the panel represents the median of the age-depth models shown in Fig. 4. Orange shading in panels (**A** and **B**) indicates pedocomplexes (PCs), which differ between the three sites and therefore do not represent uniform or definitive ages of these units. **C** Total number of lithic artefacts found in the Khovaling Loess Plateau based on^82,83^. **D** Stable carbon (C) isotope (δ^13^C vs. VPDB; *n* = 54) values of bulk sediment samples collected from stratigraphic units of study sites. The lines connecting δ^13^C values across units provide insights into vegetation changes over time. More negative δ^13^C values typically reflect dominance of C_3_ and/or closed vegetation under cooler and/or wetter conditions, while less negative values suggest increased C_4_ input and/or open or drier environments. **E** *n*-Alkane ratio (C_27_ + C_29_/C_31_ + C_33_; *n* = 32), used to infer the type of terrestrial plant input. Higher values of the *n*-alkane ratio generally reflect a stronger input from woody vegetation (shrubs and trees), whereas lower values indicate a greater contribution from grasses or herbaceous sources. **F** Relative quantity (ng/g) of polycyclic aromatic hydrocarbons (PAHs) shown by stratigraphic units (*n* = 32). Higher concentrations of PAHs may indicate increased fire activity resulting from either anthropogenic disturbances or natural processes. **G** Lithic artefacts found in PC4. **H** Lithic artefacts found in PC5. **I** Lithic artefacts found in PC6. Lithic artefacts found at Obi-Mazar: 1, 2, 12, 16; Khonako-III: 3–6; Lakhuti-I: 7–11; Karatau: 13, 14, 16, 17; and Lakhuti-IV: 19. Types of lithic artefacts: radial cores: 1, 6; unifaces: 2–4, 7, 8, 14, 15; choppers: 5, 9, 16, 17; transverse side-scraper: 10; circular side-scraper: 11; single parallel cores: 12, 18; citron core: 13; Тауасian point: 19. The artefacts in panels G, H, and I have been reproduced, with permission, from^84^: 1;^69^: 2, 5, 6;^83^: 3, 4;^70^: 7, 8, 10;^71^: 9;^85^: 12, 13. Lithic artefact 11 in panel (**H**) is an original drawing.

With this level of precision, we can now confirm with high confidence that pedocomplexes PCs4–6, which are associated with the Early Palaeolithic Karatau Culture, formed during the interglacial periods corresponding to MIS 11, 13, and 15, respectively, and that PC3 was formed during MIS 9. Additionally, the luminescence ages from Obi-Mazar (*n* = 40) and Khonako-II (*n* = 145) generated in this study support the notion that pedocomplexes PC1 and PC2 were formed during MIS 5 and MIS 7, respectively, which is consistent with the luminescence ages from Khonako-III^40^. The ages associated with the older pedocomplexes (PCs7–9) are consistent with the ages of interglacial periods MIS 17–19 (Fig. 5). Notably, PC8 correlates with an even-numbered MIS, hereby breaking the otherwise apparent correspondence between pedocomplex development and odd-numbered stages. Even-numbered stages are generally classified as cold periods, but MIS 18 is different as it contains a warm interval in the middle of the glacial period (as shown by the marine δ^18^O record). This observation underscores the importance of independent absolute chronologies for loess profiles such as those from the KLP. Furthermore, the ages of PC11 and PC12, associated with the Kuldara assemblages, most likely correspond to MIS 23 and MIS 25 ( ~ 915 and ~970 ka, respectively; Fig. 5), thereby representing the earliest, dated evidence of hominin occupation in Central Asia.

Overall, the integration of multiple dating constraints and shared data across sections yield an absolute and coherent chronology for the Early Palaeolithic Karatau Culture on the Khovaling Loess Plateau. We find that the upper nine pedocomplexes fall within the Brunhes Chron and that their ages are consistent among the three loess profiles. This study thus provides a quantitative and reliable chronological framework for the Early Palaeolithic in Central Asia–a framework that largely supports previous hypothesised correlation-based chronologies for the region^41–45^.

### Presence of early humans in the Khovaling Loess Plateau area through time

The strong similarity among the lithic assemblages associated with pedocomplexes PCs4–6 suggests that they represent one coherent archaeological culture, i.e., the Karatau Culture (Fig. 5). Close inspection of 3534 artefacts does not reveal any substantial differences among the assemblages associated with the three pedocomplexes (PCs4–6) (Tables S6–8). This contrasts with the hypothesis put forward by Ranov^31^, who noted a difference between the lithic assemblages in PC4 and those in PC6 and PC5, which he interpreted as an evolutionary trend from tools typically associated with the Early Palaeolithic to tools indicative of the Middle Palaeolithic (PC4). We do not observe such a trend, and we therefore conclude that the Karatau Culture remained relatively stable throughout PC6, PC5, and PC4. The age-depth models and number of artefacts show that the Karatau Culture was thriving in the region around the Khovaling Loess Plateau in Central Asia during MIS 15, 13, and 11, i.e. over a period of 200–250 kyr (Fig. 5).

In this loess environment, clasts larger than 1 mm could not have reached the study sites without manuporting. However, some loess (e.g. L4 and L5) and palaeosol (e.g. PC3 and PC7) units in the KLP contain small numbers of artefacts, and it might be argued that these could have arisen from vertical mixing by hominins and other animals from over- and underlying artefact-rich units. However, the pronounced stratification of artefacts in, e.g., PC4 and PC6, where layers of tools are separated by only a few cm of sterile soil, strongly suggests that even at times when bioturbation might be expected to be most active (i.e., soil formation with intermittent but significant hominin presence), stratification is preserved over depths of only a few cm. It is consequently difficult to attribute the small tool concentrations found in e.g. PC3 and PCs7–9 to vertical mixing, especially given the low densities in the bracketing loess layers. It is even less likely that at times when soil formation processes were weaker, and evidence for hominin presence is small or negligible (e.g. during loess deposition in MIS 12), that the low tool concentrations can be attributed to vertical mixing. The observed artefact densities therefore likely reflect a more limited hominin presence and tool manufacture/use in the locations we have identified to this point.

With this in mind, we infer that the emergence of the Karatau Culture during MIS 15 was marked by the sudden appearance of lithic tools that ended with the termination of MIS 11. The intervening loess units, which correspond to glacial periods MIS 14 and MIS 12, host a smaller number of artefacts, especially MIS 12, where a near-absence of artefacts indicates that the area was largely depopulated during these glacial periods. Similarly, PCs7–9 and PC3 are also largely without material proof for human presence (10 specimens in total). In combination, the available evidence suggests that the area was largely devoid of human activity during interglacial periods preceding the Karatau Culture (MIS 17, 19, and 21), and that the archaeological tradition disappeared altogether with the onset of the glacial period associated with MIS 10. The lithic industry found in PC2 does not represent a continuation of the Karatau Culture, because the assemblage in PC2 is dominated by blades, and thus typologically as well as technologically distinct from the assemblages in PCs4–6. The lithic culture in PC2 therefore signals the arrival of a new archaeological tradition, and possibly hominin population, during MIS 7. Nevertheless, the sporadic presence of artefacts in loess units L6 and L5 as well as pedocomplexes PC7, PC8, and PC9, possibly suggest that small groups of hominins migrated through the area, both during the intervening glacial periods and the interglacials preceding the Karatau Culture. However, such a sporadic and migratory presence is in stark contrast to the thriving Karatau Culture associated with MIS 15, 13, and 11.

### Climatic and environmental changes associated with the Karatau Culture

Alternations of Pleistocene loess and palaeosol units in Tajik loess sequences are generally interpreted to indicate shifts between cool, dry, dusty, steppe-dominated glacial periods and warmer, wetter, forested interglacial periods^43,46^. Such fundamental ecosystem fluctuations are independently corroborated by bulk stable isotope (δ^13^C, δ^15^N) and *n*-alkane data from KLP sites, indicating the presence of C_3_-dominated woodland taxa during Middle Pleistocene interglacial periods (MIS 15, 13, 11) with more open (grassier) conditions during glacial periods (MIS 14, 10) (Fig. 5D, E^47–49^;). Alternations between different climate-driven habitats may explain why the Karatau Culture thrived during certain interglacial periods and dwindled during glacial periods, but it remains unclear why the Karatau Culture suddenly appeared during MIS 15 and disappeared during MIS 10 and never returned during or after the subsequent interglacial period (MIS 9).

The appearance of the Karatau Culture in PC6 immediately followed MIS 16, the first of the deep glacial phases after the Mid-Pleistocene Transition when the dominant periodicity of glacial-interglacial cycles shifted from 41-kyr to 100-kyr cycles^50^. Our age model confirms that MIS 16 is represented by the deposition of loess unit L7 in the KLP, the first of several anomalously thick loess units deposited in the area (Fig. 4). This association may be a coincidence, but it raises the possibility that a fundamental shift in glacial intensity and associated atmospheric and ecosystem dynamics may have been a factor in the archaeological culture’s initial emergence in MIS 15.

One explanation for the disappearance of hominins may be linked to a possible shift away from a summer precipitation regime to a winter precipitation regime in Tajikistan after MIS 11. At present, precipitation in the Khovaling area is dominated by wintertime westerlies^51^. Magnetic susceptibility records from the KLP show a shift from muted glacial-interglacial variations, recorded in PC4 (MIS 11) and below, to high-amplitude variations recorded in PC3 (MIS 9) and above (Fig. 5^41,52^;), which coincides with the disappearance of the Karatau Culture in Tajikistan. A simple ‘pedogenic-model’ interpretation of this proxy^53^ would suggest wetter interglacial episodes from PC3 (MIS 9) onwards due to an increase in westerly precipitation. An alternative explanation involves a shift from a precipitation regime dominated by weak Indian Summer Monsoon (ISM) rainfall (PC4) to a regime dominated by wintertime westerlies and a weakened ISM^42,52^. Such a shift in seasonality would likely have led to fundamental ecosystem changes, including changing vegetation patterns and numbers and types of large herbivores present on the landscape. It is thus conceivable that changes in seasonality led to unfavourable subsistence conditions for early humans associated with the Karatau Culture during interglacials from PC3 (MIS 9) onwards, potentially explaining the disappearance of the Karatau Culture after PC4 (MIS 11).

### Hominins in central Asia during the early Palaeolithic

The lithic tools represent the only direct source of information on the Karatau Culture, as no bone material has been preserved in the loess-palaeosol sections. However, indirect information may be gleaned from biomarkers indicating that the Karatau inhabitants used fire, perhaps at landscape scales to affect vegetation. The greatest concentration of biomarkers and stable isotopic indicators associated with woodland and fire are found at or in the immediate vicinity of the Lakhuti-IV site, which hosts the highest concentrations of archaeological artefacts among the sites studied here (Fig. 5D, E, F^47,54^;). The occurrence of fire decreases with distance from archaeological sites; off-site (Khonako-II) areas show negligible occurrences of fire, regardless of when the deposits were formed, while sparse on-site (Obi-Mazar) and near-site (Kuldara) areas in the vicinity of Obi-Mazar River have evidence for a high prevalence of fire, suggesting hominin fire-ecology landscape management practices could have existed already at this early time. Hominins are argued to have control of fire at the sites of FxJj20 in Koobi Fora, Kenya, by 1.5 Ma^55^ (Fig. 1), Wonderwerk, South Africa by 1 Ma^56^, and habitual use of fire in European contexts associated with Neanderthals between 400 and 300 ka^57^. Evidence from Lusakert Cave in Armenia also demonstrates Middle Palaeolithic hominin fire use in the southern Caucasus^58^, expanding the geographical range of early fire use during the Early-Middle Palaeolithic. While we cannot argue definitively for ‘habitual’ fire use among the inhabitants of the Khovaling region, we observe patterned differences in distributions of vegetation and fire indicators that appear disconnected from climate or landscape but do appear related to human presence.

The stability and constancy of the lithic industries associated with the Karatau Culture over three consecutive interglacial periods indicate that the bearers of this archaeological tradition arrived at the Khovaling Loess Plateau from somewhere else around the onset of MIS 15 with a fully developed tradition. It is unclear where they came from, however, because none of the Early Palaeolithic Acheulean, or Acheulean-like, assemblages in Central Asia or the Iranian Plateau and Indian subcontinent resemble the Karatau Culture. There are sites in southern Kazakhstan that potentially are contemporary with the Karatau Culture, but the industries at these site complexes differ fundamentally from the Karatau Culture due to their absence of large chopping tools, greater diversity of retouched pointed tools, and use of multiple Levallois methods^59^ (Fig. 1).

In China, there are several Mid-Pleistocene hominin sites that have been constrained to the time span 600–400 ka^16,17,60–64^ (Fig. 1), suggesting this was a critical period for human evolution in East Asia that encompassed the transition from *Homo erectus* to Mid-Pleistocene *Homo* (MPH)^63^. This time span coincides with the Karatau Culture in Central Asia, but the knapping techniques and toolkits associated with most of these Chinese sites are distinctly different from the Karatau Culture. However, there are sites in China, e.g., in the Bose Basin^65^, that have many characteristics in common with the Karatau Culture, including discoidal and radial flaking as well as a high proportion of pebble tools and unifaces^65,66^. This could indicate that the bearers of the Karatau Culture populated Central Asia from the east, and if these regions were monsoonal, would be consistent with the hominins of the Karatau Culture being well adapted for ecosystems of summer precipitation regimes, rather than winter westerly driven precipitation. However, the lithic tool assemblages in China that resemble the Karatau Culture are not well-constrained in time, and they also include bifacial tools^65,66^ which are absent in the Karatau Culture. It is therefore impossible to correlate the Karatau Culture with other contemporary cultures in the region surrounding the KLP. Consequently, our absolute chronology for the KLP sites does not definitively clarify how Central Asia fits into the overall picture of Early Palaeolithic dispersals in Eurasia, but it provides anchor points for the Karatau Culture and Kuldara assemblages for future study. As more sites become reliably dated, our understanding of human migrations over a wide area of Asia will transform, increasingly tied to more robust environmental and archaeological proxies.

## Methods

Permission was obtained from the A. Donish Institute of History, Archaeology, and Ethnography of the Academy of Sciences of the Republic of Tajikistan (Dushanbe, Tajikistan) to collect and export the relevant samples (sample permit No. 34003123-152).

### Archaeological description

During field campaigns in 2021–2023, members of our team excavated PC6, PC5, and PC4 at Lakhuti-IV^36^. Our description of the artefacts and definition of the Karatau Culture are based on all known assemblages associated with PCs4–6, including the artefacts found during our excavations in 2021–2023 and the artefacts excavated by Ranov, which are stored at various scientific departments and museums in Tajikistan. The technological and typological characteristics of the Karatau Culture are illustrated by the best-preserved assemblages, which include the PC6 assemblage from Karatau, the PC5 assemblage from Lakhuti-IV, and the PC4 assemblage from Obi-Mazar PC4. See the Supplementary Note 5 for the full description.

### Luminescence chronology

For each site, detailed luminescence chronologies were constructed based on post-IR IRSL signals from 40 to 63 µm polymineral grains. A SAR pIRIR_200,290_ protocol was used to measure the D_e_ values; this protocol has been successfully applied in other loess contexts (e.g.,^67,68^). Challier et al.^27^ and Buylaert et al.^26^ extensively tested the applicability of this protocol to loess from the Khovaling Loess Plateau. Parts of the luminescence age dataset for Khonako-II and the entire Kuldara data have been presented in Challier et al.^27^ and Buylaert et al.^26^, respectively. Details of the luminescence methodology are presented in the Supplementary Note 1.

### Cosmogenic nuclides

The artefacts used for cosmogenic ^26^Al-^10^Be nuclide analysis were originally collected from multiple sites in Tajikistan (Supplementary Data 4) between 1976 and 2022^36,69–73^. These artefacts were recently made available to us by the National Museum of Tajikistan for the purpose of dating the most prominent loess-palaeosol sections associated with the Khovaling Loess Plateau, including PCs4–6. The quartz separation and geochemical preparation of the samples were carried out at the Cosmogenic Nuclide Laboratory, Aarhus University, and the ^26^Al-^10^Be concentrations were measured at the Aarhus Accelerator Mass Spectrometry (AMS) Centre.

The quartz was isolated through crushing, sieving (250–500 μm), magnetic separation, hydrochloric acid leaching, and hydrofluoric (HF) etching. About 20 g of purified quartz was then digested in HF together with in-house ^9^Be and ^27^Al carriers added to the samples in the laboratory. Beryllium and aluminium were separated via ion exchange chromatography, oxidised, and mixed with Nb and Ag powders, respectively, before the samples were pressed into cathodes for AMS analysis. ^10^Be/^9^Be and ^26^Al/^27^Al ratios were normalised to ICN standards, and procedural blank values were subtracted.

Samples from parallel, neighbouring sections were placed on the same depth scale relative to the palaeosols. This includes stratigraphic units at Obi-Mazar that can be tracked by eye and directly correlated with units at Lakhuti. The same is true for the relationship between Khonako-II and Khonako-III.

### Age-depth modelling

The age-depth models presented in Fig. 4 were constructed using the CosmoChron method^74^, a probabilistic inverse modelling approach that integrates cosmogenic ^26^Al-^10^Be concentrations and/or direct age constraints. First, we produced an age-depth model for each of the three individual sections at Obi-Mazar, Kuldara, and Khonako based on luminescence ages from the upper ~250 ka of the profiles, paired ^26^Al-^10^Be concentrations, and magnetostratigraphic boundaries (Fig. S3). In total, we use 286 luminescence ages, which include 185 previously unpublished ages (Supplementary Data 1–2) and 101 published ages^26,27^ (Supplementary Data 3), and 16 unpublished ^26^Al-^10^Be concentration pairs (Supplementary Data 4) from PC4, PC5, and PC6, and four known magnetostratigraphic boundaries^37,38^ (Table S3). Next, we use the magnetic susceptibility records associated with the three sections to improve the precision of the age-depth models (Fig. S4–7; Supplementary Data 9–11).

The free parameters in the CosmoChron model include the (i) mean, variance, and correlation range of the accumulation rates, (ii) hiatus durations associated with observed unconformities, (iii) pre-burial exposure histories associated with the ^26^Al-^10^Be samples, and (iv) palaeomagnetic lock-in depths. Prior knowledge is used to constrain each of these parameters, including the constraint that the accumulation rate must be positive to ensure that the age increases with depth, as detailed in the Supplementary Note 4.1. The CosmoChron forward model generates age-depth relationships and ^26^Al-^10^Be concentrations at specific depths, which are subsequently compared against empirical observations including luminescence ages, measured ^26^Al-^10^Be concentrations and palaeomagnetic boundaries. This inverse problem is solved using the extended Metropolis algorithm^75^, a Markov Chain Monte Carlo method, which is implemented using the SIPPI toolbox for MATLAB^76^. The age-depth models are defined by the accepted accumulation rates and hiatus durations, and the reported age estimates are calculated as the median along with the 15.9 and 84.1% percentiles, corresponding to the 68% confidence interval. For further details on the CosmoChron method, see the Supplementary Note 4 or Sørensen et al.^74^.

The magnetic susceptibility signals associated with the three sections are used to augment the age-depth models of the individual sections through probabilistic alignment using the Probabilistic Dynamic Time Warping (P-DTW) algorithm^39^. The depth-depth correlation of the magnetic susceptibility records enables transposition of age constraints among the three sites, which adds additional age constraints to the individual sections. The uncertainty associated with the depth-depth alignments, which may have a complex multimodal distribution, defines the prior depth distributions associated with the transposed ages (Fig. S5–7). The added precision of the transposed age constraints therefore depends directly on the uncertainty associated with the depth-depth alignments. The incorporation of transposed ages in the augmented age-depth models leads to lower uncertainties in all three sections (Obi-Mazar, Kuldara, and Khonako-II) for the portions that are older than ~250 ka, because of the scarcity of age constraints below the luminescence depth range and the relatively low uncertainty on the depth-depth correlations among the three sections.

### Reporting summary

Further information on research design is available in the Nature Portfolio Reporting Summary linked to this article.

## Supplementary information

Supplementary Information

Description of Additional Supplementary Files

Supplementary Data 1-3

Supplementary Data 4-8

Supplementary Data 9-11

Supplementary Data 12-14

Supplementary Data 15-17

Supplementary Data 18

Reporting Summary

Transparent Peer Review file

## Data Availability

The luminescence, cosmogenic nuclide, magnetic susceptibility, and biomarker data used to produce Figs. 4 and 5 (including previously published data) are provided in the Supplementary Data and in FigShare (https://doi.org/10.6084/m9.figshare.31249798). Supplementary Data files include the luminescence data (Supplementary Data 1–3), cosmogenic nuclide data (Supplementary Data 4–8), and magnetic susceptibility data (Supplementary Data 9–11). Furthermore, the age-depth models shown in Fig. 4, as well as the biomarkler data presented in Fig. 5, are also included in the supplementary data files (Supplementary Data 12–14 and Supplementary Data 15–17, respectively). The archaeological artefacts analyzed in this work are located at the National Academy of Science of Tajikistan in Dushanbe. A list of all museum accession numbers is provided in Supplementary Data 18. Please contact Tura Khudjageldiev (tura959@mail.ru) concerning access to archaeological samples.

## Code availability

The software used to establish the age-depth models are available on GitHub.com: (CosmoChron: https://github.com/aaaasssskkkkeeee/cosmochron), (P-DTW: https://github.com/aaaasssskkkkeeee/P-DTW), (P-PINI: https://github.com/cosmojesper/ppini), and (SIPPI: https://github.com/cultpenguin/sippi). All versions correspond to those available as of 24 September 2025.
