# Supplementary material for: Absolute chronology of the Early Palaeolithic Karatau Culture in Central Asia
Source: Nat Commun. 2026 Jul 1;17:8217. doi: 10.1038/s41467-026-74938-5 (PMC13462899; doi:10.1038/s41467-026-74938-5)
Supplement: Supplementary file 1 [file 41467_2026_74938_MOESM10_ESM.pdf]

nature portfolio

Peer Review File

Absolute chronology of the Early Paleolithic Karatau Culture in Central Asia

Corresponding Author: Dr Aske Sørensen

This file contains all reviewer reports in order by version, followed by all author rebuttals in order by version.

Version 0:

Reviewer comments:

Reviewer #1

(Remarks to the Author)
This is a useful study that provides improved chronological resolution for an important record of Paleolithic occupation. The chronological framework will be of interest to archaeologists. The methodology appears technically sound and robust. However, I have concerns about how the authors frame the significance of their findings. This study uses new methods to confirm and refine what has been established through decades of previous work on the loess sections of Tajikistan. It is valuable, but is not a major step forward in understanding human evolution and dispersal, as the authors suggest. In particular, the discussion of the findings in the context of hominin migration is poorly presented.

I do not agree that the refined chronology of the loess sections in Tajikistan informs our understanding of the earliest migrations into Eurasia. The argument is poorly developed. Two hypotheses for dispersal routes are briefly summarized in the opening paragraph (one lacking a citation), and a handful of sites are used as examples. These sites span approximately 1.5–2.5 Ma—some 500 ka to 1.5 million years before the artifacts presented in this study. Unless the assemblages presented here date to the same period as these early migrations, it is unclear how they can inform those dispersal routes.

Furthermore, the choice of sites highlighted in the introductory text and Figure 1 is puzzling. Many archaeologists are skeptical of reference 1 (Scardia, G. et al. 2019. “Chronologic constraints on hominin dispersal outside Africa since 2.48 Ma from the Zarqa Valley, Jordan”), yet this site anchors the authors’ opening argument. Other relevant and more widely accepted sites are not discussed at all. As an example, Figure 1 of Curran et al. 2025 (“Hominin presence in Eurasia at least 1.95 million years ago”) includes a detailed map of over 30 potential artifact, fossil, and cut-mark sites in Eurasia older than 1 Ma, which they discuss in the text and acknowledge when sites are controversial (including Scardia et al. 2019). The majority of these sites are not mentioned in this current study or included in Figure 1. I encourage the authors to be more thorough in establishing this contextual background.

I also find Figure 1 confusing and hard to follow. The sites do not correspond chronologically with the direction of the arrows. For example, the proposed migration path flows from Koobi Fora (~1.8 Ma) to the older Zarqa Valley site (2.5 Ma) and then to KLP. I am not sure why these sites would be selected to illustrate the dispersal route or why arrows are positioned where they are. What do the “?”’s represent?

Regarding the age model, while I am not a geologist, I am familiar with cosmogenic nuclide dating and its limitations. I do not have concerns about the authors technical approach and find their methods to be thorough.

In summary, this is a well-executed study. The new age model and artifact analysis confirm and refine many of the prior assumptions that were based on stratigraphic correlation. The results support that the Karatau Culture thrived in the KLP during MIS 15, 13, and 11 and remained relatively stable through PC6-PC4.

The new chronology provides valuable context for understanding the lithic assemblages in the KLP. However, it is less helpful for understanding Pleistocene migrations into Eurasia broadly, especially for periods that predate the assemblage by a million years or more, and, because it was already widely accepted that hominins occupied southern Tajikistan around this time.

Does this new evidence change our understanding of hominin dispersal, or does it provide refined support for the established timeline of hominin occupation in the Tajik depression? I believe it is the latter. The authors should frame the

significance of their findings within a more modest and regionally grounded context. In its current form, the background lacks sufficient detail and there is a misalignment between the solid evidence and an overstated interpretation.

Reviewer #2

(Remarks to the Author)
This manuscript entitled "First absolute chronology of the Early Palaeolithic in Central Asia" presents a novel and robust chronological framework for the Early Palaeolithic Karatau Culture in southern Tajikistan, Central Asia. The high-resolution temporal anchors of these key sites offer crucial insights into the evolution and migration of Mid-Pleistocene hominins across the Asian continent. This study is well-suited for publication in Nature Communications pending moderate revisions.

The paleomagnetic data from source references, which is one of the vital evidences to support the chronological conclusion of this manuscript, have been carefully evaluated. Those rock magnetic and magnetostratigraphic results integrate well with the new luminescence and cosmogenic 26Al-10Be ages presented in the manuscript. Nevertheless, several minor issues require attention and further elaboration:

1. Was the Khovaling Loess Plateau affected by tectonic activity since the Mid-Pleistocene? If so, it should be clarified whether the loess-paleosol strata in this area have experienced tilting.
2. The three loess-paleosol sections are located within the same plateau region. Could the authors provide the elevations of the three sections and confirm whether they all developed on the same river terrace level?
3. Several sedimentary hiatuses identified from Kuldara section (at depth interval of 20–11 m) are not observed in the neighboring Obi-Mazar section. What factors account for the gaps and the differential preservation in sedimentary continuity between these two sections?
4. The lock-in depth model is used in the Supplementary file (section 4.1.4) to explain the offset of the M/B boundary in loess deposits relative to marine records. Why other potential factors, such as magnetic minerals differences or pedogenic process, were excluded from consideration?
5. Considering the close archaeological connections between Central and East Asia, the discussion (lines 418–420) could be strengthened by incorporating recent chronologic advances and inter-regional comparison. In fact, many contemporaneous palaeolithic sites in China were well-constrained by different dating methods during last decade. Here are some literatures that might be helpful.
a) Ao H, Liu C, Roberts A P, et al. An updated age for the Xujiayao hominin from the Nihewan Basin, North China: Implications for Middle Pleistocene human evolution in East Asia. Journal of Human Evolution, 2017, 106: 54–65. DOI: 10.1016/j.jhevol.2017.01.014
b) Yang S, Deng C, Zhu R, et al. The Paleolithic in the Nihewan Basin, China: Evolutionary history of an Early to Late Pleistocene record in Eastern Asia. Evolutionary Anthropology, 2020, 29 (3): 125–142. DOI: 10.1002/evan.21813
c) Ji H, Liu C, Yin G, et al. ESR dating of the Hougou Paleolithic site in the Nihewan Basin, North China, using both additive and regenerative dose methods. Quaternary International, 2022, 619: 72–80. DOI: 10.1016/j.quaint.2021.10.001
d) Michel V, Jourdan F, Moncel M-H, et al. High-precision 40Ar/39Ar dating of Australasian tektites associated with bifacial tools in the Bose Basin (Xiaomei and Fengshudao sites), South China and in Vietnam (Go Da and Roc Tung 1 sites). Quaternary Science Reviews, 2024, 346: 109065. DOI: 10.1016/j.quascirev.2024.109065
e) Ye Z, Pei S, Tu H, et al. 26Al/10Be burial dating and technological strategies of hominins at the Jijiazhuang Paleolithic site, Nihewan Basin, China: Implications for understanding Middle Pleistocene human adaptations in east Asia. Quaternary Science Reviews, 2024, 339: 108837. DOI: 10.1016/j.quascirev.2024.108837

Besides, there are also a few unclear statements/illustrations and typos need to be amended:

6. (Lines 66–71, Figure 1) It would be helpful to include age constraints for the sites in the figure or caption. The current figure caption mislabels Sites 5 and 6. Site 5 should be “Nihewan Basin” in northern China and Site 6 should be “Niujianbao” (not “Niujianbuo” as in the text) site in southwestern China.
7. (Line 135) Should be “magnetostratigraphic”.
8. (Line 241, Figure 5) Magnetic susceptibility curves in column B are overlaid, which impedes clear visualization.
9. (Supplementary file, Line 488) Should be “stratigraphic”.

Reviewer #3

(Remarks to the Author)
Sorensen et al present a detailed geochronological study of a loess-paleosol sequence combined with a detailed archaeological analysis of the lithic artefacts contained in this sequence. The time-span covered by this terrestrial sediment sequence is extraordinary, the geographical position of the site, that is situated in the Khovalin loess plateau in Tajikistan, is critical for understanding human migration pathways out of Africa and into the Asian continent throughout the Pleistocene. The study rests on a geochronological dataset that is exceptionally large in the upper part of the loess-paleosol sequence (ca. 0-300 ka), where it is based on luminescence dating, and CRN dating of lithic artefacts and paleomagnetics on the loess section itself for the lower part of the section (from ca. 400 ka to 1 Ma). An age model is constructed and tested that combines the various numerical approaches and also integrates the cosmogenic production and erosion history of the pebbles that were ultimately used for artefact production.

This review focuses on the georchronological aspects and the overall structure of the manuscript. The geochronological methods are sound and well tested and carefully interpreted in terms of paleo-climate and sedimentation rates and archaeological implications. The story and data presented by Sorensen et al are significant and worthwhile publishing in Nature Communications, however, there are several points that need improvement to enhance the clarity of the manuscript and the logic of arguments, particularly in the main text of the paper. One key issue is the following:
Part of the dataset and key methodological foundations have already been published in Buylaert et al. (2024) and Cheallier et al. (2024). While the supplementary materials for the present manuscript contain the relevant methodological details, the main text currently lacks clarity and cohesion. Specifically: (i) it is often unclear whether statements and results attributed to "Sorensen et al." refer to the authors' new analyses versus previously published work (Buylaert et al., 2024; Cheallier et al., 2024); (ii) logic gaps arise because claims in the main text are not consistently linked to the relevant figures and/or supplementary sections; and (iii) the manuscript assumes substantial prior knowledge of the Khovalin loess plateau stratigraphy and chronology (across multiple sites), which needs to be explicitly articulated to be accessible to readers.
Another aspect if related to the uncertainties that arise from the various dating and modeling exercises that should be stated more clearly, and also stated in relative terms (rel err) where relevant, to make the various age claims, rates etc intercomparable and better understandable.
The numbered remarks below (in manuscript order) address these issues as well as additional methodological and minor points.

Line 81: uppermost part = which PCs/LCs/ age range?
Line 121 + 123: ages of Karatau Culture are your findings? From some other authors? Cite / clarify
Line 133: 16 CRN pairs … provide a minimum of context and explanation – measuredon what? Refer to supplement section and ev. Figs there; provide citation for this dating principle (which is currently rather exotic) here too if appropriate.
Line 153: what is the "citron technique"? pls explain or replace with more common terminology or add citation to an accessible paper the reader can refer to.
L 184: Section age-depth models   refs to supplement non existing!
L 185: what precisely do you mean with "upper parts" – refer to Fig where it can be seen, do you still talk in terms of ref 17 and 18 or your own study?
L 188 and 190 – what is the error on these erosion numbers!?
L 185-204: how does loess accumulate in your opinion during PC formation? is there accumulation that can be resolved within error? Or only pedogenic overprinting or older/pre-existing loess?
L 199: hiatuses – can they been specified? Horizon of non-deposition, erosional hiatus..?
L 205 + 207 : The upper parts … versus lower parts … ? Refer specific to these parts via Fig., or cite…
L 209: (Fig. 4F and 4Q, Challier, 2024)   unclear what are your and Chevallier et al data. (also: et al missing)
L 209: you cite your Fig for the first time starting with F, would be better to introduce A, B etc much earlier. Explain your site, stratigraphy, chronology step by step rather than assuming everybody knows papers 17 and 18 already.
L 220: ..within the luminescence age range ..   what is the uncertainty (for the OSL ages/the mode)?
L 223: Figure 4:
this Fig is key and should be a stand-alone Figure, i.e. needs to be better integrated/explained step-by-step in main text and in Fig caption + reference to supplement for details.
Unclear how transposing of ages and colour coding works (line 231 – transposed ages = which ages?)
The symbol for the magnetostratigraphic ages is very hard to read in the A4 print out version in Fig 4 I and O.
The CRN concentration pairs are important and need a minimum on explanation here already + ref to Suppl. (e.g line 229) – what type of samples? do they come from PC 4, 5 and 6? How do they fold into the model – i.e. do they project also onto the x-axis (age in Ma)?
L 236: ages of Karatau cultural layers derived from this age model? From other archaeological publications (cit)?

L 266: significantly enhances the precision   by what? Specify.
L 270 + 272: here and on several other occasions: give relative error as well to make it intercomparable.
L 277: P-DTW = ?
L 279: the age of PCs 4-6 to 35 ka   provide relative error too
L 287: the new luminescence ages   published versus new data (this study)… n=?
L 293: is too large   add error in %
L 313: Fig. 5   integrate and cite at beginning of section (and whenever required in-between), not at the end;
L 358: MIS 9   but there are 2 artefacts from that section (attributed to bioturbation in the supplement) – what about 3D spatial heterogenous pattern of camp-sites versus 2D outcrop?
L 367 onward: this paragraph seems to link directly with the paragraph L349-358, merge!?; disappearance   during glacials? During MIS 9?
L 382: the disappearance of the archaeological culture after PC4/MIS 11.   but there is archaeology throughout the entire section! Be more clear/explicit about the Karatau culture (which you are refering here to I guess) versus other cultures – show this in Fig 5 more explicitly too.
L 70: site 12 = ?

Supplements:
L 39: ref 7 is about Q OSL, you extract polymin "FG" for IRSL.
L 178: these samples – which ones?
L 179: Samples used for the age-depth model… n=?
L 180: S3 = Fig Table?
L 341: which delays the acquisition of a stable signal – in loess or marine sediments?
L 385: range generally increase   how much in %?

L 393: uncertainties in the upper parts of the profiles increase   how much in %

Version 1:

Reviewer comments:

Reviewer #1

(Remarks to the Author)
Thank you for the opportunity to review this manuscript. I am happy with the revision and I feel the authors have addressed my concerns. I recommend this paper for publication.

Reviewer #2

(Remarks to the Author)
The authors have thoroughly revised their manuscript and provided detailed responses to the reviewers' comments. In my view, the manuscript is significantly improved and is suitable for publication subject to the following minor revisions:

Figure 1 Caption: (1) correct the name of Site 8 to "Niujianbao," as previously noted. (2) Correct the name of Site 18 to "Shuangshu."(3) Both Site 11 and Site 23 are currently labeled as "Bose Basin." Please replace one of them with a more specific site name.

Figure 4: It is suggested to add numerical scales and labels to the horizontal axes in the diagrams of Obi-Mazar and Kuldara sections.

Reviewer #3

(Remarks to the Author)
The authors have addressed the questions and comments and I recommend this overhauled version of the manuscript for publication in Nat.Comm.

Open Access This Peer Review File is licensed under a Creative Commons Attribution 4.0 International License, which permits use, sharing, adaptation, distribution and reproduction in any medium or format, as long as you give appropriate credit to the original author(s) and the source, provide a link to the Creative Commons license, and indicate if changes were made.
In cases where reviewers are anonymous, credit should be given to 'Anonymous Referee' and the source.
The images or other third party material in this Peer Review File are included in the article's Creative Commons license, unless indicated otherwise in a credit line to the material. If material is not included in the article's Creative Commons

license and your intended use is not permitted by statutory regulation or exceeds the permitted use, you will need to obtain permission directly from the copyright holder.
To view a copy of this license, visit https://creativecommons.org/licenses/by/4.0/

Response to Reviewer 1

Reviewer 1 summary of assessment
This is a useful study that provides improved chronological resolution for an important record of Paleolithic occupation. The chronological framework will be of interest to archaeologists. The methodology appears technically sound and robust. However, I have concerns about how the authors frame the significance of their findings. This study uses new methods to confirm and refine what has been established through decades of previous work on the loess sections of Tajikistan. It is valuable, but is not a major step forward in understanding human evolution and dispersal, as the authors suggest. In particular, the discussion of the findings in the context of hominin migration is poorly presented.

Response: We thank the reviewer for the comments and constructive feedback on our paper. We appreciate the comments concerning the framing of the paper and acknowledge that the background introduction was a bit disconnected from our results, which gave our paper a somewhat misleading context. We have addressed this concern by redrafting the first paragraph of the introduction and the last paragraph of the discussion. We have also modified Fig. 1, so that it is now fully aligned with the text in the introduction and discussion. Note that we have decided to keep a brief description of the oldest sites in Eurasia in the introduction, because it provides important background information. This information is particularly important in the context of the ~970 ka old Kuldara assemblages that we also mention and date in our paper. However, we also add more information on Chinese sites that are contemporary with the Karatau Culture – as suggested by reviewer 2. This gives a better and more accurate context for our findings, including the new chronology of the Karatau Culture.

Finally, we acknowledge that the importance of our findings was poorly framed, because our results do not by themselves improve our understanding of early human evolution and dispersal as such. However, we believe that absolute and precise chronologies are crucial to understand early human evolution and migrations patterns across Eurasia. Once more Early Paleolithic sites with robust chronologies become available, we will be able to solve this puzzle. The Khovaling Loess Plateau is important in this context, because there are no Early Paleolithic sites with robust, absolute chronologies in this region, and because the KLP sites preserve a semi-continuous record of human activities and environmental changes over the last ~1 Ma. We have tried to give a more accurate description of this context in the revised paper.

Reviewer 1 Comment 1
I do not agree that the refined chronology of the loess sections in Tajikistan informs our understanding of the earliest migrations into Eurasia. The argument is poorly developed. Two hypotheses for dispersal routes are briefly summarized in the opening paragraph (one lacking a citation), and a handful of sites are used as examples. These sites span approximately 1.5–2.5 Ma—some 500 ka to 1.5 million years before the artifacts presented in this study. Unless the assemblages presented here date to the same period as these early migrations, it is unclear how they can inform those dispersal routes.

Response: We appreciate this comment and acknowledge that the context of our findings was poorly presented. In response, we have reframed the first paragraph of the introduction and the last

paragraph of the discussion to provide a more accurate context. We have also changed Fig. 1 accordingly. That being said, we do believe that robust, absolute chronologies are key for our understanding of early human dispersal patterns – but more sites with robust chronologies are needed. As mentioned above, that is the context we strive to convey in the revised paper.

Reviewer 1 Comment 2
Furthermore, the choice of sites highlighted in the introductory text and Figure 1 is puzzling. Many archaeologists are skeptical of reference 1 (Scardia, G. et al. 2019. "Chronologic constraints on hominin dispersal outside Africa since 2.48 Ma from the Zarqa Valley, Jordan"), yet this site anchors the authors' opening argument. Other relevant and more widely accepted sites are not discussed at all. As an example, Figure 1 of Curran et al. 2025 ("Hominin presence in Eurasia at least 1.95 million years ago") includes a detailed map of over 30 potential artifact, fossil, and cut-mark sites in Eurasia older than 1 Ma, which they discuss in the text and acknowledge when sites are controversial (including Scardia et al. 2019). The majority of these sites are not mentioned in this current study or included in Figure 1. I encourage the authors to be more thorough in establishing this contextual background.

Response: We understand and partly agree with this comment. First of all, it is important for us to say that we never intended to give a full account of all the sites in Eurasia that are older than 1 Ma. We wanted to give readers an impression of how early the first hominins reached different parts of Asia, hereby demonstrating that early humans were present in regions to the west, east, south, and north of the KLP long before the Kuldara assemblages and, in particular, before the Karatau Culture. We also wanted to give a more regional context that focused more on Central Asia but is difficult as we only want to include sites with robust, absolute chronologies.

In the revised paper, we have improved the alignment between Fig. 1 and the text. Also, we only include sites that are mentioned in text, either because they are important for the general background context, the description of fire use, or because they are comtemporary with the Karatau Culture. Finally, we decided to keep Zarqa Valley (Sukhna) because it is in the literature (and also mentioned in Curran et al., 2025), but we group it together with Masol (India) and Longgupo (China) to highlight there is evidence for early human presence outside Africa as early as 2.6-2.0 Ma.

Reviewer 1 Comment 3
I also find Figure 1 confusing and hard to follow. The sites do not correspond chronologically with the direction of the arrows. For example, the proposed migration path flows from Koobi Fora (~1.8 Ma) to the older Zarqa Valley site (2.5 Ma) and then to KLP. I am not sure why these sites would be selected to illustrate the dispersal route or why arrows are positioned where they are. What do the "?"'s represent?

Response: Yes – we acknowledge that several aspects of Fig. 1, including the arrows, were confusing. We have tried to address this in the revised paper. We have decided to keep the arrows, although now in a different style, because they highlight the uncertain role of Central Asia. Did early humans arrive in this region from the west or from the east? We have also decided to keep Koobi Fora, Wonderwerk Cave, and Lusakert Cave in Fig. 1, because we

refer to these sites in the discussion of the early use of fire. We now make that clear in the caption and with the new layout of the figure.

Reviewer 1 Comment 4
Regarding the age model, while I am not a geologist, I am familiar with cosmogenic nuclide dating and its limitations. I do not have concerns about the authors technical approach and find their methods to be thorough.

Response: Thank you for this comment.

Reviewer 1 Comment 5
In summary, this is a well-executed study. The new age model and artifact analysis confirm and refine many of the prior assumptions that were based on stratigraphic correlation. The results support that the Karatau Culture thrived in the KLP during MIS 15, 13, and 11 and remained relatively stable through PC6-PC4.<br><br>The new chronology provides valuable context for understanding the lithic assemblages in the KLP. However, it is less helpful for understanding Pleistocene migrations into Eurasia broadly, especially for periods that predate the assemblage by a million years or more, and, because it was already widely accepted that hominins occupied southern Tajikistan around this time.<br><br>Does this new evidence change our understanding of hominin dispersal, or does it provide refined support for the established timeline of hominin occupation in the Tajik depression? I believe it is the latter. The authors should frame the significance of their findings within a more modest and regionally grounded context. In its current form, the background lacks sufficient detail and there is a misalignment between the solid evidence and an overstated interpretation.

Response: We thank the reviewer for the positive comment and thoughtful feedback. We have tried to address the points raised by the reviewer in the revised version of the paper.

Response to Reviewer 2

Reviewer 2 summary of assessment
This manuscript entitled "First absolute chronology of the Early Palaeolithic in Central Asia" presents a novel and robust chronological framework for the Early Palaeolithic Karatau Culture in southern Tajikistan, Central Asia. The high-resolution temporal anchors of these key sites offer crucial insights into the evolution and migration of Mid-Pleistocene hominins across the Asian continent. This study is well-suited for publication in Nature Communications pending moderate revisions.<br><br>The paleomagnetic data from source references, which is one of the vital evidences to support the chronological conclusion of this manuscript, have been carefully evaluated. Those rock magnetic and magnetostratigraphic results integrate well with the new luminescence and cosmogenic 26Al-10Be ages presented in the manuscript. Nevertheless, several minor issues require attention and further elaboration:

Response: We thank the reviewer for the kind words and valuable feedback on the manuscript. We have revised the manuscript accordingly and provided detailed responses to the comments below.

Reviewer 2 Comment 1
Was the Khovaling Loess Plateau affected by tectonic activity since the Mid-Pleistocene? If so, it should be clarified whether the loess-paleosol strata in this area have experienced tilting.

Response: This is a very relevant question, but there is no evidence for tilting of the loess-paleosol strata. The loess and the soil units in the KLP are more-or-less horizontal throughout the area (as seen in Fig. 2), except where they follow local changes in the topography (mainly at Kuldara). This question was discussed in some of the early papers by Dodonov and Ranov, who concluded that there is no evidence for tilting of the loess-paleosol strata that are younger than 1 Ma.

Reviewer 2 Comment 2
The three loess-paleosol sections are located within the same plateau region. Could the authors provide the elevations of the three sections and confirm whether they all developed on the same river terrace level?

Response: Thank you for this comment. Both Kuldara and Obi-Mazar have a surface elevation of 1300 m, while Khonako-II was deposited higher up in the landscape with a surface elevation of 1880 m (Fig. 2 A). The question concerning river terraces is more complicated. The loess-paleosol section at Obi-Mazar rests on fluvial sediments next to the modern Obi-Mazar River. It is less clear what lie directly underneath the sections at Kuldara and Khonako-II, but they did not develop next to a large modern-day river. Over time, they were exposed by local rivers cutting into the loess-paleosol strata.

We have now added the surface elevations of the sites in table S5, including the latitude and longitude, as these numbers have been used to calculate the cosmogenic nuclide post-burial production. We have also added the following to the caption of figure 2:

Both Kuldara and Obi-Mazar have surface elevations around 1300 m, whereas the surface elevation at Khonako-II is 1880 m (Table S5). The surface of the sections at Obi-Mazar and Khonako-II is sub-horizontal, whereas the surface is sloping at Kuldara because it is cut by a gulley.

Reviewer 2 Comment 3
Several sedimentary hiatuses identified from Kuldara section (at depth interval of 20–11 m) are not observed in the neighboring Obi-Mazar section. What factors account for the gaps and the differential preservation in sedimentary continuity between these two sections?

Response: This is a very relevant question. At Kuldara, which is located in a side valley next to the Obi-Mazar River, there are a couple of local gulleys, and it is likely that erosion took place in these gulleys from time to time. Also, the present topography at Kuldara is more undulating as a result of erosion in these gulleys. This represents a stark contrast to Obi-Mazar and Khonako-II where the surface is flat, and the layers can be tracked over large distances. However, we note that there is no evidence for large hiatuses at Kuldara apart from those identified in this study (PC3, L3, PC2.1), as the number and estimated ages associated with the loess-paleosol units below 250 ka match our age-depth models for the two other sites.

Reviewer 2 Comment 4
The lock-in depth model is used in the Supplementary file (section 4.1.4) to explain the offset of the M/B boundary in loess deposits relative to marine records. Why other potential factors, such as magnetic minerals differences or pedogenic process, were excluded from consideration?

Response: This is a good question. The lock-in depth is observed in many loess sections across the world, and it is caused by delayed acquisition related to pedogenic processes in loess and not a difference in magnetic minerals. We have elaborated on this aspect in section 4.1.4 of the supplementary material to clarify this.

Reviewer 2 Comment 5
Considering the close archaeological connections between Central and East Asia, the discussion (lines 418–420) could be strengthened by incorporating recent chronologic advances and inter-regional comparison. In fact, many contemporaneous palaeolithic sites in China were well-constrained by different dating methods during last decade. Here are some literatures that might be helpful.<br>a) Ao H, Liu C, Roberts A P, et al. An updated age for the Xujiayao hominin from the Nihewan Basin, North China: Implications for Middle Pleistocene human evolution in East Asia. Journal of Human Evolution, 2017, 106: 54–65. DOI: 10.1016/j.jhevol.2017.01.014<br>b) Yang S, Deng C, Zhu R, et al. The Paleolithic in the Nihewan Basin, China: Evolutionary history of an Early to Late Pleistocene record in Eastern Asia. Evolutionary Anthropology, 2020, 29 (3): 125–142. DOI: 10.1002/evan.21813<br>c) Ji H, Liu C, Yin G, et al. ESR dating of the Hougou Paleolithic site in the Nihewan Basin, North China, using both additive and regenerative dose methods. Quaternary International, 2022, 619: 72–80. DOI: 10.1016/j.quaint.2021.10.001<br>d) Michel V, Jourdan F, Moncel M-H, et al. High-precision 40Ar/39Ar dating of Australasian tektites associated with bifacial tools in the Bose Basin (Xiaomei and Fengshudao sites), South China and in Vietnam (Go Da and Roc Tung 1 sites). Quaternary Science Reviews, 2024, 346: 109065. DOI: 10.1016/j.quascirev.2024.109065<br>e) Ye Z, Pei S, Tu H, et al. 26Al/10Be burial dating and technological strategies of hominins at the Jijiazhuang Paleolithic site, Nihewan Basin, China: Implications for understanding Middle Pleistocene human adaptations in east Asia. Quaternary Science Reviews, 2024, 339: 108837. DOI: 10.1016/j.quascirev.2024.108837

Response: Thank you very much for these suggestions! We have added these in the introduction and the discussion as well as figure 1.

Reviewer 2 Comment 6
(Lines 66–71, Figure 1) It would be helpful to include age constraints for the sites in the figure or caption. The current figure caption mislabels Sites 5 and 6. Site 5 should be “Nihewan Basin” in northern China and Site 6 should be “Niujianbao” (not “Niujianbuo” as in the text) site in southwestern China.

Response: Thank you for noticing this! We have corrected these errors and updated figure 1 substantially.

Reviewer 2 Comment 7
(Line 135) Should be “magnetostratigraphic”.

Response: Corrected

Reviewer 2 Comment 8
(Line 241, Figure 5) Magnetic susceptibility curves in column B are overlaid, which impedes clear visualization.

Response: We thank the reviewer for this comment. The magnetic susceptibility curves have now been separated in panel B in Figure 5 to avoid overlapping and improve clarity.

Reviewer 2 Comment 9

(Supplementary file, Line 488) Should be “stratigraphic”.

Response: Corrected

Response to Reviewer 3

Reviewer 3 summary of assessment

Sorensen et al present a detailed geochronological study of a loess-paleosol sequence combined with a detailed archaeological analysis of the lithic artefacts contained in this sequence. The time-span covered by this terrestrial sediment sequence is extraordinary, the geographical position of the site, that is situated in the Khovalin loess plateau in Tajikistan, is critical for understanding human migration pathways out of Africa and into the Asian continent throughout the Pleistocene.
The study rests on a geochronological dataset that is exceptionally large in the upper part of the loess-paleosol sequence (ca. 0-300 ka), where it is based on luminescence dating, and CRN dating of lithic artefacts and paleomagnetics on the loess section itself for the lower part of the section (from ca. 400 ka to 1 Ma). An age model is constructed and tested that combines the various numerical approaches and also integrates the cosmogenic production and erosion history of the pebbles that were ultimately used for artefact production.
This review focuses on the georchronological aspects and the overall structure of the manuscript. The geochronological methods are sound and well tested and carefully interpreted in terms of paleo-climate and sedimentation rates and archaeological implications. The story and data presented by Sorensen et al are significant and worthwhile publishing in Nature Communications, however, there are several points that need improvement to enhance the clarity of the manuscript and the logic of arguments, particularly in the main text of the paper. One key issue is the following:

Response: We thank the reviewer for their thorough and positive assessment of our study, as well as the constructive feedback. We have revised the manuscript accordingly and detailed responses to the comments are provided below.

Reviewer 3 Comment 1

Part of the dataset and key methodological foundations have already been published in Buylaert et al. (2024) and Cheallier et al. (2024). While the supplementary materials for the present manuscript contain the relevant methodological details, the main text currently lacks clarity and cohesion. Specifically: (i) it is often unclear whether statements and results attributed to “Sorensen et al.” refer to the authors’ new analyses versus previously published work (Buylaert et al., 2024; Cheallier et al., 2024); (ii) logic gaps arise because claims in the main text are not consistently linked to the relevant figures and/or supplementary sections; and (iii) the manuscript assumes substantial prior knowledge of the Khovalin loess plateau stratigraphy and chronology (across multiple sites), which needs to be explicitly articulated to be accessible to readers.

Response: Thank you for pointing this out. We have tried to address the comments below and ensure that (i) it is clear when we refer to the published data by Buylaert et al., 2024 and

Challier et al. 2024 or new data, (ii) we make clear links to figures and the supplementary, and (iii) provide enough background information on the Khovaling Loess Plateau.

Reviewer 3 Comment 2
Another aspect if related to the uncertainties that arise from the various dating and modeling exercises that should be stated more clearly, and also stated in relative terms (rel err) where relevant, to make the various age claims, rates etc intercomparable and better understandable. The numbered remarks below (in manuscript order) address these issues as well as additional methodological and minor points.

Response: Thank you for this suggestion. We now also present comparisons of numbers (rates and ages) in relative terms. Mostly in percentage. This has improved the manuscript and provides clarity to the respective statements.

Reviewer 3 Comment 3
Line 81: uppermost part = which PCs/LCs/ age range?

Response: The sentence now read:

a notion that was recently confirmed for the upper ~250 ka of the sections at Kuldara and Khonako using high-resolution luminescence dating.

Reviewer 3 Comment 4
Line 121 + 123: ages of Karatau Culture are your findings? From some other authors? Cite / clarify

Response: Thank you for this comment. We have revised these sentences and provided the relevant references:

The Karatau Culture is so far constrained in time by a younger limit of ~250 ka[1], provided by the luminescence ages associated with PC2 (and L3), and an older limit of ~770 ka provided by the Matuyama-Brunhes boundary[2,3,4]

*1. Buylaert, J.-P. et al. A luminescence dating study of the upper part of the loess-palaeosol sequence at Kuldara, Khovaling Loess Plateau, Tajikistan. Quaternary Geochronology **82**, 101545 (2024).*

2. Kulakova, E. & Kurbanov, R. First Paleomagnetic Record of the Matuyama-Brunhes Transition in the Loess-Paleosol Series of Tajikistan. in Problems of Geocosmos—2022 (eds. Kosterov, A., Lyskova, E., Mironova, I., Apatenkov, S. & Baranov, S.) 67–84 (Springer International Publishing, Cham, 2023). doi:10.1007/978-3-031-40728-4_6.

3. Penkov, A. V. & Gamov, L. N. Paleomagnetic benchmarks in the Pliocene-Quaternary strata of Southern Tajikistan. Granitsa neogena i chetvertichnoi sistemy. M.: Nauka (Publ.) 184–189 (1980).

*4. Kulakova, E. P., Kurbanov, R. N. & Sosin, P. M. New high-resolution magnetostratigraphic record of the last 1.2 Myr from loess-paleosol section Khonako-II in Southern Tajikistan, Central Asia. Quat. Sci. Rev. **371**, 109689 (2026)*

Reviewer 3 Comment 5
Line 133: 16 CRN pairs … provide a minimum of context and explanation – measuredon what? Refer to supplement section and ev. Figs there; provide citation for this dating principle (which is currently rather exotic) here too if appropriate.

Response: We have revised the sentences as follows:

We also provide an exceptionally detailed absolute chronology for three sections from the Khovaling Loess Plateau (Obi-Mazar, Kuldara, and Khonako-II), based on 286 luminescence ages from loess and soil samples, 16 ^{26}Al-^{10}*Be cosmogenic nuclide concentration pairs from quartz-bearing lithics, and paleomagnetic polarity records based on loess and soil samples (see sections 1-3 in Supp. Mat.).*

Reviewer 3 Comment 6
Line 153: what is the "citron technique"? pls explain or replace with more common terminology or add citation to an accessible paper the reader can refer to.

Response: Thanks for pointing this out. We now provide a brief description of the citron technique in the main text (*There are many 'citron' flakes (up to 8%), i.e. lemon shaped wedges produced during pebble flaking and the striking platforms are generally flat or cortical*) and a slightly longer description in the supplementary material.

Reviewer 3 Comment 7
L 184: Section age-depth models ◊ refs to supplement non existing!

Response: We have now added several references to the supplementary material throughout this section.

Reviewer 3 Comment 8
L 185: what precisely do you mean with "upper parts" – refer to Fig where it can be seen, do you still talk in terms of ref 17 and 18 or your own study?

Response: We now refer to figure 4 panels F, L and Q which shows the 'upper part' of the sections covered by luminescence ages. Here, we refer to our own study, which hopefully is clear after the insertion of the description of figure 4. We have also replaced "upper parts" with "upper ~250 ka" to clarify this statement.

Reviewer 3 Comment 9
L 188 and 190 – what is the error on these erosion numbers!?

Response: Yes – it is a good idea to include these errors. We have calculated and added the mean and std of the accumulation rates.

Reviewer 3 Comment 10
L 185-204: how does loess accumulate in your opinion during PC formation? is there accumulation that can be resolved within error? Or only pedogenic overprinting or older/pre-existing loess?

Response: This is a very relevant question. From a soil-formation standpoint, PCs in the Khovaling Loess Plateau are best interpreted as time-integrated soil-forming intervals, rather than instantaneous depositional events. Aeolian dust input likely continued episodically during PC formation, but sedimentation rates were strongly reduced relative to loess units and were consistently outpaced by pedogenic processes. Any syn-pedogenic dust input would therefore have been rapidly incorporated into the soil matrix through bioturbation, carbonate redistribution, and clay translocation, and cannot be resolved within the uncertainty of the absolute chronology.

This interpretation is directly supported by detailed pedological and micromorphological evidence from the Khonako II loess–paleosol section, where individual PCs (e.g., PC3 and PC5) comprise multiple ABk–Btk–Bt–Bk successions with diffuse boundaries, strong blocky structures, intensive carbonate redistribution, and abundant clay coatings. These features require prolonged soil stability and low net sediment accumulation (Talebi et al., *Quaternary International*, accepted).

Additional independent support comes from high-resolution $\delta^{13}C$ studies of pedogenic carbonates in modern loess-derived soils from northern Iran (Khormali et al., *Quaternary Research*, in revision). In that study, $\delta^{13}C$ values of pedogenic carbonate show very strong, systematic relationships with MAP (mean annual precipitation) and P/PET (aridity index), indicating that carbonate formation integrates soil respiration, vegetation structure, and moisture balance over extended periods of soil stability. Such coherent isotopic signals would be difficult to preserve under conditions of rapid or continuous sediment accumulation, reinforcing the interpretation that pedogenic carbonate formation—and by extension PC development—occurs under low sedimentation rates and time-integrated soil processes.

From an archaeological perspective, this pedogenic framework implies that artifact-bearing PCs represent stable or slowly aggrading land surfaces, where repeated occupations may be incorporated into soils forming over long timescales. This can result in some degree of time-averaging, while still allowing preservation of stratified artifact concentrations at the centimeter scale.

We hope this addresses the points.

Talebi, F., Khormali, F.*, Karimi, A., Tazikeh, H., Kurbanov, R., Buylaert, J.-P. *Paleopedogenesis and paleoclimatic changes recorded at the Khonako II loess section, Khovaling Loess Plateau, Southern Tajikistan.* Quaternary International, accepted.

Khormali, F.*, Tabor, N., Robbins, J. A. *High-Resolution Isotopic Signatures of Pedogenic Carbonates in Loess Soils of Northern Iran: A Reliable Proxy for Holocene Soil Water Balance and Ecosystem Dynamics.* Quaternary Research, in revision.

Reviewer 3 Comment 11
L 199: hiatuses – can they been specified? Horizon of non-deposition, erosional hiatus..?

Response: They are presumably erosional hiatuses, as they coincide with erosional boundaries (red lines in figure 4). We have added a sentence in the introduction to address this:

High-resolution luminescence dating of the upper ~250 ka indicates the presence of significant hiatuses that coincide with erosional boundaries (Buylaert et al. 2024).

Reviewer 3 Comment 12
L 205 + 207 : The upper parts … versus lower parts … ? Refer specific to these parts via Fig., or

Response: We now write *upper ~250 ka (Fig. 4F, L, Q)* and *lower >~250 ka (Fig. 4C, I, O)* instead of *upper parts* and *lower parts*, respectively, to signify the age range datable by luminescence dating and specifically referring to the panels showing this part.

Reviewer 3 Comment 13
L 209: (Fig. 4F and 4Q, Challier, 2024) ◊ unclear what are your and Chevallier et al data. (also: et al missing)

Response: Thank you for pointing this out. The reference to Challier 2024 here was a mistake, because the age intervals are derived from the age-depth models presented in this study (Sørensen et al.) and shown in Fig. 4. The reference to Challier et al. has now been removed.

Reviewer 3 Comment 14
L 209: you cite your Fig for the first time starting with F, would be better to introduce A, B etc much earlier. Explain your site, stratigraphy, chronology step by step rather than assuming everybody knows papers 17 and 18 already.

Response: We accept his point and in response to this comment, we now start the section entitled "Age-depth models for the Khovaling Loess Plateau" with:

The age-depth models associated with the Obi-Mazar, Kuldara, and Khonako-II profiles are presented in figure 4 along with the lithology (Fig. 4A, G, M), magnetic susceptibility (Fig. 4B, H, N), and the data that constrain the age-depth models (Fig. 4C, I, O). The upper ~250 ka of the loess-paleosol sections, which are temporally constrained by luminescence ages (Fig. 4F, L, Q), indicate variable dust accumulation rates across time and space.

Reviewer 3 Comment 15
L 220: ..within the luminescence age range .. ◊ what is the uncertainty (for the OSL ages/the mode)?

Response: The sentence now reads:

The age uncertainty (1σ) of the age-depth models for stratigraphic units located below the luminescence age range is generally considerably larger (30-50 ka, Table S7) than for units located

within the luminescence age range (upper ~250 ka) which shows 1σ uncertainties of ~1-20 ka (Fig. 4).

Reviewer 3 Comment 16.1
L 223: Figure 4:<br>this Fig is key and should be a stand-alone Figure, i.e. needs to be better integrated/explained step-by-step in main text and in Fig caption + reference to supplement for details.

Response: See response to comment 14.

Reviewer 3 Comment 16.2
Unclear how transposing of ages and colour coding works (line 231 – transposed ages = which ages?)

Response: The sentence now reads:

Colored ellipses represent transposed ages from the individual age-depth model of Obi-Mazar (red), Kuldara (green) and Khonako-II (blue), with width indicating age uncertainty (see Fig. S3–5) and height indicating depth uncertainty (see Figs. S6–S8).

The color coding is also illustrated in the bottom bar of figure 4.

Reviewer 3 Comment 16.3
The symbol for the magnetostratigraphic ages is very hard to read in the A4 print out version in Fig 4 I and O.

Response: We have changed the symbols for the magnetostratigraphic ages by removing the cross and the size of error bar caps. Hopefully, these symbols are easier to read in an A4 print out version of the figure.

Reviewer 3 Comment 16.4
The CRN concentration pairs are important and need a minimum on explanation here already + ref to Suppl. (e.g line 229) – what type of samples? do they come from PC 4, 5 and 6? How do they fold into the model – i.e. do they project also onto the x-axis (age in Ma)?

Response: We thank the reviewer for pointing out the need for clearer explanation of the cosmogenic radionuclide. The explanation of Panels D, J and P now reads:

*Panels **D**, **J**, and **P**: measured $^{26}Al/^{10}Be$ ratios from lithics collected during multiple expeditions between 1976 to 2021 from PCs 4-6 (Table S4). The y-axis shows the depth, while the x-axis shows the $^{26}Al/^{10}Be$ ratios which decrease with depth reflecting increasing burial age with depth. These ratios are incorporated into the age-depth models by calculating the $^{26}Al/^{10}Be$ ratios as a function of the exposure burial history, as detailed in the supplementary material and Sørensen et al.2024*

Reviewer 3 Comment 17
L 236: ages of Karatau cultural layers derived from this age model? From other archaeological publications (cit)?

Response: Yes, from the age-depth models presented in our study. We have added “derived from the age-depth models” to the sentence to clarify this:

Colored error bars mark the ages for the mid-depth of Karatau Culture layers PC 4-6 derived from the age-depth models obtained from Obi-Mazar (red), Kuldara (green), and Khonako-II (blue).

Reviewer 3 Comment 18

L 266: significantly enhances the precision ◊ by what? Specify.

Response: The specific enhancement of the precision depends on the depth at the site. That is why we in the following sentences specifically state how much the vertical coupling enhances the precision at PCs 4-6 compared to the conventional burial dating methods.

Reviewer 3 Comment 19

L 270 + 272: here and on several other occasions: give relative error as well to make it

Response: We have added information regarding the relative error and how much it changes as the model approach integrates more and more information. Note that the exact numbers depend on the PC – we provided a representative example based on PC5.

Reviewer 3 Comment 20

L 277: P-DTW = ?

Response: Thank you for noticing this. We have now added:

Probabilistic Dynamic Time Warping (P-DTW)[54]

Reviewer 3 Comment 21

L 279: the age of PCs 4-6 to 35 ka ◊ provide relative error too

Response: Done – we have now included this information.

Reviewer 3 Comment 22

L 287: the new luminescence ages ◊ published versus new data (this study)… n=?

Response: Here, we only refer to the new luminescence ages. We have added numbers and references in brackets:

Additionally, the new luminescence ages from Obi-Mazar (n=40) and Khonako-II (n=145, Table S1) support the notion that pedocomplexes PC1 and PC2 were formed during MIS 5 and MIS 7, respectively, which is consistent with the luminescence ages from Khonako-II1[1]

1: Challier, A. Detailed quartz and feldspar luminescence chronologies of loessic Palaeolithic sites in Tajikistan. PhD thesis. Department of Physics, Technical University of Denmark. https://backend.orbit.dtu.dk/ws/portalfiles/portal/390510697/PhD_thesis_-_Am_lie_Juliette_Marie_Challier.pdf. (2024).

Reviewer 3 Comment 23

L 293: is too large ◊ add error in %

Response: When revising L 293, we realized that the sentence was an understatement and we have therefore expanded this part regarding the older PCs (PCs 7-12):

The ages associated with the older pedocomplexes (PCs7-9) are consistent with the ages of interglacial periods MIS 17-19 (Fig. 5). Notably, PC8 correlates with an even-numbered MIS, hereby breaking the otherwise apparent correspondence between pedocomplex development and odd-numbered stages. Even-numbered stages are generally classified as cold periods, but MIS 18 is different as it contains a warm interval in the middle of the glacial period (as shown by the marine $\delta^{18}O$ record). This observation underscores the importance of independent absolute chronologies for loess profiles such as those from the KLP. Furthermore, the ages of PC11 and PC12, associated with the Kuldara assemblages, most likely correspond to MIS 23 and MIS 25 (~915 and ~970 ka, respectively; Fig. 5), thereby representing the earliest, dated evidence of hominin occupation in Central Asia.

Reviewer 3 Comment 24
L 313: Fig. 5 ◊ integrate and cite at beginning of section (and whenever required in-between), not at the end;

Response: We have added a reference to Fig. 5 at the beginning of the section, as well as references to Supplementary Tables S9–S11 related to the lithic analysis, which are also relevant here.

Reviewer 3 Comment 25
L 358: MIS 9 ◊ but there are 2 artefacts from that section (attributed to bioturbation in the supplement) – what about 3D spatial heterogenous pattern of camp-sites versus 2D outcrop?

Response: Yes – we discuss this observation in the section entitled “Presence of early humans in the Khovaling Loess Plateau area through time”. We cannot rule out definitively that the two artefacts in PC3 (MIS 9) can be attributed to bioturbation, but in this section, we argue that vertical mixing is unlikely. It is a possibility that there are 3D spatial heterogeneities, but we note that PC3 has been excavated at multiple sites in the area over many years (and only 2 artefacts have been found), so it is very unlikely that there was a thriving culture in this region during MIS 9. Also, two artefacts are way too little to describe the culture and make comparisons to the Karatau Culture defined by the lithics in PC4-6. In conclusion, we think the area was largely devoid of human activity during MIS 9, but there may have been a migratory presence of a small number of humans that temporarily stayed in the area (as discussed at the end of this section). In response to this comment, we have modified the discussion in the supplementary material.

Reviewer 3 Comment 26
L 367 onward: this paragraph seems to link directly with the paragraph L349-358, merge!?; disappearance ◊ during glacials? During MIS 9?

Response: Yes – it is true that these two paragraphs are linked. We considered this suggestion but decided to keep the original order of the paragraphs. The last paragraph in this section is longer and more speculative, and we think it is better placed at the end. Also, it gives a better lead-up to the final section.

Regarding the disappearance during glacials / MIS 9, we believe that the Karatau Culture disappeared with the onset of the glacial associated with MIS 10, and then they didn't reappear during the subsequent interglacial (MIS 9. In general, the environment during glacials were much less suitable for early humans, with MIS 14 and MIS 6 as exceptions to this rule.). In the paper, we discuss possible reasons for the disappearance of the Karatau Culture at the onset of MIS 10, as well as possible reasons for why the culture didn't reappear during MIS 9.

Reviewer 3 Comment 27
L 382: the disappearance of the archaeological culture after PC4/MIS 11. ◊ but there is archaeology throughout the entire section! Be more clear/explicit about the Karatau culture (which you are refering here to I guess) versus other cultures – show this in Fig 5 more explicitly too.

Response:

We now highlight the interval associated with the Karatau Culture in Fig. 5 and also differentiate it from the Kuldara assemblages and the Middle and Late Paleolithic intervals. In the text, we have also made sure that we are more explicit about the Karatau Culture here.

Reviewer 3 Comment 28
L 70: site 12 = ?

Response: Yes – thanks for pointing this out. We have now redone Fig. 1 along with the associated caption.

Supplement:

Reviewer 3 Comment 29
L 39: ref 7 is about Q OSL, you extract polymin "FG" for IRSL.

Response: Ref 7 (Murray et al. 2021), even though it refers to quartz, is used in this context to refer to standard sample preparation techniques: e.g. HCl, H2O2 and sieving. But we have changed the reference to Challier et al. (2024) instead to avoid confusion.

*Murray, A. et al. Optically stimulated luminescence dating using quartz. Nat Rev Methods Primers **1**, 72 (2021))*

*Challier, A. et al. A detailed quartz and feldspar luminescence chronology for the Khonako II loess section (Southern Tajikistan). Quaternary Geochronology **83**, 101571 (2024).*

Reviewer 3 Comment 30
L 178: these samples – which ones?

Response: L 178 now reads:

All these excluded samples are reported in table S4 for any future studies to use.

Reviewer 3 Comment 31
L 179: Samples used for the age-depth model… n=?

Response: We have added the numbers in brackets.

Reviewer 3 Comment 32
L 180: S3 = Fig Table?

Response: It refers to Figure S3. We have corrected this here and at two other locations.

Reviewer 3 Comment 33
L 341: which delays the acquisition of a stable signal – in loess or marine sediments?

Response: Good question. This refers to the loess, which we have now clarified in the text.

Reviewer 3 Comment 34
L 385: range generally increase ◊ how much in %?

Response: From ~10-150% depending on the site and the pedocomplex (Table S7). We have written:

We tested the sensitivity of the age-depth models to different datasets. When constructing models using only luminescence ages and magnetostratigraphic boundaries (excluding ^{26}Al and ^{10}Be concentrations), the age uncertainties in the PC4-6 depth range generally increase and from ~10-150 % depending on the site and the pedocomplex (Fig. S3 C, I, O, Table S7), compared to the age-depth models that includes all the data (Fig. S3 E, K, Q, Table S7).

Reviewer 3 Comment 35
L 393: uncertainties in the upper parts of the profiles increase ◊ how much in %

Response: The increase is up to ~1000% depending on the depth and profile. In the absence of OSL ages, the age of the younger parts (<250 ka) of the profiles are constrained by the $^{26}Al/^{10}Be$ concentrations and the associated uncertainties are large because this

method is relatively insensitive in the age range 0-200 ka (due to the half-lives of ^{26}Al and ^{10}Be). Also, it is the integrated approach in the CosmoChron model, which combines OSL with $^{26}Al/^{10}Be$, that really strengthens the use of the $^{26}Al/^{10}Be$ in this context. The sentence now reads:

Conversely, when constructing models using only ^{26}Al and ^{10}Be concentrations (excluding luminescence dating and magnetostratigraphy), the uncertainties in the upper ~250 ka of the profiles increase up to ~1000% (age-depth models shown in Fig. S3 D, J, P relative to those shown in Fig. S3 C, I, O), as these layers are primarily constrained by luminescence ages in the full model.

RESPONSE TO THE REVIEWERS' COMMENTS

Reviewer #1:

Thank you for the opportunity to review this manuscript. I am happy with the revision and I feel the authors have addressed my concerns. I recommend this paper for publication.

Response: We thank the reviewer for the positive evaluation and for recommending our manuscript for publication. We appreciate your constructive feedback throughout the review process.

Reviewer #2:

The authors have thoroughly revised their manuscript and provided detailed responses to the reviewers' comments. In my view, the manuscript is significantly improved and is suitable for publication subject to the following minor revisions:
Figure 1 Caption: (1) correct the name of Site 8 to “Niujianbao,” as previously noted. (2) Correct the name of Site 18 to “Shuangshu.”(3) Both Site 11 and Site 23 are currently labeled as “Bose Basin.” Please replace one of them with a more specific site name.

Response: We thank the reviewer for these helpful comments. We have corrected Site 8 to “Niujianbao” and Site 18 to “Shuangshu” in Figure 1. To avoid duplication, Site 11 is now specified as “Bose Basin (Xiaomei and Fengshudao)” to distinguish it from Site 23.

Figure 4: It is suggested to add numerical scales and labels to the horizontal axes in the diagrams of Obi-Mazar and Kuldara sections.

Response: We thank the reviewer for this suggestion. Numerical scales and labels have been added to the horizontal axes of the Obi-Mazar and Kuldara sections in Figure 4.

Reviewer #3:

The authors have addressed the questions and comments and I recommend this overhauled version of the manuscript for publication in Nat.Comm.

Response: We thank the reviewer for the positive assessment and recommendation for publication.
