# Supplementary material for: Absolute chronology of the Early Palaeolithic Karatau Culture in Central Asia
Source: Nat Commun. 2026 Jul 1;17:8217. doi: 10.1038/s41467-026-74938-5 (PMC13462899; doi:10.1038/s41467-026-74938-5)
Supplement: Supplementary file 2 [file 41467_2026_74938_MOESM1_ESM.pdf]

Supplementary Information for “Absolute chronology of the Early Palaeolithic Karatau Culture in Central Asia”

This supplementary material provides additional background information on the data and methods used to construct the age–depth models presented in Figure 4 of the main text. It is organized into five main Supplementary Notes:

1. *Luminescence chronology*
2. *Magnetostratigraphy*
3. *Cosmogenic nuclides*
4. *Construction of the age-depth models*
5. *Detailed description of the Early Paleolithic Karatau Culture*

Supplementary Note 0: Overview of the data used to construct the age-depth models

The age-depth models associated with the three loess sections at Obi-Mazar, Kuldara, and Khonako-II are constructed by integrating luminescence (OSL, post-IR_{200}, and $IRSL_{290}$) ages, cosmogenic $^{26}Al/^{10}Be$ concentrations, and paleomagnetic boundaries into CosmoChron, a probabilistic inverse age-depth model. Table S1 shows an overview of the data used to construct the age-depth models displayed in figure 4 and S3. The luminescence ages are used to constrain the age-depth models in the upper ~250 ka; the cosmogenic nuclides samples are used to constrain the age-depth models in the 300 to 600 ka age range because the majority of stone tools derive from PC4, PC5, and PC6. The older parts (>700 ka) of the age models are constrained by paleomagnetic boundaries. The magnetic susceptibility records are used to refine and augment the age-depth models in the ~300–700 ka range, as explained in Supplementary Notes 4.3 and 4.4.

Table S1: Overview of the data included in the age-depth models shown in figure 4.

		Obi-Mazar/ Lakhuti	Kuldara	Khonako-II	Total
New data	Luminescence ages	40	0	145	185
	Cosmogenic nuclides samples	11	2	3	16
	Magnetic susceptibility	1223	0	0	1223
Published data	Luminescence ages	0	78 [1]	23 [2]	101
	Paleomagnetic boundaries	0	1 [3]	3 [4]	4
	Magnetic susceptibility	1094 [5]	2861 [6]	1038 [4]	4993

The luminescence, cosmogenic nuclide, magnetic susceptibility and biomarker data used to produce figure 4 and 5 are provided in the Supplementary Data and in FigShare (https://doi.org/10.6084/m9.figshare.31249798). Supplementary Data files includes the luminescence data (Supplementary Data 1–3), cosmogenic nuclide data (Supplementary Data 4–8), and magnetic susceptibility data (Supplementary Data 9–11). Furthermore, the age-

depth models shown in figure 4, as well as the biomarkler data presented in figure 5, are also included in the supplementary data files (Supplementary Data 12–14 and Supplementary Data 15–17, respectively).

Supplementary Note 1: Luminescence Chronology

The three sections were sampled for luminescence dating at a high spatial density with distance between samples varying from 5 to 180 cm; the average distance between samples was 30–40 cm for Khonako II and Kuldara sections and 70 cm for Obi Mazar. Luminescence ages (n=85) for the entire Kuldara section (~26 m) have been published by Buylaert et al.[1]. The luminescence chronology for the upper part (~8 m, n=37) of the Khonako II section was published by Challier et al.[2]. In this work we add the luminescence data for another set of 140 new samples collected to improve the sampling resolution between 370 and 780 cm depth from Challier's data and to extend the Khonako II chronology down to the bottom of PC2. For the Obi Mazar site, 40 luminescence samples were collected from the upper ~30 m, covering the presumed L1 loess and PC1 and PC2 pedocomplexes.

Standard sample preparation techniques including HCl (10%) and H_2O_2 (10%) treatments and wet-sieving were used to extract the polymineral 40–63µm fraction from each sample[2]. These grains were mounted as large (~8 mm diameter) aliquots on stainless steel cups and measured in a standard Risø TL/OSL reader equipped with infra-red (IR, 870 nm) stimulation and a calibrated $^{90}Sr/^{90}Y$ beta source[7,8]. The chosen SAR post-IR IRSL protocol involved two 200 s IR stimulations, one at 200°C to remove unstable charge and a subsequent stimulation at 290°C to access that signal of interest ($pIRIR_{200,290}$). At end of each SAR cycle there was an elevated temperature IR (325°C, 200 s) cleanout step to reduce recuperation. This $pIRIR_{200,290}$ protocol has been applied successfully in several luminescence dating studies on loess (e.g. [10,11]). Following Challier et al.[2], the test dose size was kept at ~100% of the estimated dose for the Khonako II and Obi Mazar samples, whereas for the Kuldara data a test ranging between 30–50% was used[1]. The size of these test doses was chosen based on successful dose recovery tests.

A typical dose response curve is shown in Fig. S1a for sample 249038 collected below PC2 at Obi Mazar. It can be seen that the natural sensitivity corrected light level lies well below laboratory saturation and interpolation can be easily achieved. Fig. S1b shows the dose recovery data for the top sample from Obi Mazar (249001). In this experiment a range of laboratory doses was added on top of the natural dose and measured with SAR (using test doses approximately equal to the measured doses). The slope of the linear fit is 1.09 suggesting that our SAR $pIRIR_{200,290}$ protocol can accurately measure a dose given prior to a heat treatment.

It has been shown by Challier[11] and others (e.g.[10,13]) that the $pIRIR_{200,290}$ signal does not seem to fade in the laboratory and does not require correction for instability. However, the $pIRIR_{200,290}$ signal does contain a very-hard-to-bleach component which needs to be subtracted prior to age calculation. This presumed residual dose at deposition was estimated

by Challier et al.[2] to be 9.2±0.4 Gy based on very young loess and modern dust samples. The measured $pIRIR_{200,290}$ D_e values with this tresidual subtracted are given in Supplementary Data 1.

For each sample, the radionuclide concentrations (^{238}U, ^{226}Ra, (^{210}Pb), ^{232}Th and ^{40}K) were measured on dried and powdered subsamples using high-resolution gamma spectrometry following Murrey[13,14] (Supplementary Data 2). Radionuclide concentrations were converted to dry, infinite matrix, alpha, beta and gamma dose rates using the conversion factors of Cresswell et al.[15]. Alpha and beta attenuation factors follow Martin et al.[16] and Mejdahl[17], respectively. To allow for the different luminescence efficiency of alpha compared to beta/gamma radiation an a-value of 0.09±0.02 was adopted[18]. The individual dry dose rate components were corrected for moisture content using the correction factors proposed by Aitken[19]. For Khonako II and Obi Mazar the water content values are based on the depth-dependent water content model developed by Challier[11] for the Khonako III site. For Kuldara the water content values presented in Buylaert et al.[1] were used. A small internal beta dose rate from ^{40}K was incorporated (0.24±0.01 Gy/ka, based on 12.5% K in K-rich feldspar grains). The internal U, Th alpha dose rate contribution was assumed to be 0.10±0.05 Gy/ka. Cosmic ray dose rate contribution was calculated following Prescott and Hutten[20]. The total dose rates, water content values and final $pIRIR_{200,290}$ ages are also presented in Supplementary Data 1.

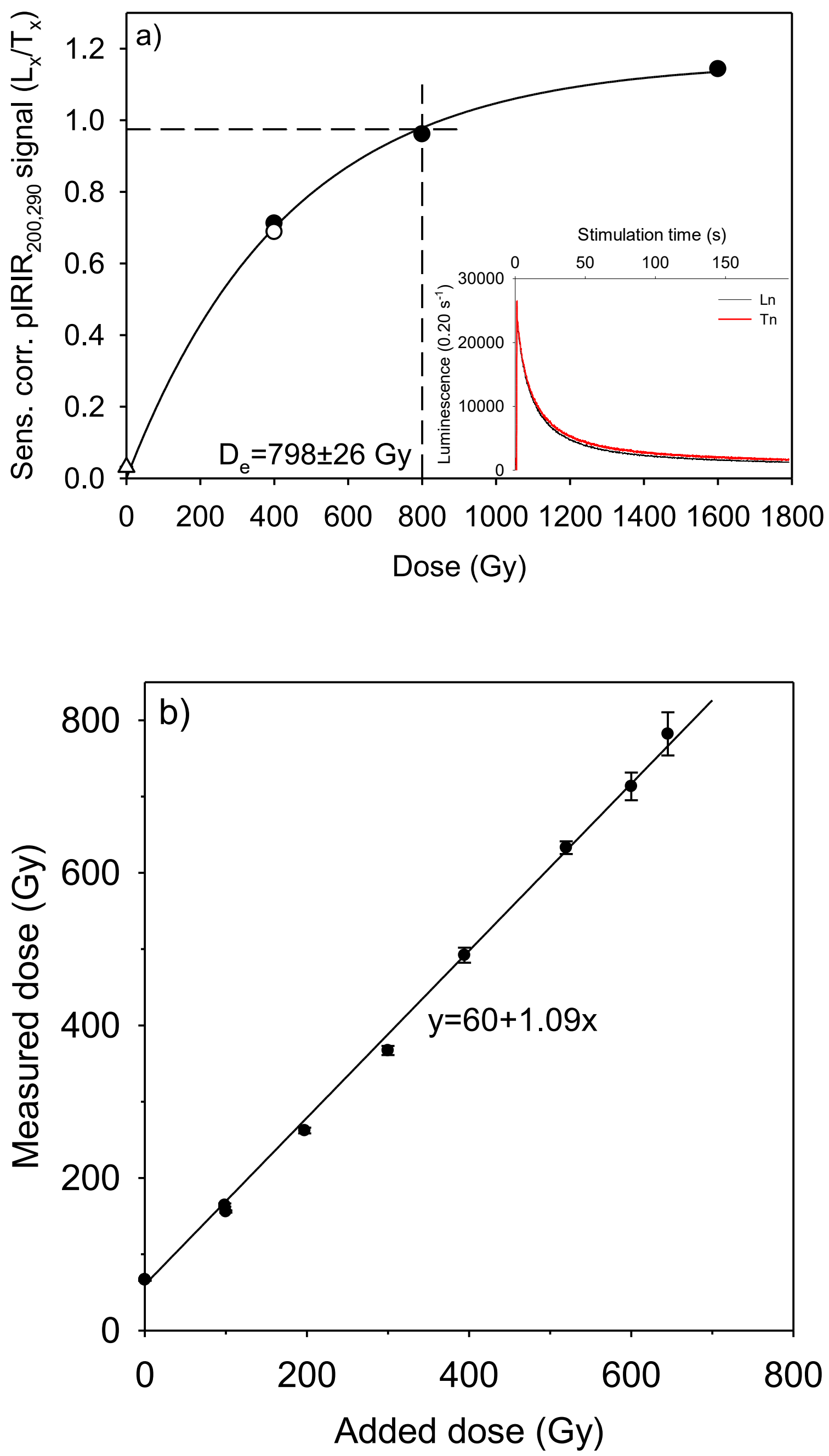

Fig. S1 a) Laboratory dose response curve for sample 249038 from the Obi Mazar section. The dose response curve is well-represented by a single saturating exponential function ($L=L_0(1-D/D_c)$) with a D_c value of (435±10 Gy). b) Dose recovery test on sample 243001 carried out by adding beta doses on top of the natural dose. Ratio of test dose to measured dose ranged from ~0.95 to ~1.20.

Supplementary Note 2: Magnetostratigraphy

Magnetostratigraphic studies conducted in 2021–2023 confirm that the upper nine pedocomplexes of loess-paleosol sequences in the Khovaling region are within the Brunhes chron[3,4,6]. A detailed study (with a resolution of 2–2.5 cm) of the paleomagnetic record of the last reversal of geomagnetic field – Matuyama-Brunhes (M/B) – was carried out for the Kuldara and Khonako-II sections. The Matuyama-Brunhes transition is recorded in the 2.9 m (depths 74.96–77.86 m) and 2.7 m (depths 95.52–98.20 m) thick interval at the Kuldara and Khonako-II, respectively, and is characterized by a decrease in the value of natural remanent magnetization, deterioration of the quality of the paleomagnetic record, and scattered directions in specimens from the same level[3,4]. The ending of M/B transition for both sections is fixed at the lower part of PC 9, corresponding to MIS 19[21], which was established for the first time for loess-paleosol series of Southern Tajikistan – previously M/B boundary occurred in loess unit L 10 (MIS 20)[22].

The upper and lower boundaries of the Jaramillo subchron were studied in detail at the Khonako-II section. The upper Jaramillo is identified for the first time within PC 14 (MIS 28b), and Lower Jaramillo was established at the same level as it was fixed before by A.V. Penkov, at the base of bottom of PC 16 (MIS 31)[4,22].

The new position of the paleomagnetic boundaries is in lithological units of the same climatic stages as in oceanic sediments, indicating that there is no significant lag in the response of continental and oceanic environments to global climate fluctuations. Nevertheless, it was found that the position of these boundaries is 1–1.5 m lower than expected, which is presumably related to the peculiarity of the mechanism of paleomagnetic signal fixation by the loess-paleosol series.

Supplementary Note 3: Cosmogenic nuclides

Archaeological artifacts used for measuring cosmogenic ^{26}Al and ^{10}Be were collected by V.A. Ranov in 1976, 1979 (Lakhuti-I[23,24]), 1981 (Kuldara[25]), 1982, 1986 (Karatau[26,27]), 1995, 1997 (Obi-Mazar[28]), 2000 (Khonako-III[29]), by T. Khujageldiev in 2021 (Kuldara[6]), by A.A. Anoikin in 2021 (Lakhuti-IV[30]) and stored at the Natural Museum of Tajikistan (Supplementary Data 4). Sample preparation was carried out at the Cosmogenic Nuclide Laboratory at Aarhus University, and the concentrations were measured at the Aarhus University accelerator mass spectrometry center.

Samples were initially crushed and sieved to a 250–500 µm grain size fraction, followed by magnetic separation. The grains were then treated with hydrochloric acid (HCl) to dissolve calcium carbonates, then cleaned in heated aqua regia to remove remaining impurities. Quartz and feldspar were separated by flotation and heavy liquate separation. The quartz fraction was then etched with hydrofluoric acid (HF) to remove meteoric ^{10}Be from the grain surfaces. Finally, ~20 g of quartz from each sample was fully digested in HF along with an in-house ^{9}Be and ^{27}Al carrier. Chloride conversion was achieved through repeated addition and evaporation of 6 M HCl. Beryllium and aluminum were then isolated using ion exchange

chromatography. The oxidized samples were mixed ~1:4 with Nb powder (BeO) and ~1:1.5 Ag power (Al_2O_3) and pressed into cathodes for AMS analysis.

The $^{26}Al/^{27}Al$ and $^{10}Be/^{9}Be$ ratios were measured using the multi-element AMS system at the Aarhus AMS Centre[31]. Beryllium results were normalized to the ICN standard 01-5-4, which has a certified $^{10}Be/^{9}Be$ ratio of 2.851×10^{-12} [32]; reported errors include a 1.1 % uncertainty associated with the standard. Aluminum results were normalized to the ICN standard 01-4-3, with a $^{26}Al/^{27}Al$ ratio of 10.65×10^{-12} [33]; uncertainties include a 1.2% contribution from the standard. For each batch, the $^{10}Be/^{9}Be$ and $^{26}Al/^{27}Al$ ratios of the corresponding procedural blanks were subtracted from the sample measurements.

LA293, OM67, KulPC11.1 and KulPC11.2 were excluded from the age-depth model as they represent modern samples with a $^{26}Al/^{10}Be \sim 6.8$, while OP6, Laalu1s, Laalu3s, Laalu4.1, and Laalu4.2 were excluded because the uncertainty on the concentrations are so high that the age-depth model does not benefit from including them, Laalu6s was excluded as the Be concentration measured with ICP-OES was higher than spike value, and lastly, KA1573 and Ka4, were excluded as we focuss on the Karatau site. All these excluded samples are reported in Supplementary Data 4 for any future studies to use. Samples used for the age-depth models for Khonako-II (n=3) and Obi-Mazar (n=11; Fig. 4 and Fig. S3) derive from parallel profiles from the same section. Geological units can be correlated by eye between the profiles at Lakhuti (Obi-Mazar and Lakhuti-I) and the same is true at Khonako (Khonako-II and Khonako-III). Specifically, the depths of the archeological artifacts have been noted in height above the petrocalcic horizon which is a well-defined layer that can easily be followed from Khonako-III to Khonako-II and from Lakhuti-I to Obi-Mazar.

Supplementary Note 4: Construction of the age-depth models

The age-depth models presented in figure 4 and S3 were constructed using the CosmoChron [34] method, a probabilistic inverse modeling approach that integrates multiple dating techniques. This method incorporates luminescence (quartz optically stimulated luminescence – OSL, and polymineral grains post-IR_{200} $IRSL_{290}$) ages, ages derived from paleomagnetic boundaries, and cosmogenic ^{26}Al and ^{10}Be concentrations. It also makes use of prior knowledge about: i) the loess accumulation process, such as the requirement that the ages must increase with depth, ii) the origin of the samples (Fig. S2), iii) the duration of hiatus(es) (Fig. S8), and iiii) the paleomagnetic lock-in depth in loess deposits (Fig. S9). Furthermore, the model incorporates age constraints by aligning magnetic susceptibility records across sites. These alignments are performed using the Probabilistic Dynamic Time Warping (P-DTW) algorithm[35], which also quantifies the uncertainty associated with the alignment (Fig. S4). This uncertainty is propagated into the CosmoChron age-depth model, and it may include multimodal or any other form of distribution resulting from the automated alignment of magnetic susceptibility records (Fig. S5–7).

An inverse problem seeks to constrain model parameters based on observed data, $\mathbf{d}_{obs}$. In the context of the CosmoChron models shown in figure 4, the model parameters **m**, include a series of accumulation rates **ϴ**, the age at the uppermost depth $\mathbf{T_0}$, the durations of hiatuses **h**, depths of transposed ages **Td**, paleomagnetic lock-in depths **Ld** and a pre-burial parameter **p** for each $^{26}Al/^{10}Be$ data point:

$\mathbf{m}=[\mathbf{\Theta}, \mathbf{T_0}, \mathbf{h}, \mathbf{Td}, \mathbf{Ld}, \mathbf{p}]$

The exact composition of **m** depends on the model setup. For instance, if the model includes only direct age constraints without hiatuses, transposed ages, or paleomagnetic boundaries, the parameter set simplifies to $\mathbf{m}=[\mathbf{\Theta}, \mathbf{T_0}]$. Constraining the series of accumulation rates **ϴ** is particularly critical for dating sedimentary profiles, as it directly governs the age-depth relationship of the profile.

The forward model describes how to compute the data **d** given the model parameters **m**:

$$\mathbf{d} = g(\mathbf{m}).$$

The output of the forward model is data consisting of $^{26}Al/^{10}Be$ ratios, transposed ages and direct age constraints at specific depths:

$\mathbf{d}=[\mathbf{^{26}Al/^{10}Be}, \mathbf{Transposed}\ \text{Ages}, \mathbf{Direct\ Ages}]$.

The inverse problem can be solved using the probabilistic framework of Tarantola[36], where the posterior probability distribution $\sigma_M(\mathbf{m})$, combines prior knowledge of the model parameters and the likelihood from observed data:

$$\sigma_M(\mathbf{m}) = k\rho_M(\mathbf{m})L(g(\mathbf{m})).$$

where $L(g(\mathbf{m}))$ is the likelihood and is a measure of how well the forward response of **m** reflects the observed data and $\rho_M(\mathbf{m})$ represents prior knowledge about the model parameters.

We sample the posterior distribution using the extended Metropolis algorithm[37], implemented though the SIPPI toolbox for Matlab[38].

The following sections detail the prior constraints applied to each model parameter used to construct the age-depth models shown in Figure 4 and S3, including accumulation rates **Θ** (Supplementary Note 4.1.1), hiatus durations **h** (Supplementary Note 4.1.2), pre-burial $^{26}Al/^{10}Be$ ratios **p** (Supplementary Note 4.1.3), and paleomagnetic lock-in depths **Ld** (Supplementary Note 4.1.4). The derivation of the prior transposed depths **Td** though alignment of magnetic susceptibility is discussed in Supplementary Note 4.4, with implementation details provided in Supplementary Note 4.5. These model parameter constraints together represent the prior model.

Supplementary Note 4.1: The prior CosmoChron model

As described above, the prior CosmoChron model represents constraints on the model parameters, and hence knowledge of the loess accumulation process, hiatus durations, transposed depth, the pre-burial history of the samples, and the magnetostratigraphic lock-in depth. It defines plausible source-to-sink transport pathways for the samples used for cosmogenic nuclide analyses, and it incorporates knowledge about the paleomagnetic lock-in process in loess deposits, alignment of magnetic susceptibility and hiatus durations.

A detailed description of the prior model is provided in the following section, with a summary presented in Tables S2–3, S5 and Figures S2–4.

Supplementary Note 4.1.1: Accumulation rate process

The mean prior inverse accumulation rate is estimated based on the depth of the Matuyama-Brunhes paleomagnetic boundary at Khonako-II, observed at ~95 m (Fig. 4, Table S3; [4]). This suggests a mean accumulation rate of ~0.12 m/ka, which we apply as the prior mean accumulation rate for all three sites. We use this constraint from Khonako-II for all sites, because such information is unavailable for Obi-Mazar, which is too young to include the Matuyama-Brunhes Boundary. At Kuldara, the Matuyama-Brunhes Boundary is observed at ~76 m, indicating a lower mean accumulation rate of ~0.1 m/ka. However, the mean accumulation rate in CosmoChron reflects the accumulation rate within intervals characterized by continuous or semi-continuous accumulation, but Kuldara contains multiple hiatuses as revealed by the published feldspar $pIRIR_{200,290}$ ages (Fig. 4, Fig. S3; [1]). This shows that the intervals in between the hiatuses of the sequence agree with a mean accumulation rate of ~0.12 m/ka.

Inspection of the luminescence ages indicates that accumulation rate variations approximately occur on a depth scale of 2 m with a variance of ~100 ka^2/m^2, leading us to apply a prior R and variance of Θ^{-1} of 2 meters and 100 ka^2/m^2, respectively, for the upper parts of the sections (Fig. 4 and Fig. S3).

For the lower sections, we increase the prior R to 10 meters. This adjustment is partly done to decrease the simulation time, but also because the cosmogenic nuclide data, which constrain the ages of these deeper layers, cannot resolve accumulation rate variations at a 2-m depth resolution. As the sample spacing for these samples is on the order of ~10 m, it is not possible to resolve accumulation rate changes occurring over 2 m.

The combination of the prior R and variance Θ^{-1} determines the prior uncertainty in age as a function of depth[34]. Hence this prior uncertainty is indirectly determined by the OSL ages and is maintained through the section. Since increasing R inherently increases the prior age uncertainty with depth, we compensate by reducing the prior variance of Θ^{-1} from 100 to 50 ka^2/m^2.

The prior variances associated with the inverse accumulation rate (Θ^{-1}) and correlation ranges (R) are based on the luminescence-derived ages (Table S2).

Supplementary Note 4.1.2: Hiatuses

Four hiatuses have been identified in the Kuldara loess profile based on the feldspar $pIRIR_{200,290}$ ages [1]. A prominent hiatus at ~19.8 m lasted ~100 ka and corresponds to a well-defined erosional boundary observed in the field. Two younger hiatuses with shorter durations have also been identified, whereas the duration of the oldest hiatus at ~19.8 m remains unknown because the $pIRIR_{200,290}$ data obtained from the underlying loess unit (L4) approach saturation indication that the layer is older than ~250 ka. These latter three hiatuses are not discernible in the field.

Two hiatuses have been identified in the profile at Khonako-II. The first, at a depth of ~1.35 m, lasted ~10 ka based on luminescence ages [2]. The second, at a depth of ~119.7 m, was not observed in the field, but further corroborated through lithological, magnetostratigraphic, and rock-magnetic comparisons[4]. This second hiatus is located within the Jaramillo subchron. The Jaramillo subchron has a duration of about 70 thousand years, covering three warm (marine isotope stages (MIS) 28b, 29, and 31) and two cold periods (MIS 29c, 30)[39]. At the Khonako-II profile, the Jaramillo subchrone contains only two pedocomplexes (corresponding to warm periods) and one loess unit (cold period). Using magnetic susceptibility, correlation of the Khonako-II sequence with the more complete sites of the region, at Tagijar and Darai Kalon, reveals that L 15, PC 15 and the upper part of L 16 are missing at Khonako-II, presumably because of erosion; the duration of resulting hiatus is estimated at ~40 kyr[4].

The depths of these hiatuses are incorporated into the CosmoChron model, with prior hiatus duration ranges that include the expected durations (Table S2) based on the descriptions above.

Supplementary Note 4.1.3: Pre-burial cosmogenic nuclide production

We assume that the archaeological artifacts were brought to the surface by river erosion and subsequently collected by early humans and left behind at the sites. Based on this scenario, we use a range of different river erosion rates in the prior model (5–500 m/Myr), while also allowing for episodic erosional events that instantly remove 0–5 m of the sediment. This complex pre-burial model [34] reflects a situation in which the pebbles experienced steady erosion and some degree of abrupt erosion before they were exposed at the surface, affecting the cosmic ray exposure history of the samples and in the end the ^{26}Al-^{10}Be concentrations of the samples.

Furthermore, we assume that the samples could have originated from elevations between 1200 and 2000 m, corresponding to the elevation range stretching from the Obi-Mazar River to the top of the Khovaling loess deposits. Both the elevation and the geographic location define the cosmogenic nuclides production rate and hence influence the ^{26}Al -^{10}Be concentrations of the samples. We used a loess density of 2 g/cm³ and the geographic location of the Khovaling Loess Plateau (38°18'08.90" N, 69°53'29.73" E).

We use a conservative prior pre-burial model to represent a range of pre-burial scenarios; some of these might not be described above but are still represented in the model. As these pre-burial conditions influence the ^{26}Al -^{10}Be concentrations measured in the samples, we obtain a wide range pre-burial ^{26}Al/^{10}Be ratios ranging from 6.2 to 7 (Fig. S2).

Importantly, the mean prior pre-burial ratio is ~6.8, which aligns with the surface samples collected at the field sites (Supplementary Data 4: 6.7 for LA293, 6.8 for OM67, 6.8 for KulPC11.1, and 6.8 for KulPC11.2).

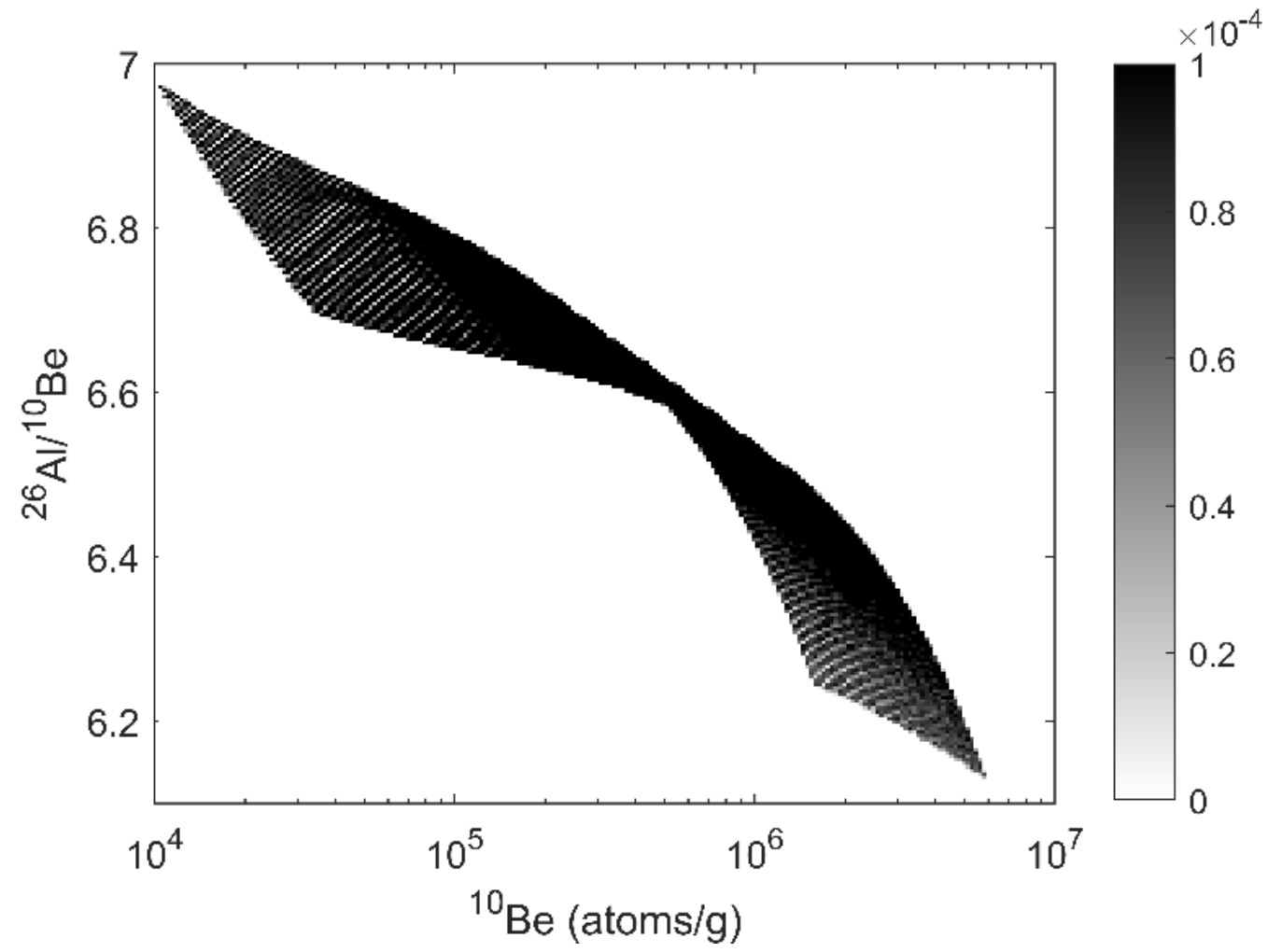

Figure S2: Probability density distribution of pre-burial ^{26}Al-^{10}Be, generated using P-PINI [40,41]. The model uses erosion rates of 5–500 m/Myr at elevations of 1200–2000 m, a rock density of 2 g/cm³, and river erosional event depth of 0–5 m, at the location of the Lakhuti site (38°18'08.90" N, 69°53'29.73" E).

Table S2: The prior constraints on the CosmoChron model parameters (the prior model) used to construct the age-depth models shown in figures 4 and S3. The parameters include Θ^{-1} (inverse accumulation rate), R (correlation range), x_0 (upper age), and T_0 (age at x_0). Pre-burial parameters (*P) are a distribution of ^{26}Al-^{10}Be concentration (Fig. S2) as detailed in Supplementary Note 4.1.3. Different prior variances of Θ^{-1} and R were applied to the upper and lower profile sections, denoted as 'interval' in the table. The elevation, latitude and longitude of the sites and have been used to calculate the ^{10}Be and ^{26}Al production rates and post-burial production at the site.

Location	**Obi-Mazar**		**Kuldara**		**Khonako-II**	
Figure	4, S3		4, S3		4, S3	
Interval (m)	0.1–33	33-84	0.1–20	20–75	0.1–40	40–125
Variance of Θ^{-1} (ka^2/m^2)	100	50	100	50	100	50
R (m)	2	10	2	10	2	10
Mean of Θ^{-1} (ka/m)	8		8		8	
x_0 (m)	0.1		0.1		0.1	
T_0 (ka)	0–40		0-40		0-40	
P	*Complex		*Complex		*Complex	
Hiatus depths (m)			11.5, 17, 17.8, 19.8		1.35	119.7
Hiatus duration (ka)			0–150		0–30	0-60
Elevation (depth=0; m)	1300		1300		1880	
Latitude (°)	38.302472		38.282769		38.359272	
Longitude (°)	69.891592		69.885639		70.046386	

Supplementary Note 4.1.4: Magnetostratigraphic age constraints and the lock-in process

A well-documented discrepancy exists between the positions of geomagnetic polarity boundaries in loess deposits and those in marine sediments. The prevailing explanation attributes this offset to post-depositional remanent magnetization related to the dominant pedogenic processes in loess, which delay the acquisition of a stable magnetic signal in the loess and paleosol units. This process results in a lock-in depth which has been estimated to 0–3 m in Chinese loess (e.g. Pan et al., (2021)). Consequently, the observed magnetostratigraphic boundaries may appear shifted downward relative to their actual depositional depth. This general trend, which is observed in many loess deposits across the world, cannot be attributed to changes in the magnetic minerals carrying the magnetic remanence.

To account for this uncertainty, we incorporate the depth of geomagnetic polarity boundaries as a free parameter in the model, allowing for potential shifts ranging from 0 to 3 meters. In other words, we allow the lock-in depth to take on any value between 0 and 3 m below the observed depth in the age-depth models.

The specific depths of the polarity changes observed in the loess profiles as well as the associated reference ages presented in figures 4 and S3 are listed in Table S3.

Table S3. Depths of the paleomagnetic boundaries and the reference ages applied in the models.

	Kuldara Depth (m)	Khonako-II Depth (m)	Age (Ma)
Ending of the Matuyama-Brunhes transition	74.96 [3]	95.52 [4]	0.77 [43]
Beginning of the Matuyama-Brunhes transition	77.86 [3]	98.20 [4]	0.80 [43]
Upper Jaramillo		118.97 [4]	0.99 [44]
Lower Jaramillo		121.6 [4]	1.07 [44]

Supplementary Note 4.2: Individual age-depth models for the three loess profiles and sensitivity of different data

We first constructed CosmoChron age-depth models for each individual site without incorporating transposed ages (Fig. S3), by integrating luminescence ages, magnetostratigraphic boundaries, and measured cosmogenic ^{26}Al-^{10}Be concentrations from each site alone. These models incorporate the prior model constraints described above, including accumulation rate processes (Supplementary Note 4.1.1), hiatus durations (Supplementary Note 4.1.2), pre-burial ^{26}Al and ^{10}Be production (Supplementary Note 4.1.3), and magnetostratigraphic lock-in depths (Supplementary Note 4.1.4). We then assessed the sensitivity of the resulting models to the inclusion of different datasets.

For models integrating luminescence ages, magnetostratigraphic boundaries, and cosmogenic nuclide concentrations, the age at the middle depth of PC4 has a 1σ uncertainty of ~ 52 ka for both Khonako-II and Kuldara (Fig. S2: E, K and Q). In contrast, the uncertainties at PC5 and PC6 are larger (~ 75 ka) reflecting the smaller number of ^{26}Al-^{10}Be samples at these positions, all originating from PC4 in these profiles. As a result, ages at PC4 are better constrained than at PC5 and PC6. At Obi-Mazar, the age-depth model exhibits smaller uncertainties for PC4, PC5, and PC6 due to a greater number of cosmogenic samples available at this site (one sample from PC4, eight from PC5, and two from PC6; see Table S2). For example, the 1σ uncertainty for the age at the middle of PC5 at Obi-Mazar is ~ 50 ka, ~ 25 ka lower than the corresponding uncertainties for Kuldara and Khonako-II.

Importantly, PCs 4, 5, and 6 most likely accumulated during Marine Isotope Stages 11, 13, and 15, respectively, across all three sites (colored error bars in panels U), based on the age-depth model integrating luminescence ages, magnetostratigraphic boundaries, and cosmogenic nuclide concentrations shown in figure S3 panels E, K and Q. This provides a prerequisite for integrating transposed ages, as discussed in the main text.

Supplementary Note 4.1: Sensitivity of age-depth models to different datasets

We tested the sensitivity of the age-depth models to different datasets. When constructing models using only luminescence ages and magnetostratigraphic boundaries (excluding ^{26}Al

and ^{10}Be concentrations), the age uncertainties in the PC4–6 depth range generally increase and from ~10–150 % depending on the site and the pedocomplex (Fig. S3 C, I, O, Table S4), compared to the age-depth models that includes all the data (Fig. S3 E, K, Q, Table S4). This is because the cosmogenic nuclide data, which contribute important age constraints for these layers are omitted. The effect is particularly pronounced at Obi-Mazar (Fig. S3C), where the lower part of the age-depth model (>40 m) is unconstrained by magnetostratigraphic boundaries, in contrast to the Kuldara and Khonako-II models (Fig. S3 I and O).

Conversely, when constructing models using only ^{26}Al and ^{10}Be concentrations (excluding luminescence dating and magnetostratigraphy), the uncertainties in the upper ~250 ka of the profiles increase up to ~1000% (age-depth model shown in Fig. S3 D, J, P relative to those shown in Fig. S3 C, I, O), as these layers are primarily constrained by luminescence ages in the full model. However, the lower portions of the profiles, particularly around PC4, remain visually constrained by the cosmogenic nuclide data at Kuldara and Khonako-II. At Kuldara, the age-depth model 'bends' to accommodate the ^{26}Al-^{10}Be constraints, while at Khonako-II, uncertainties narrow around the depth of PC4. Below PC4, the models for Kuldara and Khonako-II are less well-constrained due to the absence of magnetostratigraphic boundaries. In contrast, the age-depth model for Obi-Mazar shows similar uncertainties around PC5 and PC6 when using only cosmogenic nuclide data (Fig. S3P) compared to the full model (Fig. S3E). This is because these layers are predominantly constrained by the ^{26}Al and ^{10}Be measurements at Obi-Mazar from these layers.

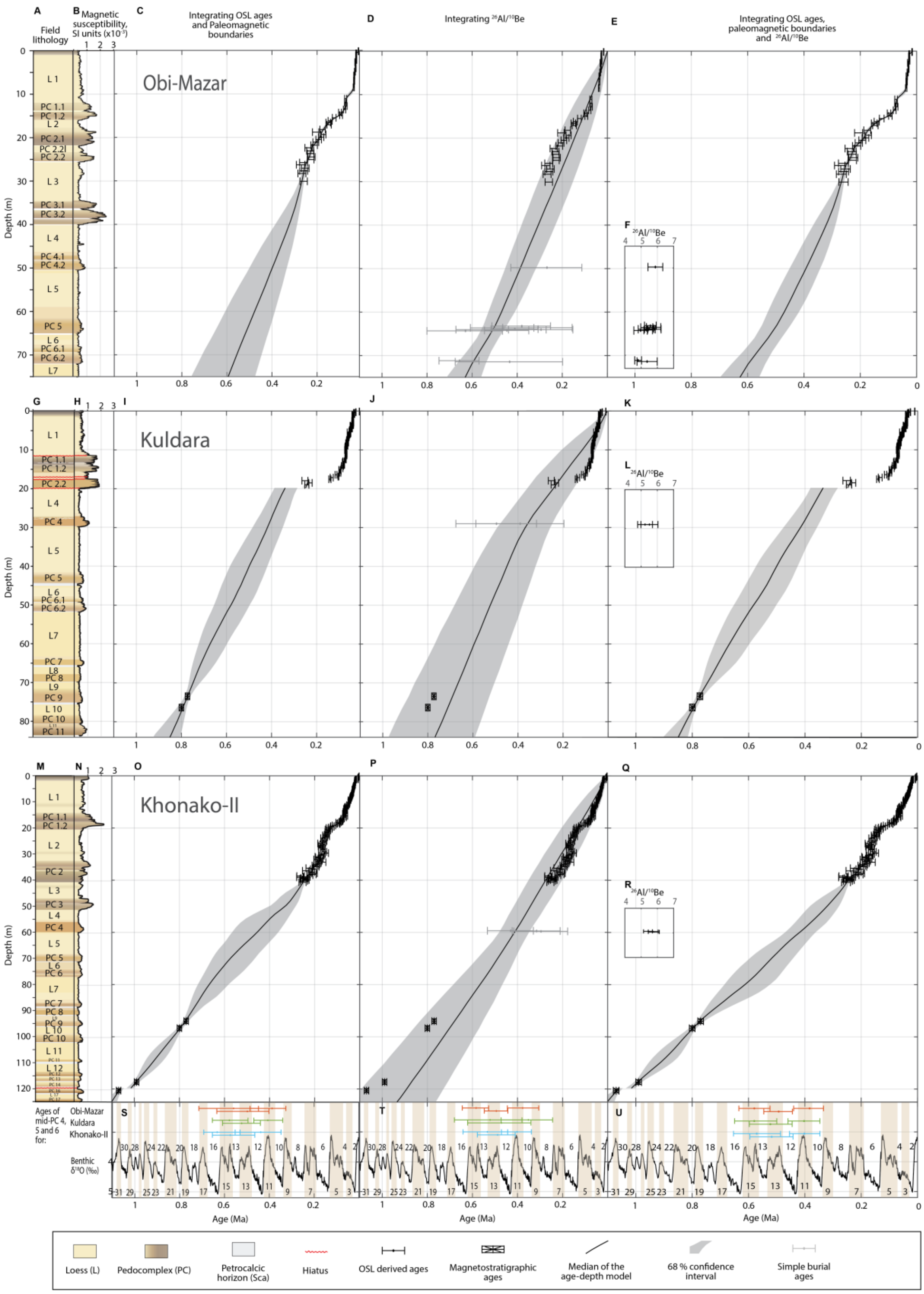

Figure S3: Age-depth models for the Khovaling loess deposits, Tajikistan, using different data. **A**, **G**, and **M**: field lithology for Obi-Mazar, Kuldara and Khonako-II, respectively. **B**, **H**, and **N**: magnetic susceptibility for Obi-Mazar (n = 2317), Kuldara (n = 2861) and Khonako-II (n = 1038), respectively. **C**, **I**, and **O**: CosmoChron age-depth models for Obi-Mazar, Kuldara and Khonako-II, respectively, based on luminescence ages and magnetostratigraphic boundaries (excluding ^{26}Al and ^{10}Be

concentrations). N = 40, 78 and 168 luminesense ages, n = 0, 2 and 4 magnetostratigraphic boundaries for Obi-Mazar, Kuldara and Khonako-II, respectively. **D**, **J**, and **P**: CosmoChron age-depth models for Obi-Mazar, Kuldara and Khonako-II, respectively, based on cosmogenic ^{26}Al and ^{10}Be concentration (excluding luminescence dating and magnetostratigraphy). n = 11, 2 and 3 $^{26}Al/^{10}Be$ ratios for Obi-Mazar, Kuldara and Khonako-II, respectively. **E**, **K**, and **Q**: CosmoChron age-depth models for Obi-Mazar, Kuldara and Khonako-II, respectively, using cosmogenic ^{26}Al and ^{10}Be concentrations, luminescence ages and magnetostratigraphic boundaries. n = 40, 78 and 168 luminesense ages, n = 0, 2 and 4 magnetostratigraphic boundaries, and n = 11, 2 and 3 $^{26}Al/^{10}Be$ ratios for Obi-Mazar, Kuldara and Khonako-II, respectively. For panels C–E, I–K and O–Q: Black dots and horizontal error bars represent the individual luminescence ages or magnetostratigraphic boundaries and their corresponding 1σ age uncertainties, respectively. The vertical error bars on the magnetostratigraphic boundaries represent the standard deviation (1σ) of the posterior lock-in depth (see Figure S9). **F**, **L**, and **R**: measured $^{26}Al/^{10}Be$ ratios and the associated 1σ uncertainties for Obi-Mazar (n = 11), Kuldara (n = 2) and Khonako-II (n = 3), respectively (see Supplementary Data 4). **S**, **T** and **U**: The LR04 $\delta^{18}O$ stack (Lisiecki & Raymo, 2005) shown as a black curve. The numbered shaded areas correspond to marine isotope stages (MIS). Dots and corresponding error bars indicate median and 1σ age uncertainties, respectively, of the the mid-age for PC 4, 5, and 6 at Obi-Mazar (red), Kuldara (green), and Khonako-II (blue) (see Table S2).

At Khonako-II, three ^{26}Al-^{10}Be samples provide strong age control for PC 4. However, at Kuldara, the age of PC 4 also depends on the duration of the hiatus above, which currently has a maximum limit of 150 ka in the model. Expanding this range beyond 150 ka would imply that MIS 11 is absent at Kuldara, which, in turn, would complicate the correlation between PCs and marine isotope stages between MIS 13 and the Matuyama-Brunhes boundary. Furthermore, the age-depth model solely based on ^{26}Al-^{10}Be concentrations at Kuldara also indicates that PC 4 most likely corresponds to MIS 11 supporting the current age-depth model and the maximum hiatus duration of 150 ka (Fig. S3; Table S4)

Table S4: The age at the middle depth of pedo-complexes (PCs) determined by including different data in the CosmoChron method. In the table 'cosmo' is short for including for ^{26}Al-^{10}Be concentration in the CosmoChron method, 'Lum' is short for including luminescence ages, and 'paleomag' for including magnetostratigraphic boundaries.

Site	Layer	Depth (m)	Age using: Lum + paleomag (ka)	Age using: Cosmo (ka)	Age using: Cosmo + Lum + paleomag (ka)	Age using: Cosmo + Lum + paleomag + transposed ages (ka)
Obi-Mazar	PC 4	48.75	389 +98/-60	377 +68/-71	384 +57/-49	400 +31/-29
	PC 5	62.71	499 +135/-96	494 +53/-51	494 +53/-49	508 +34/-33
	PC 6	70.44	560 +154/-111	584 +61/-55	581 +55/-56	582 +40/35
Kuldara	PC 4	28.55	407 +62/-66	352 +121/-108	404 +57/-56	405 +28/-30
	PC 5	43.15	534 +85/-83	471 +150/-131	524 +75/-77	519 +34/-34
	PC 6	49.8	579 +75/-83	522 +158/-142	581 +70/-80	581 +37/-35
Khonako-II	PC 4	58.5	439 +114/-90	402 +67/-64	403 +53/-55	389 +29/-28
	PC 5	70.0	574 +79/-109	488 +90/-78	519 +78/-76	514 +32/-35
	PC 6	76.0	633 +60/-102	534 +108/-90	572 +83/-85	581 +37/-37

Supplementary Note 4.3: Probabilistic Dynamic Time Warping alignment models

The magnetic susceptibility curves were aligned using the Probabilistic Dynamic Time Warping (P-DTW) algorithm[35]. This algorithm automatically aligns signals while estimating the associated alignment uncertainty. Once the alignment models have been constructed, they allow for the transposition of ages across sites, including the associated uncertainties.

The P-DTW parameter settings were optimized through trial and error until the models appear physical realistic and compatible with the individually age-depth models. The model parameter p controls the influence of the signals on the alignment model ($p = 1$ corresponds to the classic deterministic DTW algorithm, while $p = -\infty$ constructs an alignment model independent of the signals), while the parameters *ms*, *ml* and *ss* define path-moment strength, path-moment length and steer strength, respectively (see Sørensen et al.[35] for details). The settings are listed in Table S5.

The signals were pre-processed before alignment as listed for each alignment model in Table S5. The constant values 10 and 1 were added to avoid negative values, while logarithmic scaling was applied to reduce the influence of large relative amplitude variations. Each signal was linearly interpolated in depth to a length of 3000 data points.

Hiatuses identified at Kuldara were incorporated into the models based on the independent age-depth model (red lines) (Fig. S3, panels E, K and Q). Additionally, the alignment model between Khonako-II and Kuldara was further constrained using the Matuyama-Brunhes paleomagnetic boundary (Table S3), with these constraints marked by blue stars in figure 3D and 3H.

As shown in figure S4, the alignment models largely agree with the independent age-depth models (Fig. S3, panels E, K and Q) for all three sites, reinforcing confidence in the accuracy of the individual models.

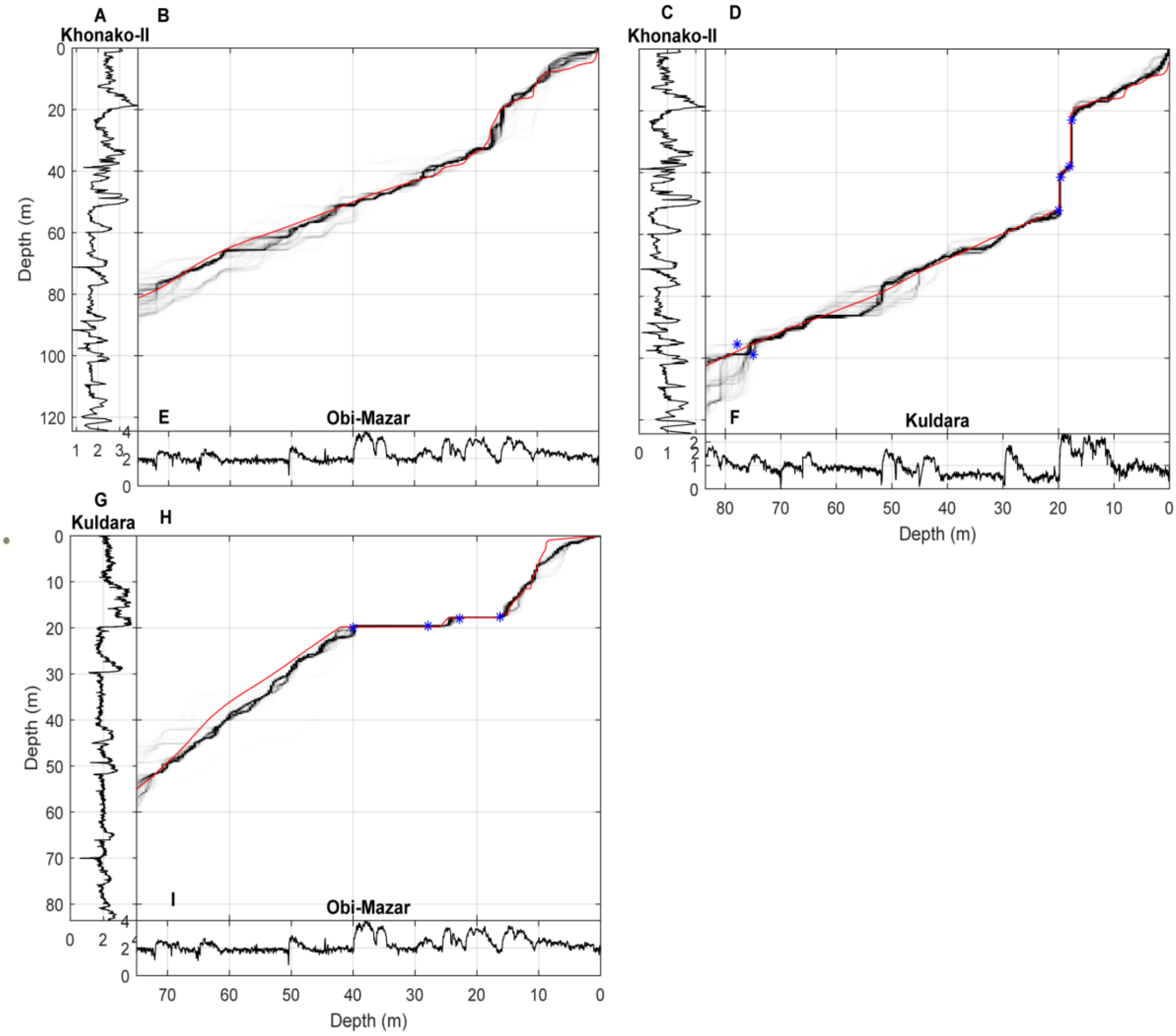

Figure S4: P-DTW alignment models used to transpose ages from the independent age-depth models (Fig. S3, Panels E, K and Q) across sites, as applied in the age-depth models shown in figure 4. **A**, **C**, **E**, **F**, **G** and **I**: processed magnetic susceptibility curves for each site (see Table S5; Obi-Mazar (n=2317), Kuldara (n=2861) and Khonako-II (n=1038)). **B**: alignment model between Khonako-II and Obi-Mazar, **D**: alignment model between Khonako-II and Kuldara, **H**: alignment model between Kuldara and Obi-Mazar. For both **B**, **D** and **H**: grayscale represents the P-DTW alignment model, blue stars indicate tie points, red curves are the median of the independent age-depth models (Fig. S3) plotted against the sites.

Table S5: P-DTW-settings used to generate the alignment models shown in figure S4 which are subsequently applied to transpose the ages shown in Figure 4. *a1* denotes the original magnetic susceptibility signals, while *a* denotes the processed signals. 'detrend' is a function that removes the best straight-fit line from the signal.

	Procession of signals	Initial depth (m)	p	ms	ml	ss
Obi-Mazar vs Kuldara	a = log(a1)+10	45–55 at Kuldara	0.8	1	30	0.25
Obi-Mazar vs Khonako-II	a = log(a1)+10	70–90 at Khonako-II	0.5	1	15	0.25
Kuldara vs Khonako-II	a =detrend(log(a1)+10)+1	97–120 at Khonako-II	0.7	1	30	0.25

Supplementary Note 4.4: Transposed ages

Age constraints from the individual age-depth models (Fig. S3, Panels E, K and Q) are transposed across sites in 10-m intervals below the segments with OSL ages using the alignment models shown in figure S4. We use 10-m intervals because the age uncertainties result in overlapping uncertainties between the transposed ages given the mean accumulation rate in the age-depth models (Fig. S3, Panels E, K and Q). Hence, including additional transposed ages would not further enhance the age-depth models.

We do not include transposed ages for the upper sections covered by luminescence dating. For both Obi-Mazar, Kuldara and Khonako-II, the upper sections are well-constrained by luminescence ages, and there is nothing to be gained by transposing ages in the luminescence dating segments. The ages within the upper ~250 ka are too well-defined compared to the uncertainty of the alignment. We also note that the alignment models (Fig. S4) agree with the independent age-depth models for all three sites for the stratigraphic intervals constraint by the luminescence ages.

The transposed ages are incorporated into CosmoChron's probabilistic inverse modeling framework by treating the depths of the ages as free parameters. We use the depth-depth relationships from the P-DTW alignment models (Fig. S4-7) as prior distributions for these depths. They can be multimodal or have any other probability distribution. To draw samples from the prior depth distribution, we apply an inverse normal score transformation[45], implemented through the SIPPI toolbox for MATLAB[38].

Figures S5–S7 show the prior and posterior distributions of the transposed depths related to the age-depth models in figure 4. The prior distributions are represented by 1D histograms of the 2D alignment models in Fig. S4 at the specified depths denoted above each panel. For all the transposed depths (Fig. S5–S7), the prior and posterior distributions are nearly identical. This reflects that it is the alignment models (Figure S4) that constrain the age-depth models (Figure S3 vs. 4), rather than the reverse. However, as shown in Sørensen et al.[35], merging age constraints across sites using the P-DTW algorithm can in some cases improve both the alignment and the age-depth models.

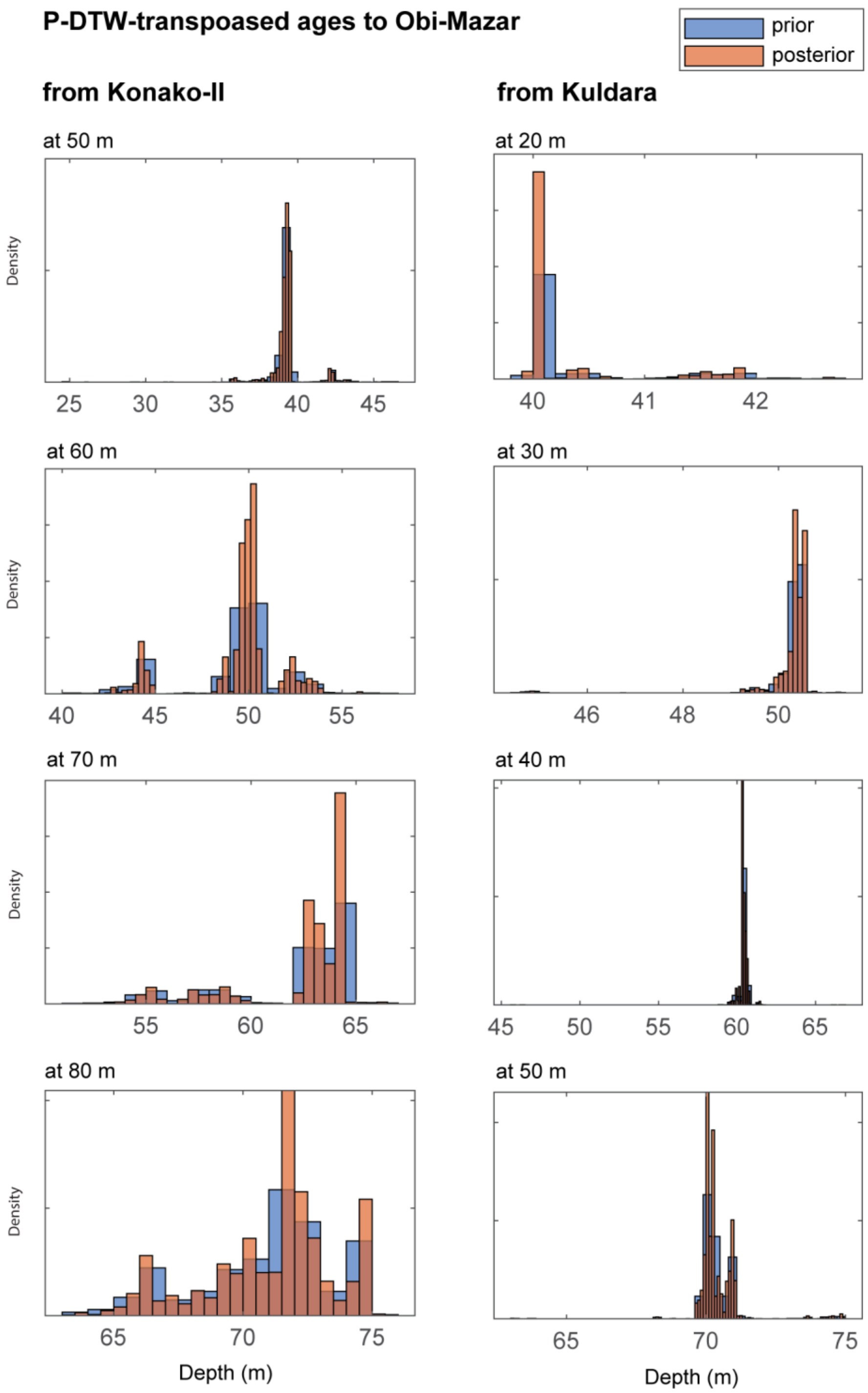

Figure S5: Prior and posterior transposed depths for the age-depth model for Obi-Mazar shown in figure 4

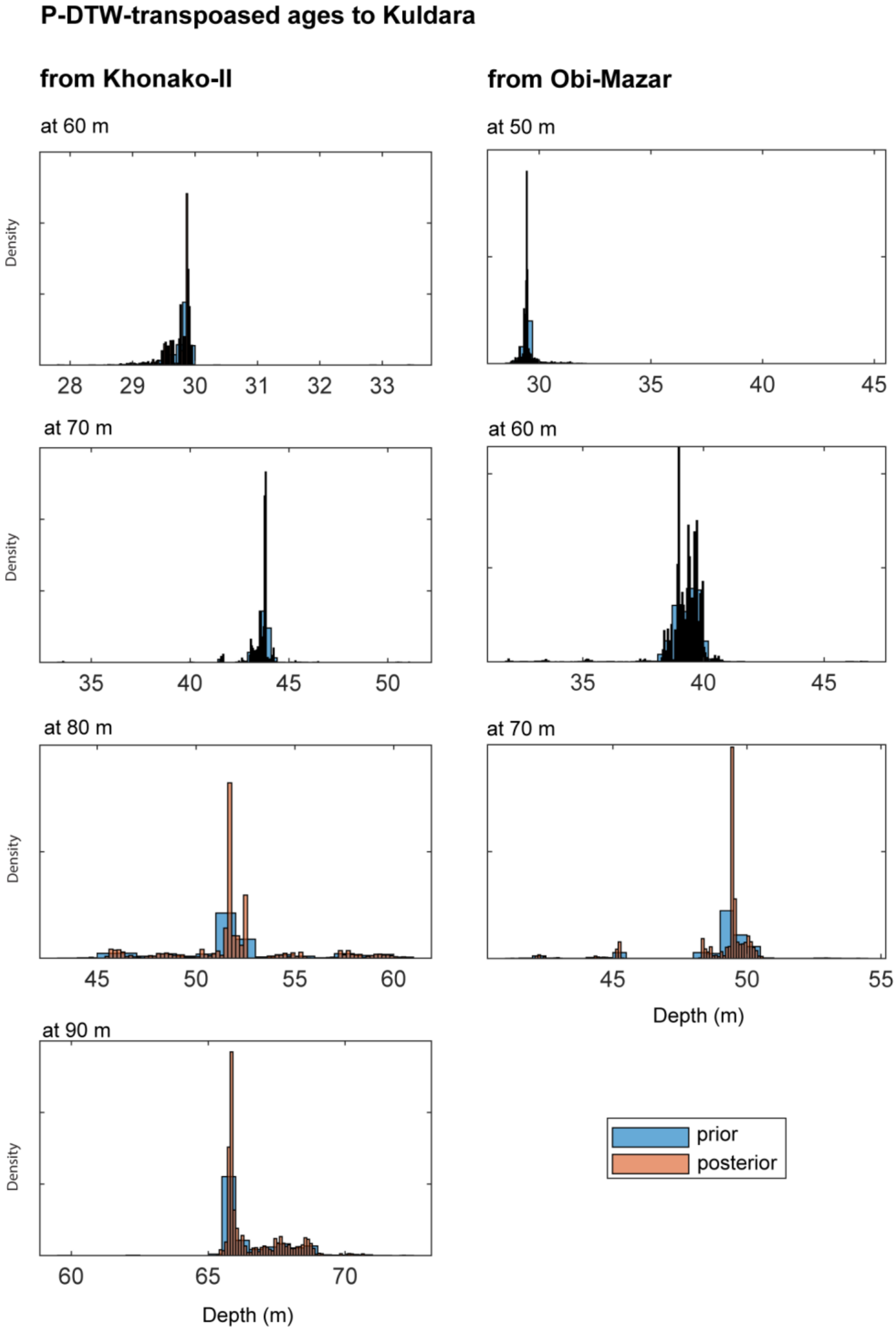

Figure S6: Prior and posterior transposed depths for the age-depth model for Kuldara shown in figure 4.

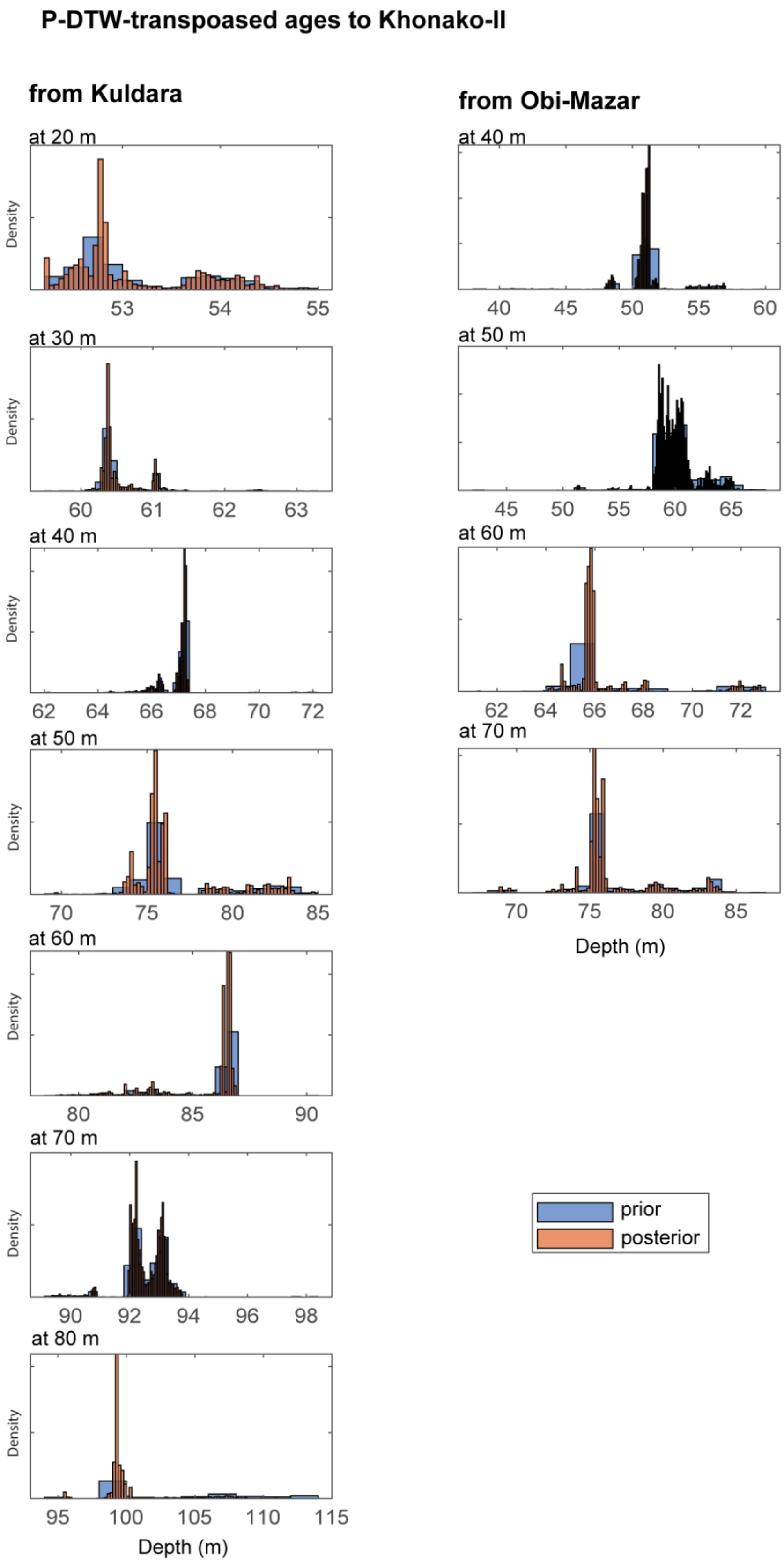

Figure S7: Prior and posterior transposed depths for the age-depth model for Khonako-II shown in figure 4.

Supplementary Note 4.5: Posterior hiatus durations

The depths and prior duration constraints for each hiatus incorporated into the CosmoChron age-depth models are detailed in Supplementary Note 4.1.2 and listed in Table S2. Here, we present the posterior distributions of hiatus durations associated with the age-depth models shown in Figure 4 of the main text.

Hiatus durations constrained by OSL ages exhibit approximately Gaussian posterior distributions with relatively low uncertainties. These include the hiatuses at 17.0 m and 17.8 m depth at Kuldara, and at 1.35 m depth at Khonako-II. In contrast, the hiatus at 19.8 m depth at Kuldara, constrained by cosmogenic ^{26}Al and ^{10}Be concentrations from sample PC4, yields a broader, non-Gaussian posterior distribution, peaking at ~ 110 ka and reflecting a higher degree of uncertainty.

The hiatus within the Jaramillo subchron at 119.7 m depth at Khonako-II displays a posterior distribution skewed toward longer durations within the prior range. This tendency likely results from the influence of the assumed prior mean accumulation rate (0.12 m/ka), which here favors longer hiatus durations.

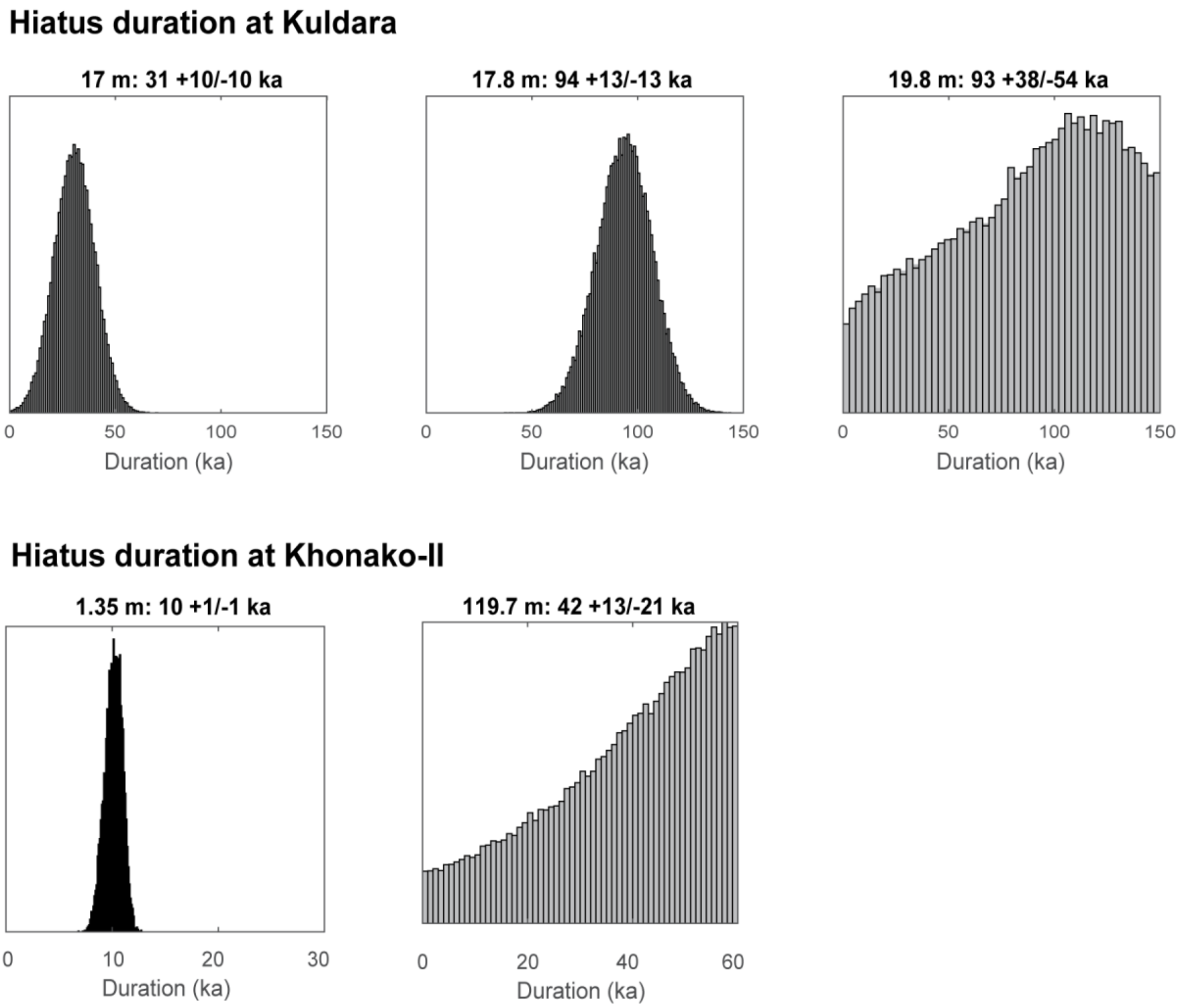

Figure S8: Posterior hiatus durations for the age-depth models shown in figure 4. The age range of each panel reflects the prior durations (Table S2).

Supplementary Note 4.6: Posterior paleomagnetic lock-in depths

The posterior lock-in depths exhibit only slight deviations from the prior for the Matuyama-Brunhes and Upper Jaramillo boundaries (Fig. S9), with both having a mean posterior lock-in depth of ~1.5 m below the observed depth. The skewed distributions are primarily induced by the transposed ages near the depths of these boundaries as shown in figure 4. Notably, the posterior lock-in depth distribution for the lower Jaramillo significantly decreases at depths greater than 2 m. This pattern is due to the hiatus incorporated at 119.7 m, which lies ~2 m above the paleomagnetic boundary at 121.6 m. This indicates that it is highly unlikely for the true lower Jaramillo boundary to be located above the depth of this hiatus.

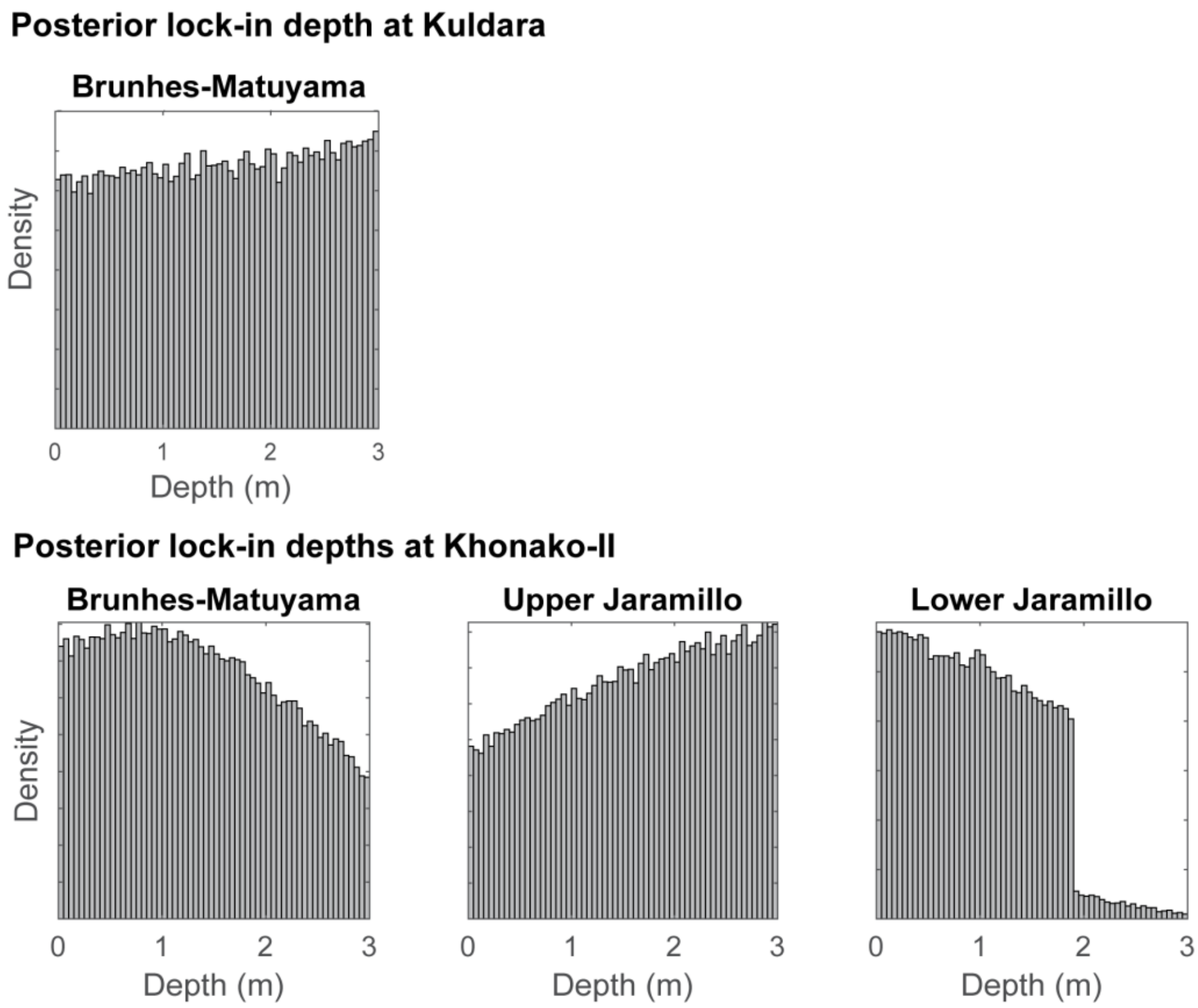

Figure S9: Posterior paleomagnetic lock-in depths for the age-depth models shown in figure 4. The depth range of each panel reflects the prior lock-in depth.

Supplementary Note 5: The Early Paleolithic Karatau Culture

Supplementary Note 5.1: Historiographical contexts of the Karatau Culture

Situated strategically between the western and eastern regions of the Eurasian continent, Central Asia served as a crucial migration corridor for hominins during the Pleistocene.. Notably, modern-day Tajikistan has been recognized as a focal point of Early Paleolithic activity in this region, most likely due to the fact that perennial water sources are common, whereas in other parts of the region, there are few. This has created a situation in which there are only isolated stratified sites or surface artifact scatters in other parts of Central Asia, however Tajikistan features a dense concentration of Early Paleolithic sites, many of which are synchronous with one another.

Early research on the Lower Paleolithic in Tajikistan is closely linked to the work of the prominent Soviet archaeologist V.A. Ranov. In 1973, Ranov established a collaborative partnership with geologist A.A. Lazarenko to investigate the Karatau loess-paleosol sequence in the middle reaches of the Vakhsh River[46,47]. The initial focus of the investigation was to establish a formation model for the deposition of sediments and formation of soils, which were labeled sequentially with Pedocomplex (PC) 0 beginning in the Holocene and subsequent PCs forming during interstadial events going back 1+ Ma. As a result of extensive research at the sites in the Vakhsh River Valley, substantial assemblages of archaeological material were recovered from PC 6. These findings prompted V.A. Ranov to define the first Lower Paleolithic culture in Central Asia, which he designated as the Karatau culture[48]. The Karatau site was excavated only within PC 6; no artifacts were found in the upper part of the section, while the lower part remained unexcavated due to the hardness of the sediments[46]. In the following years between 1980 and into the 2000s, the majority of the sites of the Karatau culture were discovered and studied by Ranov in the valley of the Obi-Mazar River (Pyanj River basin), which include Lakhuti-I, Obi-Mazar-IV and -VI, and Khonako-III[23,29,46,47,49–53]. The Lakhuti-IV site was discovered in 2021[30].

Ranov conceptualized the Karatau culture as a flake-based technocomplex characterized by pebble-core technology, predominantly featuring citron and flat-faced cores[50].

The ‘citron flaking’ technique is characterized by the detachment of flakes from the transversal plane of large pebbles. The resulting ‘citron flakes’ are diagnostically defined by the preservation of cortical surfaces on their proximal, lateral, or distal margins. Technologically, this approach parallels knapping on slab cores[54] and shares conceptual ground with core exploitation strategies in Quina technology[55].

The lithic assemblages from this technocomplex exhibit minimal standardization and serial production, with flakes demonstrating considerable morphometric variability. Nevertheless, a consistent toolkit recurs across all assemblages, comprising choppers, side-scrapers, atypical end-scrapers, and denticulated tools. In PC 4, Ranov and Schäfer[52] identified "certain technical features that bring it closer to Middle Paleolithic industries." According to Ranov[50], these transitional characteristics primarily manifest in the types of

reduction strategies employed, particularly through the presence of intensively worked small-sized cores.

The Karatau culture is comprised of the following sites, each with one to three industries: Obi-Mazar-VI (PC 6), Karatau (PC 6), Karamaydan (PC 6), Lahuti-IV (PC 6-4), Lakhuti-I (PC 5), Obi-Mazar-IV (PC 4), Khonako-III (PC 4). The total collection of lithic artifacts amounts to approximately 6500 specimens, which were primarily buried in paleosols of PC 6 (MIS 15), PC 5 (MIS 13) and PC 4 (MIS 11), with occasional artifacts found in unweathered loess sediments. In the paleosols underlying (PC 7-9) and overlying (PC 3), less than 10 artifacts were recorded in total across all studied sections. Considering the studied areas and the possibility of vertical movement of these items as a result of bioturbation, it is possible to consider these deposits as archaeologically "sterile". Nevertheless, the sporadic presence of artefacts in some loess units and some pedocomplexes (PC3, PC7, PC8, and PC9), may also indicate that small groups of hominins migrated through the area during these periods.

The historiography of research into the Karatau culture can be summarized as follows[50]:

- Karatau site was studied for seven seasons between 1973–1986. Archaeological material, numbering 1295 artifacts, was found in PC 6. The total area of excavations was 500 m^2.
- Lakhuti-I was excavated for four seasons from 1974–1980. Archaeological material, numbering 1208 artifacts, was found in PC 5. The total area of excavation was about 100 m^2.
- Obi-Mazar-IV was studied for five seasons between 1983–1998. Archaeological material, numbering 1722 artifacts, was found in PC 4. The total area of excavations was 30 m^2.
- Karamaydan was studied for one season in 1984. The archaeological material, numbering about 50 artifacts, was recovered in PC 6. The total excavation area was 50 m^2
- Khonako-III was investigated for three seasons in 1995–2000. Archaeological material, numbering about 200 artifacts, was found in PC 4. The total excavated area was 40 m^2.
- Obi-Mazar-VI was excavated for three successive seasons from 1996-1998. Archaeological material, numbering about 150 artifacts, was found in PC 6. The total area of excavations was 60 m^2.
- Lakhuti-IV was discovered after Ranov's death and was investigated for three seasons between 2021–2023. The archaeological material recovered from PC 6–4 comprises 2600 artifacts. The total excavated area was 55 m^2.

Supplementary Note 5.2: The *chaîne opératoire* analysis of the most preserved Karatau assemblages

Given that the majority of the artifact assemblages were excavated during the 20th century and are presently physically divided in Tajikistan between a scientific center and several museums, it is now almost impossible to account for, much less study, the complete

collection. In our current study, the focus is directed towards assemblages that have demonstrated the highest degree of preservation, as evidenced by Ranov's field documentation.

Supplementary Note 5.2.1: The Karatau site

Cores and core-like chunks account for 6.0% of the assemblage, excluding waste products. The use of radial, citron and flat-faced unidirectional cores is documented. Flat-faced unidirectional cores account for 63.0% of all cores. The next largest group is exhausted cores (21.0%). Radial and citron cores are represented by single specimens. Preliminary preparation or removals of technical spalls on all types of cores are absent or minimally performed (one or two spalls). Pebble cortex or flat surfaces were used as striking platforms. Core fronts were not prepared; similarly, no technical spalls on the cores' striking platforms were recorded.

Waste products and small flakes (<1.5 cm in maximum dimension) form a significant part of the assemblage (52.7%). Blades are absent. Among the flakes, citron flakes of various sizes account for 23.0% of the assemblage, while the number of citron cores is insignificant. Among the flakes, the proportion of cortical flakes (representing the initial stage of flaking) is 13.1%, with pebble cortex present on 40.6% of all the flakes. In terms of size, flakes are dominated by large flakes - 56.6%, which may possibly be related to the incompleteness of the collection. The striking platforms are typically cortical (54.9%) or flat (44.7%), with the rest being dihedral. There is one hammerstone in the collection, in the form of a medium-elongated pebble, with traces of hammering on one of the ends.

Within the toolkit (5.1% of the collection, excluding waste products), the most numerous and diverse categories are single scrapers on large flakes or split pebbles with varying degrees of modification. Choppers, the second most common type of tool, are also well represented. Denticulated tools and unifaces are present in a few samples. An atypical scraper and a massive "nose" tool, close to a pick, are documented. The most typical category of tools are unifaces, which are small plano-convex tools of rounded shapes. The convex surfaces of these tools are pebble cortex, and the flat surfaces are covered with centripetal negatives of various sizes, which look like relatively thin technical flakes for flattening the surface rather than for obtaining targeted flakes using the radial technique.

Supplementary Note 5.2.2: Lakhuti-I site

Archaeological material from this site was recovered from PC 5 with cores and core-like chunks account for 7.1% of the assemblage excluding waste products. The use of radial, citron and flat-faced unidirectional cores is documented. Flat-faced unidirectional cores account for 39.5% of all cores. Overall, radial and citron cores are present in smaller amounts from this assemblage than in assemblages from PC 6, but they occur in approximately equal proportions (~25%) relative to the overall assemblage. There are also a few cores on massive flakes (10.5%).

In general, preliminary preparation or removals of technical spalls on all types of cores is absent or minimally performed (one or two spalls). The removal of small and medium flakes was carried out on the ventral surface of the blank. Pebble cortex or flat

surfaces were used as striking platforms. The core front was generally not prepared and no technical spalls on cores' striking platforms were recorded.

Waste products and small flakes (<1.5 cm in maximum dimension) form a significant part of the assemblage – 37.7%. Blades are absent. Among the flakes, citron flakes of various sizes account for 24.3% of the overall assemblage. The proportion of cortical surface representing the initial stage of flaking is 15.3%, with pebble cortex present on 75.3% of all the flakes (the pebble surface of cortical striking platforms was also included in this count). In terms of size, medium-sized flakes dominate among the flakes - 61.2% with large flakes accounting for 32.4% of the assemblage. The striking platforms are typically cortical (68.4%) or flat (31.7%), with the rest being dihedral.

Within the tool kit (2.7% of the collection, excluding waste products), the most numerous and diverse categories are unifaces, choppers and single scrapers on large flakes or split pebbles with varying degrees of modification. In a few specimens, notched tools are present, with no additional retouching. A total of 29.2% of the flakes have retouch. Unifaces, which make up 25.0% of the assemblage, are in various stages of manufacture. They range in size from 8 to 4 cm along the long axis, while the reduction processes correspond to that described for the Karatau site (see Supplementary Note 5.2.1).

Supplementary Note 5.2.3: Obi-Mazar-IV site

Cores and core-like chunks account for 1.8% of the assemblage, excluding waste products. The use of radial, citron and flat-faced unidirectional cores is documented. Radial cores are the most numerous (50%), with slightly fewer flat-faced unidirectional cores and a single citron core. Pebble cortex or flat surfaces were used as striking platforms. The core fronts were not prepared and no technical spalls on cores' striking platforms were recorded.

Waste products and small flakes (<1.5 cm in maximum dimension) form a significant part of the assemblage – 56.9%. Blades are absent. Among the flakes, citron flakes of various sizes account for 11.3% of the assemblage. The proportion of cortical surface representing the initial stage of flaking is 19.2%, with pebble cortex present on 61.9% of all the flakes (the pebble surface of cortical striking platforms was also included in this count). Medium-sized flakes dominate the assemblage at 71.4%, and large flakes account for 9.5%. The striking platforms are typically cortical (68.4%) or flat (31.7%).

Within the toolkit (0.5% of the collection, excluding waste products), the most numerous are unifaces. A single side-scraper on split pebbles and a single chopper were recovered. The generation of medium sized (4–5 cm) unifaces is similar to that described for the Karatau site (see Supplementary Note 5.2.1).

Table S6: Overview of the lithic assemblages used in this study

	Karatau		Lakhuti-IV		Obi-Mazar-VI	
	PC 6	%	PC 5	%	PC 4	%
Split pebbles	56	4.4	20	3.1	155	9.7
Cores	19	1.5	18	2.7	6	0.4
Core-like chunks	26	2	6	0.9	6	0.4
Flakes	512	40.2	247	37.3	519	32.5
Cortical (75–100%)	*45*	*3.5*	*17*	*2.6*	*68*	*4.3*
With cortical surface (1–74%)	*163*	*12.8*	*16*	*2.4*	*250*	*15.6*
Without cortical surface	*304*	*23.8*	*217*	*32.8*	*201*	*12.6*
Small flakes (≤1,5 cm)	136	10.7	87	13.1	232	14.5
Chunks	367	28.8	172	26.0	407	25.5
Waste products	159	12.5	59	8.9	273	17.1
Chips	0	0	53	8.0	0	0
Total	1275	100	662	100	1598	100

Table S7: Overview of the tool types reported this study

Tool types	Karatau PC 6	Lakhuti-IV PC 5	Obi-Mazar-VI PC 4
Side-scrapers	15	4	1
End-scrapers atypical	1	2	0
Denticulated	3	2	0
Notched with retouch	0	1	0
Notched without retouch	2	2	0
Unifaces	3	4	3
Unidentifiable tools with retouch	0	5	0
Hammerstones	1	8	0
Retouched flakes	3	10	0
Choppers	5	0	1
Nosed tools	1	0	0
Total	34	21	5

Table S8: Overview of the core types reported this study

Core types	Karatau PC 6	Lakhuti-IV PC 5	Obi-Mazar-VI PC 4
Flat faced	12	3	3
Radial	1	6	5
Saucisson	2	2	1
Cores on flake	0	1	0
Exhausted	4	2	2
Total	19	18	11

Supplementary Note 5.3: Technological and typological characteristics of the Karatau culture.

In this study, we have considered all known assemblages of the Karatau culture, including museum samples (Table S6), which allows for a more comprehensive assessment of its characteristics in comparison to the complexes that have been previously analyzed.

Cores account for 2–5% of the assemblages recovered across the various sites. Several techniques were used in primary flaking: radial, citron and flat-faced. Flat-faced techniques are characterized by unidirectional flake removals only. The cores were subjected to minimal or no preparation, exhibiting only one or two spalls. It is evident that only a flat striking platform was created, with no evidence of preparation for the flaking front or laterals. In the assemblages of several sites (Lakhuti-I PC 5, Lakhuti-IV PC 5, etc.), a small group of cores was identified derived from large, massive flakes, where the removal of small and medium-sized flakes was carried out along the ventral surface of the blank. No technical flakes related to the processing of cores other than primary flakes (up to 13% of all flakes) have been recorded in the collections. Pebble surfaces are present on up to ~70% of flakes (the pebble surface of cortical striking platforms was also included in this count).

Waste products, which include small flakes (<1.5 cm) and chips indicative of blank retouching, account for the majority of debitage (up to 70%). Citron is abundant among the flakes (up to 8%). Striking platforms are plain or cortical, with other types being almost non-existent. In the collection of almost all sites there are hammerstones, in the form of elongated pebbles of different sizes and weights, with traces of hammering at one or both ends.

The toolkit, which constitutes up to 5% of assemblages devoid of waste products, contains a significant proportion of retouched flakes and unidentifiable tools (up to 25%). Among the formal tools are choppers, simple scrapers, denticulated, notched tools and unifaces. However, the percentage of these categories varies considerably between sites. Atypical end-scrapers and perforators are also present in all collections.

Common to all assemblages are unifaces that occur in the form of small, rounded plano-convex tools. Among these tools, the convex surface completely preserves the pebble cortex, while the flat (or slightly convex) surface is covered with negatives of centripetal flakes of various sizes.

A comparative analysis of the lithic assemblages from PCs 6–4 deposits reveals substantial similarities in both primary flaking strategies and tool kit composition. The principal distinctions between assemblages lie in the relative frequencies of core and tool types. In each instance, the lithic industry incorporates distinct formal tools, which are typically absent in other localities. These include scrapers with intensive retouch from PC 6 at Karatau and Obi-Mazar-VI sites, "Tayacian" retouched points from PC 6 at Lakhuti-IV and PC 5 at Lakhuti-I, and scrapers displaying perimeter retouch from PC 5 at Lakhuti-I.

The distribution of archaeological materials exhibits certain regularities, both in terms of time and space. The least amount of archaeological material has been recorded in PC 6. The concentration of artefacts per 1 m^2 is 1–2 items in these deposits, with a total excavated area of about 650 m^2 (Obi-Mazar, Karatau, Lakhuti-IV). The assemblages from PC 6 contain considerably more specific formal tools than in later industries. The maximum concentration of artifacts is recorded in assemblages from PC 5, where it is 70–100 items per m^2 (Lakhuti-I and -IV). In PC 4 the concentration of archaeological materials drops again, and specific formal tools practically disappear from the tool kits. In addition, some significant differences between the assemblages of the sites are recorded in this period.

The Khonako-III assemblage differs significantly from other PC 4 complexes at the Obi-Mazar-IV and Lakhuti-IV sites[28,30]. A small percentage of cores, a significant predominance of small flakes and a significant share of tools characterize this assemblage.

The differences in the composition of the lithic assemblages among sites most likely reflect differences in the function of the sites. The low percentage of cores, predominance of small flakes, and high proportion of tools indicate that Khonako served a different purpose than Obi-Mazar and Lakhuti. This makes sense in light of the site's location, as Khonako is located relatively far from the pebble raw material exposed on the riverbed of the Obi-Mazar River, implying that lithic production at Khonako was mainly related to tool manufacture and maintenance. The strategic position of Khonako, located at a relatively high elevation overlooking a large valley, meant that the site likely served as a seasonal hunting ground (based on faunal assemblages from Lower Paleolithic sites across Central Asia, we infer that this would have been primarily wild boar [*Sus scrofa*], bear [*Ursus* sp.], and deer [*Cervus* sp.]) and butchering site. In contrast, the lithic assemblages at Obi-Mazar and Lakhuti bear the hallmarks of workshops characterized by a relative high percentage of cores and many large flakes. These sites are located on the banks of the Obi-Mazar River with easy access to raw material.

It is hypothesized that PC 6 marks the onset of the colonization of what is now Tajikistan by the bearers of the Karatau archaeological culture. During this period, a significant accumulation of archaeological material and a notable diversification of the Karatau culture were documented. Evidence indicates an absence of earlier settlement in the area (MIS 17–19), and the industry's stable composition and ephemeral site occurrences suggests the presence of an external impetus for its emergence in the region, which may be attributed to migration.

The sites of this archaeological culture were most intensively occupied in MIS 13 (PC 5), with a subsequent decrease in the density of artifacts at the sites and a simplification of the typological composition in MIS 11 (PC 4). The absence of any archaeological material in PC

3 (MIS 9) and the presence of a significantly different assemblage in PC 2 (MIS 7)[56] suggest a depopulation of the region during MIS 10.

Supplementary Note 5.4: Possible cultural affinities of the Karatau culture with neighboring territories

Assemblages in the north and west of Central Asia, as well as in the southwest (Iranian plateau), attributed by specialists to the Early Paleolithic, are practically all surface scatters located in open air contexts. Their cultural and chronological position is mainly determined by the presence of bifacial artefacts of archaic appearance in the assemblages (Kazakhstan, Turkmenistan, Iran)[57–59].

In Afghanistan, the only complexes that may be regarded as final Early Pleistocene are the exposed artefacts in the Hazarsum Valley, which also include bifacial tools[51]. The Early Paleolithic zone characterized by biface technologies encompasses nearly the entire Indian Peninsula with the exception of the Soan industries, which are confined to its northwestern periphery (e.g. the upper reaches of the Indus and Soan valleys). In these assemblages, as well as in those of the Karatau culture, the citron, radial, and flat-faced parallel flaking techniques are documented. The associated toolkits predominantly feature choppers and unifacial tools, with a notable absence of clearly defined bifacial tools. Additionally, tools on flakes constitute a significant proportion of the assemblages, primarily comprising of single side-scrapers and notched tools[60,61]. As is the case with sites from Tajikistan, these complexes are concentrated in the foothill and low-mountain zones. They are associated with high terrace deposits and have a raw material base of river alluvium[62,63]. However, the Soan sites are devoid of precise chronologies, and they are dated principally on the basis of geologic and geomorphologic inference. The emergence of Soan industries seems to have occurred later than the Karatau culture, which would make them a continuation of or convergent technology to Karatau sites.

Industries utilizing analogous tools and pebble-based technologies have been documented at numerous Lower Paleolithic sites in central and western China[64–66]. It is noteworthy that all these sites are of a comparable or more pertinently older age than the Karatau sites. Despite the absence of direct analogies between these assemblages and the Karatau culture sites, it is noteworthy that assemblages from China employed all the primary flaking techniques characteristic of the Lower Paleolithic from Tajikistan. The toolkit includes choppers, simple side-scrapers on large flakes, and a variety of notched tools. A significant element in this context is the existence of a particular class of tools, namely, unifaces. Moreover, at certain locations, the assemblage comprises short Tayacian-type points and rounded tools with intensive retouch (tablet-shaped objects), almost similar to the scraper from Lakhuti-I. The presence of bifacial tools at certain sites in China represents the most significant difference in comparison to the Karatau complexes. The question of the chronological appearance of bifaces in China remains a subject of debate. The emergence of bifaces in the southeastern regions of Asia has been dated to the end of the Early Pleistocene to the onset of the Middle Pleistocene. In contrast, the occurrence of bifacial tools in the central part of the region is significantly later, with some researchers suggesting a minimum age of 300,000 years. Even in those industries where there are certainly bifacial reduction techniques, its use was irregular, and unifacial processing was predominantly used[67,68]. The

currently available data allow us to argue for a possible connection of the Early Paleolithic industries of Tajikistan, with the eastern part of the continent. These parallels were also noted by V.A. Ranov, when he wrote that "Undoubtedly, there is a huge, still insufficiently analyzed similarity between the Lower Paleolithic monuments of China and the Karatau culture..."[52]. Consequently, the investigation of potential cultural affinities between the Karatau culture and the Early Paleolithic complexes within China, as well as with the Soan industries, appears to be a promising avenue of research in future projects. It is evident that there are parallels between these technocomplexes, both in terms of the characteristics of lithic industries and the adaptation strategies employed. However, the establishment of potential connections in the Middle Pleistocene between these regions necessitates further study and substantiation.

References

1. Buylaert, J.-P. *et al.* A luminescence dating study of the upper part of the loess-palaeosol sequence at Kuldara, Khovaling Loess Plateau, Tajikistan. *Quaternary Geochronology* **82**, 101545 (2024).
2. Challier, A. *et al.* A detailed quartz and feldspar luminescence chronology for the Khonako II loess section (Southern Tajikistan). *Quaternary Geochronology* **83**, 101571 (2024).
3. Kulakova, E. & Kurbanov, R. First Paleomagnetic Record of the Matuyama-Brunhes Transition in the Loess-Paleosol Series of Tajikistan. in *Problems of Geocosmos—2022* (eds Kosterov, A., Lyskova, E., Mironova, I., Apatenkov, S. & Baranov, S.) 67–84 (Springer International Publishing, Cham, 2023). doi:10.1007/978-3-031-40728-4_6.
4. Kulakova, E. P., Kurbanov, R. N. & Sosin, P. M. New high-resolution magnetostratigraphic record of the last 1.2 Myr from loess-paleosol section Khonako-II in Southern Tajikistan, Central Asia. *Quaternary Science Reviews* **371**, 109689 (2026).
5. Tokareva, O. A. *et al.* Structure and properties of paleosols of the last two interglacial cycles of the Khovaling Loess Plateau, Tajikistan (Obi–Mazar section). *Quat. res.* **124**, 47–63 (2025).
6. Kulakova, E. P. *et al.* Stratigraphy and geochronology of the Kuldara Early Paleolithic Site (Tajikistan). *Geomorfologiâ i paleogeografiâ* **55**, (2024).
7. Autzen, M., Andersen, C. E., Bailey, M. & Murray, A. S. Calibration quartz: An update on dose calculations for luminescence dating. *Radiation Measurements* **157**, 106828 (2022).

8. Bøtter-Jensen, L., Andersen, C. E., Duller, G. A. T. & Murray, A. S. Developments in radiation, stimulation and observation facilities in luminescence measurements. *Radiation Measurements* **37**, 535–541 (2003).
9. Stevens, T. *et al.* Ice-volume-forced erosion of the Chinese Loess Plateau global Quaternary stratotype site. *Nat Commun* **9**, 983 (2018).
10. Volvakh, N. E. *et al.* First high-resolution luminescence dating of loess in Western Siberia. *Quaternary Geochronology* **73**, 101377 (2022).
11. Challier, A. Detailed quartz and feldspar luminescence chronologies of loessic Palaeolithic sites in Tajikistan. PhD thesis. Department of Physics, Technical University of Denmark. https://backend.orbit.dtu.dk/ws/portalfiles/portal/390510697/PhD_thesis_-_Am_lie_Juliette_Marie_Challier.pdf. (2024).
12. Li, B. & Li, S. A reply to the comments by Thomsen et al. on" Luminescence dating of K-feldspar from sediments: A protocol without anomalous fading correction". (2012).
13. Murray, A. S., Helsted, L. M., Autzen, M., Jain, M. & Buylaert, J. P. Measurement of natural radioactivity: Calibration and performance of a high-resolution gamma spectrometry facility. *Radiation Measurements* **120**, 215–220 (2018).
14. Murray, A. S., Marten, R., Johnston, A. & Martin, P. Analysis for naturally occuring radionuclides at environmental concentrations by gamma spectrometry. *Journal of Radioanalytical and Nuclear Chemistry, Articles* **115**, 263–288 (1987).
15. Cresswell, A. J., Carter, J. & Sanderson, D. C. W. Dose rate conversion parameters: Assessment of nuclear data. *Radiation Measurements* **120**, 195–201 (2018).
16. Martin, L., Mercier, N. & Incerti, S. Geant4 simulations for sedimentary grains in infinite matrix conditions: The case of alpha dosimetry. *Radiation Measurements* **70**, 39–47 (2014).
17. Mejdahl, V. Thermoluminescence dating: beta-dose attenuation in quartz grains. *Archaeometry* **21**, 61–72 (1979).

18. Schmidt, C., Bösken, J. & Kolb, T. Is there a common alpha-efficiency in polymineral samples measured by various infrared stimulated luminescence protocols? *Geochronometria* **45**, 160–172 (2018).

19. Aitken, M. J. Thermoluminescence Dating Academic Press New York. (1985).

20. Prescott, J. R. & Hutton, J. T. Cosmic ray contributions to dose rates for luminescence and ESR dating: Large depths and long-term time variations. *Radiation Measurements* **23**, 497–500 (1994).

21. Dodonov, A. E. *Četvertičnyj Period Srednej Azii: Stratigrafija, Korreljacija, Paleografija*. (GEOS, 2002).

22. Penkov, A. V. & Gamov, L. N. Paleomagnetic benchmarks in the Pliocene-Quaternary strata of Southern Tajikistan. *Granitsa neogena i chetvertichnoi sistemy. M.: Nauka (Publ.)* 184–189 (1980).

23. Ranov, V. A. & Zhukov, V. A. Works of the Stone Age study group in 1976. in *Archaeological works in Tajikistan* vol. XVI 9–30 (1982).

24. Ranov, V. A. Works of the Stone Age study group in 1979. in *Archaeological works in Tajikistan* vol. XIX 11–36 (1986).

25. Ranov, V. A., Dodonov, A. E., Lomov, S. P., Pakhomov, M. M. & Penkov, A. V. Kuldara—novyi nizhnepaleoliticheskij pamyatnik Yuzhnogo Tadjikistana (Kuldara—a new Lower Paleolithic site in Southern Tadjikistan). *Bulletin Komissii po izucheniyu chetvertichnogo perioda* **56**, 65–74 (1987).

26. Ranov, V. A. & Amosova, A. G. Works of the Stone Age study group in 1982. in *Archaeological works in Tajikistan* vol. XXII 161–194 (1990).

27. Ranov, V. A. & Amosova, A. G. Completion of excavations of the Karatau-I site in 1986. in *Archaeological works in Tajikistan* vol. XXVI 271–310 (2005).

28. Ranov, V. A. Excavations in 4-6 paleosols of the Obi-Mazar loess-paleosol section in 1995 and 1997. in *Archaeological works in Tajikistan* vol. XXX 14–32 (2005).

29. Ranov, V. A. & Schäfer, J. Excavations of the fourth paleosol of the Khonako-III section in 2000. in *Archaeological works in Tajikistan* vol. XXIX 71–120 (2004).

30. Anoikin, A. *et al.* Lakhuti-IV – A new site of the early Palaeolithic in Central Asia (Tajikistan). *Archaeological Research in Asia* **35**, 100466 (2023).

31. Heinemeier, J., Olsen, J., Klein, M. & Mous, D. The new extended HVE 1 MV multi-element AMS system for low background installed at the Aarhus AMS Dating Centre. *Nuclear Instruments and Methods in Physics Research Section B: Beam Interactions with Materials and Atoms* **361**, 143–148 (2015).

32. Nishiizumi, K. *et al.* Absolute calibration of 10Be AMS standards. *Nuclear Instruments and Methods in Physics Research Section B: Beam Interactions with Materials and Atoms* **258**, 403–413 (2007).

33. Nishiizumi, K. Preparation of 26Al AMS standards. *Nuclear Instruments and Methods in Physics Research Section B: Beam Interactions with Materials and Atoms* **223–224**, 388–392 (2004).

34. Sørensen, A. L. *et al.* CosmoChron: A versatile age-depth modeling approach using cosmogenic nuclides and direct age constraints. *Quaternary Geochronology* **85**, 101618 (2024).

35. Sørensen, A. L., Hansen, T. M., Falk, F. A., Olsen, J. & Knudsen, M. F. Quantifying uncertainty in stratigraphic alignment of geological signals using probabilistic dynamic time warping. *Quaternary Science Reviews* **369**, 109632 (2025).

36. Tarantola, A. *Inverse Problem Theory and Methods for Model Parameter Estimation*. (Society for Industrial and Applied Mathematics, 2005). doi:10.1137/1.9780898717921.

37. Mosegaard, K. & Tarantola, A. Monte Carlo sampling of solutions to inverse problems. *J. Geophys. Res.* **100**, 12431–12447 (1995).

38. Hansen, T. M., Cordua, K. S., Looms, M. C. & Mosegaard, K. SIPPI: A Matlab toolbox for sampling the solution to inverse problems with complex prior information. *Computers & Geosciences* **52**, 470–480 (2013).

39. Cohen, K. M. & Gibbard, P. L. Global chronostratigraphical correlation table for the last 2.7 million years, version 2019 QI-500. *Quaternary International* **500**, 20–31 (2019).

40. Knudsen, M. F. *et al.* New cosmogenic nuclide burial-dating model indicates onset of major glaciations in the Alps during Middle Pleistocene Transition. *Earth and Planetary Science Letters* **549**, 116491 (2020).

41. Nørgaard, J., Jansen, J. D., Neuhuber, S., Ruszkiczay-Rüdiger, Z. & Knudsen, M. F. P–PINI: A cosmogenic nuclide burial dating method for landscapes undergoing non-steady erosion. *Quaternary Geochronology* **74**, 101420 (2023).

42. Pan, Q. *et al.* The Jaramillo subchron in Chinese loess-paleosol sequences. *Palaeogeography, Palaeoclimatology, Palaeoecology* **572**, 110423 (2021).

43. Mahgoub, A. N., Korte, M. & Panovska, S. Characteristics of the Matuyama-Brunhes Magnetic Field Reversal Based on a Global Data Compilation. *JGR Solid Earth* **128**, e2022JB025286 (2023).

44. Lisiecki, L. E. & Raymo, M. E. A Pliocene-Pleistocene stack of 57 globally distributed benthic δ^{18} O records. *Paleoceanography* **20**, 2004PA001071 (2005).

45. Goovaerts, P. *Geostatistics for Natural Resources Evaluation*. (Oxford University Press, New York, 1997).

46. Lazarenko, A. A. & Ranov, V. A. Karatau 1—drevneishij paleoliticheskij pamyatnik v lesseh Srednej Azii (Karatau 1—the oldest Paleolithic site in the loess of Central Asia). *Bulleten Komissii po izucheniyu chetvertichnogo perioda* **47**, 45–57 (1977).

47. Lazarenko, A. A. & Ranov, V. A. New Paleolithic site of Karatau I in Southern Tajikistan. in *Achievements of Central Asian Archaeology* (ed. Abetekov, A. A.) vol. 3 69–73 (Nauka, Leningrad, 1975).

48. Ranov, V. A. *et al.* Toward a New Outline of the Soviet Central Asian Paleolithic [and Comments and Reply]. *Current Anthropology* **20**, 249–270 (1979).

49. Ding, Z. L. *et al.* The loess record in southern Tajikistan and correlation with Chinese loess. *Earth and Planetary Science Letters* **200**, 387–400 (2002).

50. Ranov, V. A. The 'loessic palaeolithic' in South Tadjikistan, central Asia: Its industries, chronology and correlation. *Quaternary Science Reviews* **14**, 731–745 (1995).

51. Ranov, V. A. & Karimova, G. R. *Kamennyi Vek Afgano-Tadzhikskoi Depressii (Stone Age of the Afghan-Tajik Depression)*. (Devashtish Press: Dushanbe (in Russian), 2005).

52. Ranov, V. A. & Schäfer, J. Loess paleolithic. *Archeology, Ethnography and Anthropology of Eurasia* **2**, 20–32 (2000).

53. Schäfer, J., Laurat, T., Ranov, V. A. & Sosin, P. M. Das Altpaläolithikum des 4. Paläobodenkomplexes von Obi-Mazar (Tadschikistan). in *Erkenntnisjäger. Kultur und Umwelt des frühen Menschen. Festschrift für Dietrich Mania* (ed. Meller, H.) 509–535 (Landesmuseum für Vorgeschichte, Halle, 2003).

54. Hiscock, P., Turq, A., Faivre, J. & Bourguignon, L. Quina Procurement and Tool Production. in *Lithic Materials and Paleolithic Societies* (eds Adams, B. & Blades, B. S.) 232–246 (Wiley, 2009). doi:10.1002/9781444311976.ch17.

55. Sharon, G. Acheulian Giant-Core Technology: A Worldwide Perspective. *Current Anthropology* **50**, 335–367 (2009).

56. Schäfer, J., Ranov, V. A. & Sosin, P. M. THE 'CULTURAL EVOLUTION' OF MAN AND THE CHRONOSTRATIGRAPHICAL BACKGROUND OF CHANGING ENVIRONMENTS IN THE LOESS PALAEOSOIL SEQUENCES OF OBI-MAZAR AND KHONAKO (TADJIKISTAN). *Anthropologie (1962-)* **36**, 121–135 (1998).

57. Derevyanko, A. P. Three global human migrations in Eurasia. in *Initial human peopling of North, Central, and Middle Asia* (ed. Shunkov, M. V.) vol. 2 (Institute of Archaeology and Ethnography, Siberian Branch of the Russian Academy of Sciences, Novosibirsk, 2017).

58. Shoaee, M. J., Vahdati Nasab, H. & Petraglia, M. D. The Paleolithic of the Iranian Plateau: Hominin occupation history and implications for human dispersals across southern Asia. *Journal of Anthropological Archaeology* **62**, 101292 (2021).
59. Vishnyatsky, L. B. *The Paleolithic of Central Asia and Kazakhstan*. (European House, St. Petersburg, 1996).
60. Chauhan, P. R. Soanian cores and core-tools from Toka, northern India: Towards a new typo-technological organization. *Journal of Anthropological Archaeology* **26**, 412–441 (2007).
61. Chauhan, P. R. The technological organization of the Soanian Palaeolithic industry: A general "typo-qualitative" description of a large core-and-flake assemblage in surface context from the Siwalik hills of northern India. in *Issues and Themes in Anthropology: A Festschrift in Honour of Professor D.K. Bhattacharya* 287–336 (Palaka Prakashan, Delhi, 2005).
62. Petraglia, M. D. The Early Paleolithic of the Indian Subcontinent: Hominin Colonization, Dispersals and Occupation History. in *Out of Africa I* (eds Fleagle, J. G., Shea, J. J., Grine, F. E., Baden, A. L. & Leakey, R. E.) 165–179 (Springer Netherlands, Dordrecht, 2010). doi:10.1007/978-90-481-9036-2_11.
63. Sali, S. A. *Stone Age India*. (Shankar Publishers, Aurangabad, 1990).
64. Bar-Yosef, O. & Wang, Y. Paleolithic Archaeology in China. *Annu. Rev. Anthropol.* **41**, 319–335 (2012).
65. Gao, X. Paleolithic Cultures in China: Uniqueness and Divergence. *Current Anthropology* **54**, S358–S370 (2013).
66. Zhang, S. The Early Palaeolithic of China. in *Paleoanthropology and Paleolithic Archaeology in the People's Republic of China* (eds Wu, X. J. & Olsen, J. W.) 40 (Routledge, London, 2009).
67. Lei, L. *et al.* Production patterns and strategies of the Acheulean large flakes. *Acta Anthropologica Sinica* vol. 39 183–192 (2020).

68. Shen, C. & Qi, W. Lithic technological variability of the Middle Pleistocene in the eastern Nihewan Basin, northern China. *Asian Perspectives* 281–301 (2004).
