# Supplementary material for: Absolute chronology of the Early Palaeolithic Karatau Culture in Central Asia
Source: Nat Commun. 2026 Jul 1;17:8217. doi: 10.1038/s41467-026-74938-5 (PMC13462899; doi:10.1038/s41467-026-74938-5)
Supplement: Supplementary file 3 [file 41467_2026_74938_MOESM2_ESM.pdf]

Description of Additional Supplementary files

This document provides an overview and brief description of all supplementary files associated with the paper.

Supplementary Data 1, 3, 4, and 9–11 were used to construct the age–depth models presented in Figure 4 and Figure S3. Supplementary Data 9–11 were used to develop the alignment models shown in Figure S4. Supplementary Data 12–14 contain the age-depth model results presented in Figure 4.

Supplementary Data 1–3: Luminescence Data

These datasets contain the luminescence dating results used in this study, including both data presented in this study and previously published data.

Supplementary Data 1;

Luminescence dating results for the Khonako-II and Obi-Mazar sections presented in this study.

- Obi-Mazar (n = 40)
- Khonako-II (n = 145)

Supplementary Data 2;

Radionuclide activity concentrations obtained by high-resolution gamma spectrometry, together with dry infinite matrix beta, gamma, and alpha dose rates for the Khonako-II and Obi-Mazar sections.

Supplementary Data 3

Previously published luminescence ages used in this study:

- Khonako-II: Challier, A. et al., *Quaternary Geochronology* 83, 101571 (2024); n = 23
- Kuldara: Buylaert, J.-P. et al., *Quaternary Geochronology* 82, 101545 (2024); n = 78

Supplementary Data 4–8: Cosmogenic Nuclide Data

These datasets contain measured cosmogenic ^{26}Al and ^{10}Be concentrations from the studied samples (n = 28).

Supplementary Data 4

Overview of the cosmogenic nuclide dataset.

Supplementary Data 5

Blank information for each batch.

Supplementary Data 6

Detailed analytical information for the cosmogenic nuclide measurements.

Supplementary Data 7

Beryllium-10 (^{10}Be) results from the Aarhus Accelerator Mass Spectrometry Center.

Supplementary Data 8

Aluminium-26 (^{26}Al) results from the Aarhus Accelerator Mass Spectrometry Center.

Supplementary Data 9–11: Magnetic Susceptibility Data

These datasets contain the magnetic susceptibility data used in this study.

Supplementary Data 9

Magnetic susceptibility data for the Obi-Mazar section, including both data presented in this study and previously published datasets.

Data presented in this study:

- Lower half of the Obi-Mazar section; n = 1223

Previously published data:

- Upper part of Obi-Mazar: Tokareva, O. A. et al., *Quaternary Research* 124, 47–63 (2025); n = 1094

Supplementary Data 10

Previously published magnetic susceptibility data for the Kuldara section:

- Kulakova, E. P. et al., *Geomorfologiya i Paleogeografiya* 55 (2024); n = 2861

Supplementary Data 11

Previously published magnetic susceptibility data for the Khonako-II section:

- Kulakova, E., Kurbanov, R. & Sosin, P., *Quaternary Science Reviews* 371, 109689 (2026); n = 1038

Supplementary Data 12–14: Age-Depth Models

These datasets contain the age-depth modeling outputs presented in Figure 4 of the main text.

Supplementary Data 12**:** Obi-Mazar

Supplementary Data 13**:** Kuldara

Supplementary Data 14**:** Khonako-II

Supplementary Data 15–17: Biomarker Data

These datasets contain previously published biomarker data, including:

Supplementary Data 15**:** Stable carbon isotope values ($\delta^{13}C_{bulk}$; Fig. 5D; n = 54)

Supplementary Data 16**:** n-alkane ratios ($(C_{27}+C_{29})/(C_{31}+C_{33})$; Fig. 5E; n = 32)

Supplementary Data 17**:** Polycyclic aromatic hydrocarbons (PAHs; Fig. 5F; n = 32) Data

are from:

- Chirakkal, A. et al., *Palaeogeography, Palaeoclimatology, Palaeoecology* 663, 112786 (2025)
- Chirakkal, A. et al., *Organic Geochemistry* 212, 105088 (2026)

Supplementary Data 18: Museum Accession Numbers

This dataset provides a list of museum accession numbers for the lithic artefacts shown in Figures 3 and 5. The artefacts are located at the National Academy of Science of Tajikistan in Dushanbe.
