# Supplementary material for: Absolute chronology of the Early Palaeolithic Karatau Culture in Central Asia
Source: Nat Commun. 2026 Jul 1;17:8217. doi: 10.1038/s41467-026-74938-5 (PMC13462899; doi:10.1038/s41467-026-74938-5)
Supplement: Supplementary file 4 [file 41467_2026_74938_MOESM9_ESM.pdf]

Corresponding author(s): Aske Lohse Sørensen and Mads Faurschou Knudsen

Last updated by author(s): Nov 11, 2025

Reporting Summary

Nature Portfolio wishes to improve the reproducibility of the work that we publish. This form provides structure for consistency and transparency in reporting. For further information on Nature Portfolio policies, see our Editorial Policies and the Editorial Policy Checklist.

Statistics

For all statistical analyses, confirm that the following items are present in the figure legend, table legend, main text, or Methods section.

n/a	Confirmed	
☐	☒	The exact sample size (*n*) for each experimental group/condition, given as a discrete number and unit of measurement
☒	☐	A statement on whether measurements were taken from distinct samples or whether the same sample was measured repeatedly
☒	☐	The statistical test(s) used AND whether they are one- or two-sided<br>*Only common tests should be described solely by name; describe more complex techniques in the Methods section.*
☐	☒	A description of all covariates tested
☐	☒	A description of any assumptions or corrections, such as tests of normality and adjustment for multiple comparisons
☐	☒	A full description of the statistical parameters including central tendency (e.g. means) or other basic estimates (e.g. regression coefficient) AND variation (e.g. standard deviation) or associated estimates of uncertainty (e.g. confidence intervals)
☒	☐	For null hypothesis testing, the test statistic (e.g. *F*, *t*, *r*) with confidence intervals, effect sizes, degrees of freedom and *P* value noted<br>*Give P values as exact values whenever suitable.*
☐	☒	For Bayesian analysis, information on the choice of priors and Markov chain Monte Carlo settings
☒	☐	For hierarchical and complex designs, identification of the appropriate level for tests and full reporting of outcomes
☒	☐	Estimates of effect sizes (e.g. Cohen's *d*, Pearson's *r*), indicating how they were calculated

Our web collection on statistics for biologists contains articles on many of the points above.

Software and code

Policy information about availability of computer code

Data collection	Not relevant to this study.

Data analysis

Age-depth models were constructed using the CosmoChron method, a probabilistic inverse modeling approach that integrates cosmogenic 26Al-10Be concentrations and/or direct age constraints. The parameters in the CosmoChron model include the i) mean, variance, and correlation range of the accumulation rates, ii) hiatus durations associated with observed unconformities, iii) pre-burial exposure histories associated with the 26Al-10Be samples, and iv) paleomagnetic lock-in depth range. Prior knowledge is used to constrain each of these parameters, including the constraint that the accumulation rate must be positive to ensure that the age increases with depth. The CosmoChron forward model generates age-depth relationships and 26Al-10Be concentrations at specific depths, which are subsequently compared against empirical observations. The inverse problem is solved using the extended Metropolis algorithm, a Markov Chain Monte Carlo method, which is implemented using the SIPPI toolbox for MATLAB.

Magnetic susceptibility records were probabilistically aligned using the P-DTW (Probabilistic Dynamic Time Warping) algorithm. The P-DTW algorithm is a method that quantifies the uncertainty associated with multiple stratigraphic alignments. This allow to include transposed ages across the sites into the CosmoChron age-depth.

The softeware used are available on GitHub.com: (CosmoChron: https://github.com/aaaassssskkkkeeee/CosmoChron), (P-DTW: https://github.com/aaaassssskkkkeeee/P-DTW), (P-PINI: https://github.com/cosmojesper/ppini) and (SIPPI: https://github.com/cultpenguin/sippi). All versions correspond to those available as of 8 April 2026

MATLAB R2025a were used.

For manuscripts utilizing custom algorithms or software that are central to the research but not yet described in published literature, software must be made available to editors and reviewers. We strongly encourage code deposition in a community repository (e.g. GitHub). See the Nature Portfolio guidelines for submitting code & software for further information.

Data

Policy information about availability of data

All manuscripts must include a data availability statement. This statement should provide the following information, where applicable:

- Accession codes, unique identifiers, or web links for publicly available datasets
- A description of any restrictions on data availability
- For clinical datasets or third party data, please ensure that the statement adheres to our policy

New luminescence and cosmogenic nuclide data used in this study are provided in the Supplementary Information. The archaeological artifacts analyzed in this work are located at the National Academy of Science of Tajikistan in Dushanbe and are available upon request.

Research involving human participants, their data, or biological material

Policy information about studies with human participants or human data. See also policy information about sex, gender (identity/presentation), and sexual orientation and race, ethnicity and racism.

Reporting on sex and gender	Not relevant for our study.
Reporting on race, ethnicity, or other socially relevant groupings	Not relevant for our study.
Population characteristics	Not relevant for our study.
Recruitment	Not relevant for our study.
Ethics oversight	Not relevant for our study.

Note that full information on the approval of the study protocol must also be provided in the manuscript.

Field-specific reporting

Please select the one below that is the best fit for your research. If you are not sure, read the appropriate sections before making your selection.

☐ Life sciences ☐ Behavioural & social sciences ☒ Ecological, evolutionary & environmental sciences

For a reference copy of the document with all sections, see nature.com/documents/nr-reporting-summary-flat.pdf

Ecological, evolutionary & environmental sciences study design

All studies must disclose on these points even when the disclosure is negative.

Study description

This study provides the first absolute age framework for the Early Paleolithic Karatau Culture in Central Asia, which is defined by the rich lithic assemblages found in paleosols in the Khovaling Loess Plateau, Tajikistan. The age constraints are provided through a new multi-method approach that combines luminescence ages, cosmogenic 26Al-10Be concentrations, and magnetostratigraphic

boundaries into a probabilistic inverse age-depth model.

Research sample

The study analyzed stratified loess–paleosol sequences and associated archaeological artifacts from three sections from the Khovaling Loess Plateau in southern Tajikistan: Obi-Mazar (latitude: 38.302472°, longitude: 69.891592°, Elevation of the surface: 1300 m), Kuldara (latitude: 38.282769°, longitude: 69.885639°, Elevation of the surface: 1300 m), and Khonako-II (latitude: 38.359272°, longitude: 70.046386°, Elevation of the surface: m). These sites were chosen for their rich ensemble of litichs.

We use 286 luminescence ages, 16 pairs of cosmogenic 26Al and 10Be concentrations, six paleomagnetic boundaries along with 6216 magnetic susceptibility datapoints from three loess-paleosol sequences. 101 luminescence ages are puplished (1,2) along the 4993 magnetic susceptibility datapoints (4,5,6) and the six paleomagnetic boundaries (3,4), as summaries in the supplementary material table S1.

1. Buylaert, J.-P. et al. A luminescence dating study of the upper part of the loess-palaeosol sequence at Kuldara, Khovaling Loess Plateau, Tajikistan. Quaternary Geochronology 82, 101545 (2024).
2. Challier, A. et al. A detailed quartz and feldspar luminescence chronology for the Khonako II loess section (Southern Tajikistan). Quaternary Geochronology 83, 101571 (2024).
3. Kulakova, E. & Kurbanov, R. First Paleomagnetic Record of the Matuyama-Brunhes Transition in the Loess-Paleosol Series of Tajikistan. in Problems of Geocosmos—2022 (eds. Kosterov, A., Lyskova, E., Mironova, I., Apatenkov, S. & Baranov, S.) 67–84 (Springer International Publishing, Cham, 2023). doi:10.1007/978-3-031-40728-4_6.
4. Kulakova, E., Kurbanov, R. & Sosin, P. New high-resolution magnetostratigraphic record of the last 1.2 Myr from loess-paleosol section Khonako-II in Southern Tajikistan, Central Asia. Quaternary Science Reviews (in review).
5. Tokareva, O. A. et al. Structure and properties of paleosols of the last two interglacial cycles of the Khovaling Loess Plateau, Tajikistan (Obi–Mazar section). Quat. res. 124, 47–63 (2025).
6. Kulakova, E. P. et al. Stratigraphy and geochronology of the Kuldara Early Paleolithic Site (Tajikistan). Geomorfologiâ i paleogeografiâ 55, (2024).

Sampling strategy

The dating methods and stratigrapich spacing of the samples were chosen to obtain a comprehensive chronology of the sites. The luminescence ages are used to constrain the age-depth models in the upper ~250 ka; the cosmogenic nuclides samples are used to constrain the age-depth models in the 300 to 600 ka age range because the majority of stone tools derive from PC4, PC5, and PC6. The older parts (>700 ka) of the age models are constrained by paleomagnetic boundaries. The magnetic susceptibility records are used to refine and augment the age-depth models in the ~300-700 ka range.
No initial sample size calculations were made.

Data collection

Standard sample preparation techniques for luminescence dating including HCl (10%) and H2O2 (10%) treatments and wet-sieving were used to extract the polymineral 40-63µm fraction from each sample (3). These grains were mounted as large (~8 mm diameter) aliquots on stainless steel cups and measured in a standard Risø TL/OSL reader equipped with infra-red (IR, 870 nm) stimulation and a calibrated 90Sr/90Y beta source (4,5). The chosen SAR post-IR IRSL protocol involved two 200 s IR stimulations, one at 200 degree Celsius to remove unstable charge and a subsequent stimulation at 290 degree Celsius to access that signal of interest (pIRIR200,290). At end of each SAR cycle there was an elevated temperature IR (325 degree Celsius, 200 s) cleanout step to reduce recuperation. This pIRIR200,290 protocol has been applied successfully in several luminescence dating studies on loess (e.g. 6,7). Following Challier et al. (2), the test dose size was kept at ~100% of the estimated dose for the Khonako II and Obi Mazar samples, whereas for the Kuldara data a test ranging between 30-50% was used (1). The size of these test doses was chosen based on successful dose recovery tests.
A typical dose response curve is shown in Fig. S1a for sample 249038 collected below PC2 at Obi Mazar. It can be seen that the natural sensitivity corrected light level lies well below laboratory saturation and interpolation can be easily achieved. Fig. S1b shows the dose recovery data for the top sample from Obi Mazar (249001). In this experiment a range of laboratory doses was added on top of the natural dose and measured with SAR (using test doses approximately equal to the measured doses). The slope of the linear fit is 1.09 suggesting that our SAR pIRIR200,290 protocol can accurately measure a dose given prior to a heat treatment.
It has been shown by Challier (8) and others (e.g. 6,9) that the pIRIR200,290 signal does not seem to fade in the laboratory and does not require correction for instability. However, the pIRIR200,290 signal does contain a very-hard-to-bleach component which needs to be subtracted prior to age calculation. This presumed residual dose at deposition was estimated by Challier et al. (2) to be 9.2±0.4 Gy based on very young loess and modern dust samples. The measured pIRIR200,290 De values with this tresidual subtracted are given in Table S2.
For each sample, the radionuclide concentrations (238U, 226Ra, (210Pb), 232Th and 40K) were measured on dried and powdered subsamples using high-resolution gamma spectrometry following Murrey (10,11) (Table S3). Radionuclide concentrations were converted to dry, infinite matrix, alpha, beta and gamma dose rates using the conversion factors of Cresswell et al. (12). Alpha and beta attenuation factors follow Martin et al. (13) and Mejdahl (14), respectively. To allow for the different luminescence efficiency of alpha compared to beta/gamma radiation an a-value of 0.09±0.02 was adopted (14). The individual dry dose rate components were corrected for moisture content using the correction factors proposed by Aitken (16). For Khonako II and Obi Mazar the water content values are based on the depth-dependent water content model developed by Challier (8) for the Khonako III site. For Kuldara the water content values presented in Buylaert et al.1 were used. A small internal beta dose rate from 40K was incorporated (0.24±0.01 Gy/ka, based on 12.5% K in K-rich feldspar grains). The internal U, Th alpha dose rate contribution was assumed to be 0.10±0.05 Gy/ka. Cosmic ray dose rate contribution was calculated following Prescott and Hutten (17). The total dose rates, water content values and final pIRIR200,290 ages are also presented in Table S2.

Samples for measuring the cosmogenic 26Al and 10Be concentrations were initially crushed and sieved to a 250–500µm grain size fraction, followed by magnetic separation. The grains were then treated with hydrochloric acid (HCl) to dissolve calcium carbonates, then cleaned in heated aqua regia to remove remaining impurities. Quartz and feldspar were separated by flotation and heavy liquate separation. The quartz fraction was then etched with hydrofluoric acid (HF) to remove meteoric10Be from the grain surfaces. Finally, ~20g of quartz from each sample was fully digested in HF along with an in-house 9Be and 27Al carrier. Chloride conversion was achieved through repeated addition and evaporation of 6M HCl. Beryllium and aluminum were then isolated using ion exchange chromatography. The oxidized samples were mixed ~1:4 with Nb powder (BeO) and ~1:1.5 Ag power (Al2O3) and pressed into cathodes for AMS analysis.
The 26Al/27Al and 10Be/9Be ratios were measured using the multi-element AMS system at the Aarhus AMS Centre (18). Beryllium results were normalized to the ICN standard 01-5-4, which has a certified 10Be/9Be ratio of 2.851×10^-12 (19); reported errors

include a 1.1 % uncertainty associated with the standard. Aluminum results were normalized to the ICN standard 01-4-3, with a 26Al/27Al ratio of 10.65×10^-12 (20); uncertainties include a 1.2% contribution from the standard. For each batch, the 10Be/9Be and 26Al/27Al ratios of the corresponding procedural blanks were subtracted from the sample measurements.

Magnetic susceptibility measurements were taken along the section using a portable PIMV kappameter.

Luminescence samples were collected by NT, AC, AK and J-PB and processed by NT, AK and J-PB. Cosmogenic nuclide samples were collected by V.A. Ranov, TUK, AA, EPK, ALS, J-PB and MFK and processed by EPK and ALS with assistance from lab technician Birte Lindahl Eriksen and Rikke Brok Jensen at Aarhus University, while the 26Al and 10Be concentrations were measured by JO at Aarhus AMSC. Lithics were collected and analyzed by AA with assistance from KAK, TUK, PMS, IA, AS, VP, AR and RNK. New magnetic susceptibility data from the lower Obi-Mazar section were collected by OT.

References
1. Buylaert, J.-P. et al. A luminescence dating study of the upper part of the loess-palaeosol sequence at Kuldara, Khovaling Loess Plateau, Tajikistan. Quaternary Geochronology 82, 101545 (2024).
2. Challier, A. et al. A detailed quartz and feldspar luminescence chronology for the Khonako II loess section (Southern Tajikistan). Quaternary Geochronology 83, 101571 (2024).
3. Murray, A. et al. Optically stimulated luminescence dating using quartz. Nat Rev Methods Primers 1, 72 (2021).
4. Autzen, M., Andersen, C. E., Bailey, M. & Murray, A. S. Calibration quartz: An update on dose calculations for luminescence dating. Radiation Measurements 157, 106828 (2022).
5. Bøtter-Jensen, L., Andersen, C. E., Duller, G. A. T. & Murray, A. S. Developments in radiation, stimulation and observation facilities in luminescence measurements. Radiation Measurements 37, 535–541 (2003).
6. Stevens, T. et al. Ice-volume-forced erosion of the Chinese Loess Plateau global Quaternary stratotype site. Nat Commun 9, 983 (2018).
7. Volvakh, N. E. et al. First high-resolution luminescence dating of loess in Western Siberia. Quaternary Geochronology 73, 101377 (2022).
8. Challier, A. Detailed quartz and feldspar luminescence chronologies of loessic Palaeolithic sites in Tajikistan. PhD thesis. Department of Physics, Technical University of Denmark. https://backend.orbit.dtu.dk/ws/portalfiles/portal/390510697/PhD_thesis_-_Am_lie_Juliette_Marie_Challier.pdf. (2024).
9. Li, B. & Li, S. A reply to the comments by Thomsen et al. on" Luminescence dating of K-feldspar from sediments: A protocol without anomalous fading correction". (2012).
10. Murray, A. S., Helsted, L. M., Autzen, M., Jain, M. & Buylaert, J. P. Measurement of natural radioactivity: Calibration and performance of a high-resolution gamma spectrometry facility. Radiation Measurements 120, 215–220 (2018).
11. Murray, A. S., Marten, R., Johnston, A. & Martin, P. Analysis for naturally occuring radionuclides at environmental concentrations by gamma spectrometry. Journal of Radioanalytical and Nuclear Chemistry, Articles 115, 263–288 (1987).
12. Cresswell, A. J., Carter, J. & Sanderson, D. C. W. Dose rate conversion parameters: Assessment of nuclear data. Radiation Measurements 120, 195–201 (2018).
13. Martin, L., Mercier, N. & Incerti, S. Geant4 simulations for sedimentary grains in infinite matrix conditions: The case of alpha dosimetry. Radiation Measurements 70, 39–47 (2014).
14. Mejdahl, V. Thermoluminescence dating: beta-dose attenuation in quartz grains. Archaeometry 21, 61–72 (1979).
15. Schmidt, C., Bösken, J. & Kolb, T. Is there a common alpha-efficiency in polymineral samples measured by various infrared stimulated luminescence protocols? Geochronometria 45, 160–172 (2018).
16. Aitken, M. J. Thermoluminescence Dating Academic Press New York. (1985).
17. Prescott, J. R. & Hutton, J. T. Cosmic ray contributions to dose rates for luminescence and ESR dating: Large depths and long-term time variations. Radiation Measurements 23, 497–500 (1994).
18. Heinemeier, J., Olsen, J., Klein, M. & Mous, D. The new extended HVE 1 MV multi-element AMS system for low background installed at the Aarhus AMS Dating Centre. Nuclear Instruments and Methods in Physics Research Section B: Beam Interactions with Materials and Atoms 361, 143–148 (2015).
19. Nishiizumi, K. et al. Absolute calibration of 10Be AMS standards. Nuclear Instruments and Methods in Physics Research Section B: Beam Interactions with Materials and Atoms 258, 403–413 (2007).
20. Nishiizumi, K. Preparation of 26Al AMS standards. Nuclear Instruments and Methods in Physics Research Section B: Beam Interactions with Materials and Atoms 223–224, 388–392 (2004).

Timing and spatial scale

Luminescence and magnetic susceptibility data where collected during multiple field trips during 2021-2023 and processed until 2025. Archaeological artifacts used for measuring cosmogenic 26Al and 10Be were collected by V.A. Ranov in 1976, 1979 (Lakhuti-I), 1981 (Kuldara), 1982, 1986 (Karatau), 1995, 1997 (Obi-Mazar), 2000 (Khonako-III), by T. Khujageldiev in 2021 (Kuldara), by A.A. Anoikin in 2021 (Lakhuti-IV) and stored at the Natural Museum of Tajikistan (see summary in Table S4).

Data exclusions

Cosmogenic 26Al and 10Be samples labeled LA293, OM67, KulPC11.1 and KulPC11.2 were excluded from the age-depth model as the samples represent modern samples with a 26Al/10Be ~ 6.8 (because the samples derive from landslides), while OP6, Laalu1s, Laalu3s, Laalu4.1, and Laalu4.2 were excluded because the uncertainty on the concentrations are so high that the age-depth model does not benefit from including them, Laalu6s was excluded as the Be concentration measured with ICP-OES was higher than spike value, and lastly, KA1573 and Ka4, were excluded as we don't focus on the site at Karatau because we have insufficient data from this location. These samples are included in table S4 for any future studies to use.

Reproducibility

Not relevant for our study.

Randomization

Not relevant for our study.

Blinding

Not relevant for our study as the results are objective.

Did the study involve field work? ☒ Yes ☐ No

Field work, collection and transport

Field conditions	Not relevant for our study. Our study relies on rock and sediment samples.
Location	Khovaling Loess Plateau, southern Tajikistan.
Access & import/export	We obtained export permits to export the rock and sediments samples. The sites are not protected.
Disturbance	The disturbance in the field was minimal and limited to extractions of rock and sediment samples.

Reporting for specific materials, systems and methods

We require information from authors about some types of materials, experimental systems and methods used in many studies. Here, indicate whether each material, system or method listed is relevant to your study. If you are not sure if a list item applies to your research, read the appropriate section before selecting a response.

Materials & experimental systems

n/a	Involved in the study
☒	☐ Antibodies
☒	☐ Eukaryotic cell lines
☐	☒ Palaeontology and archaeology
☒	☐ Animals and other organisms
☒	☐ Clinical data
☒	☐ Dual use research of concern
☒	☐ Plants

Methods

n/a	Involved in the study
☒	☐ ChIP-seq
☒	☐ Flow cytometry
☒	☐ MRI-based neuroimaging

Palaeontology and Archaeology

Specimen provenance	We obtained permission from the Tajik authorities (Natural Museum of Tajikistan, in Dushanbe) to collect and export the relevant samples.
Specimen deposition	Some samples were destructed, but the remaining sample material can be accessed at Aarhus University and DTU Risø, Denmark. Archaeological artefacts are available at the Natural Museum of Tajikistan in Dushanbe.
Dating methods	The Khovaling Loess profiles are dated using a combination of luminescence, cosmogenic nuclides, and magnetostratigraphy. This is specified in the main text and in the supplementary material.

☒ Tick this box to confirm that the raw and calibrated dates are available in the paper or in Supplementary Information.

Ethics oversight	No ethical approval was needed because our study only involves rock and sediment samples.

Note that full information on the approval of the study protocol must also be provided in the manuscript.

Plants

Seed stocks	Not relevant for our study.
Novel plant genotypes	Not relevant for our study.
Authentication	Not relevant for our study.
