# Supplementary material for: Absolute chronology of the Early Palaeolithic Karatau Culture in Central Asia
Source: Nat Commun. 2026 Jul 1;17:8217. doi: 10.1038/s41467-026-74938-5 (PMC13462899; doi:10.1038/s41467-026-74938-5)
Supplement: Supplementary file 5 [file 41467_2026_74938_MOESM8_ESM.docx]

Museum accession numbers of the lithic artefacts presented in figure 3 and 5 of “Absolute chronology of the Early Palaeolithic Karatau Culture in Central Asia”

The artefacts are located at the National Academy of Science of Tajikistan in Dushanbe. Please contact Tura Khudjageldiev ([tura959@mail.ru](mailto:tura959@mail.ru)) concerning access to archaeological samples.

**Fig 3.**

Pictures and 3D models of different types of lithic artefacts of the Karatau Kulture. 1-4 - Lakhuti-IV; 5-9 - Lakhuti-I; 10-13 – Karatau. 1, 9, 11 - transverse end-scrapers; 2, 5, 10, 13 - unifaces; 3 – orthogonal parallel core; 4 - radial core; 6 - chopper; 7, 12 – “citron” cores; 8 – unidirectional parallel core. Accession numbers:

1 - Л-IV-23. Р-4. №35

2 - Л-IV-23. Р-4. №38

3 - Л-IV-23. Р-4. №238

4 - Л-IV-23. Р-4. № 188

5. - Л-76. № 1112/422

6 - Л-76. № 62

7 - Л-76. № 474

8 - Л-76. № 1112/433

9 - Л-76. № 1112/353

10 - K-T. № 1112/1491

11 - K-T. № 1112/2937

12 - K-T. № 1043/81

13 - K-T. № 1112/2509

**Fig. 5**

Lithic artefacts found in PC 6. 1, 2 - Obi-Mazar PC4; 3-6 - Khonako-III; 7-11 - Lakhuti-I; 12, 15, 18 - Obi-Mazar PC6; 13, 14, 16, 17 - Karatau; 19 - Lakhuti-IV. 1, 6 - radial cores; 2-4, 7, 8, 14, 15 - unifaces; 5, 9, 16, 17 - choppers; 10 - transverse side-scraper; 11 - circular side-scraper; 12, 18 – single parallel cores; 13 – citron core; 19 - Тауасian point. Accession numbers:

1 - OM-IV. 1995. № 1112/3478

2 - OM-IV. 1997. № Ш1211

3 - Хонако. № ш2/28

4 - Хонако. № ш2/50

5 - Хонако. № ш2/165

6. - Хонако. № ш2/148

7 - Л-76. № 740

8 - Л-76. № 1112/977

9 - Л-95. № 1112/921

10 - Л-76. № 1112/353

11 - Л-76. № 1112/355 + Л-76. № 1112/364

12 - OM-VI-97. № 165

13 - K-T. № 1114/348

14 - K-T. № 1112/??? (erased)

15 - OM-VI. № 1066/6

16 - K-T. № 1112/1952

17 - K-T. № 1047/14

18 - OM-VI-97. № 16

19 - Л-IV-22. Р-3. № 12
